# A simulation study comparing anticholinergic drug use with polypharmacy for risk of death, dementia, and delirium in UK Biobank

**DOI:** 10.1093/gerona/glaf232

**Published:** 2025-10-23

**Authors:** Jure Mur, Lucy E Stirland, Graciela Muniz-Terrera, Anja K Leist

**Affiliations:** Centre for Clinical Brain Sciences, University of Edinburgh, Edinburgh, United Kingdom; Department of Social Sciences, Institute for Research on Socio-Economic Inequality (IRSEI), University of Luxembourg, Esch-sur-Alzette, Luxembourg; Centre for Clinical Brain Sciences, University of Edinburgh, Edinburgh, United Kingdom; Global Brain Health Institute, University of California San Francisco, San Francisco, California, United States; Centre for Clinical Brain Sciences, University of Edinburgh, Edinburgh, United Kingdom; Heritage College of Osteopathic Medicine, Ohio University, Athens, Ohio, United States; Department of Social Sciences, Institute for Research on Socio-Economic Inequality (IRSEI), University of Luxembourg, Esch-sur-Alzette, Luxembourg; (Medical Sciences Section)

**Keywords:** Simulation, Primary care, Prescribing, Medication, UK Biobank

## Abstract

**Background:**

Polypharmacy and use of anticholinergics are associated with adverse health outcomes. Because these 2 exposures covary, it is not known to what extent the detrimental health effects attributed to anticholinergic burden measured by anticholinergic burden scales (ABS) may be due to polypharmacy.

**Methods:**

Our aims were to evaluate the added effect beyond polypharmacy of (1) anticholinergic use and (2) scores according to existing ABS. We used linked data from the UK Biobank prospective cohort study to investigate drugs prescribed in primary care in the year 2015 (*n* = ∼200 000; median age = 65 years). We randomly sampled among drugs to create 2000 pseudoscales—drug burden scales designed to reflect the strength of the background effect of polypharmacy. We differentiated pseudoscales constructed to capture either general polypharmacy or putative anticholinergic polypharmacy. For each scale, we fitted logistic regression adjusted for confounders to assess associations between pseudoscales from each set and risk of death, dementia, or delirium. We also assessed 23 existing ABS by comparing them to the effects of pseudoscales that included the same numbers of drugs as each ABS.

**Results:**

Odds ratios for death, dementia, and delirium using anticholinergic-polypharmacy pseudoscales (median = 1.09-1.10) were greater than those of general-polypharmacy pseudoscales (1.05-1.06). The added effect of anticholinergic use beyond polypharmacy was greater in adjusted models and when prescribed to participants when they were older. 35%-90% of ABS exhibited stronger effects than most pseudoscales.

**Conclusions:**

The models show an anticholinergic effect of drugs beyond polypharmacy or drug count, but it is small, ABS-dependent, and varies between outcomes.

## Introduction

Anticholinergic burden scales (ABS) assign numerical values to drugs based on their presumed propensity to block muscarinic receptors. The sum of an individual’s values yields a weighted burden score—the anticholinergic burden—that can be used by clinicians or researchers to identify patients or populations at risk of adverse effects. Anticholinergic burden has been associated with negative long-term health outcomes, including death,^1^ dementia,^2^ delirium,^3^ falls,^4^ fractures,^5^ and hospitalizations.^6^

Polypharmacy, the concurrent use of multiple unique medications, is also associated with several negative health outcomes.^7–10^ Polypharmacy is usually defined as the concomitant use of ≥5 or ≥10 medications.^11^ However, given that the use of any drug involves the risk of adverse side effects, and a greater number of drugs allows for more drug–drug interactions, it is reasonable to assume a continuum, where the probability of patient harm increases with the number of medications taken. An additional prescribed drug has a non-zero probability to be anticholinergic; and because such drugs are commonly prescribed,^12–14^ this probability is substantially higher than zero. Thus, on average, an increase in polypharmacy also increases the anticholinergic burden. Some modeling studies have attempted to estimate the specifically anticholinergic effect by adjusting for the count of non-anticholinergic drugs.^15^ However, the increase in the number of prescribed anticholinergic drugs (henceforth referred to as *anticholinergic polypharmacy*) is itself a component of polypharmacy (henceforth referred to as *general polypharmacy*, to distinguish it from anticholinergic polypharmacy) and any estimate of an effect of anticholinergic burden will include the effect of this “hidden” polypharmacy. Thus, it is unclear to what extent the observed effect is a consequence of the number of prescribed drugs as opposed to the specific nature of anticholinergic drugs. In other words, is there an anticholinergic effect beyond polypharmacy?

To answer this question, one could compare the effect of anticholinergic burden to that of general polypharmacy and observe whether there is a difference. However, it is uncertain how the two respective lists of drugs should be constructed and how the drugs should be weighted to yield a valid comparison. To circumvent this issue, we used a simulation approach: we defined common parameters for general and anticholinergic prescribing and within those parameters through random sampling, we repeatedly constructed scales (hereafter referred to as pseudoscales). The first set of pseudoscales was sampled among all drugs to capture general polypharmacy, whereas the second set of pseudoscales sampled only among putatively anticholinergic drugs to capture specifically anticholinergic polypharmacy. For each pseudoscale, we calculated a drug burden score and its association with the outcomes of all-cause death, dementia, and delirium.

We hypothesized that any combination of drugs would be associated with adverse health outcomes due to polypharmacy. If that is the case, comparing the effect of anticholinergic use with the null—as in previous studies—produces an overestimate of the anticholinergic effect due to the effect of hidden polypharmacy. Our approach was to construct a new “polypharmacy null” in comparison to which the true effect of anticholinergic drugs could be more appropriately evaluated. We also created pseudoscales that represented the polypharmacy component of existing ABS and assessed the latter in their effect on the above outcomes relative to the background polypharmacy effect.

## Methods

### Sample

UK Biobank is a cohort study of ∼500 000 participants from England, Scotland, and Wales, whose ages ranged from 37 to 73 years during the first assessment between the years 2006 and 2010. For these participants, the UK Biobank acquired demographic and lifestyle information and evaluated data from cognitive tests and blood-based diagnostics. This information has also been linked to data on inpatient secondary hospital care (ie, participants admitted to hospital overnight), death records, and—for about half of participants—to primary care. The latter includes diagnoses and prescriptions recorded by general practitioners.^16^^,^^17^

### Prescription preparation

The initial sample consisted of 222 048 participants with 57 691 961 prescriptions from primary care. We removed prescription records that lacked content, those issued before the date of birth, and those issued in the future, assuming that these were erroneous. This led to the exclusion of <1% of prescriptions (­Figure S1). We matched prescriptions with generic drug names. Because the search for drugs in the sample and quality control of name matches could not be completely automated, we did not label all prescriptions. Instead, we used the British National Formulary (https://bnf.nice.org.uk/) to identify the most common character strings (>5000 total occurrences), drugs listed in ABS (see below), and all existing drug combinations of the above, thus labeling ∼95% of prescriptions. For assignment of anticholinergic potency, we identified 23 ABS that were available as lists of medicines with unambiguous numerical potency scores, updating a previous selection^18^ (Table S1).

**Figure 1.**
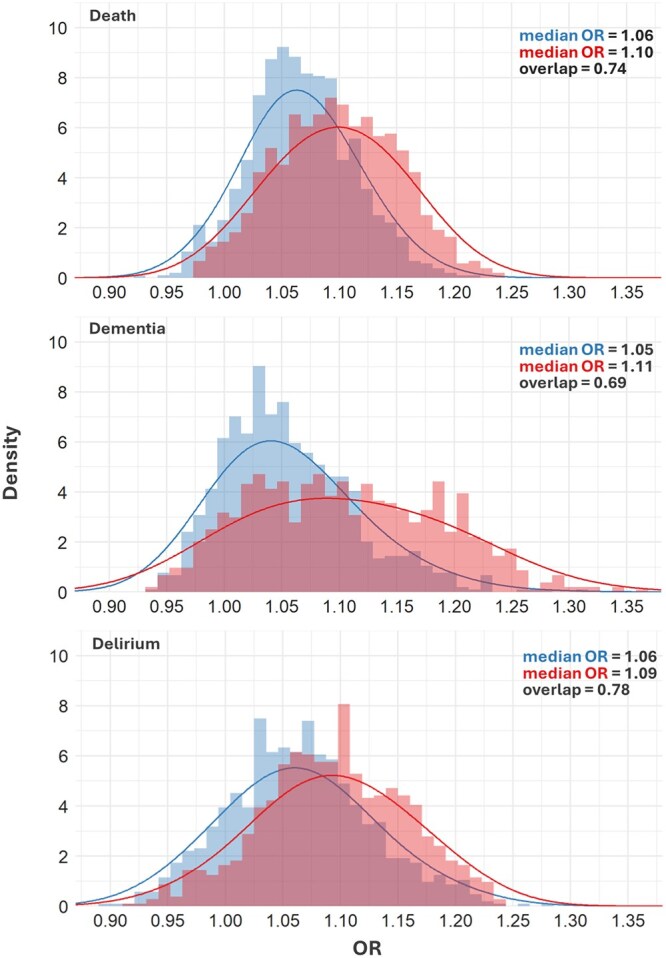
Histograms and density plots for effects of general (blue; left histogram in each panel) and anticholinergic (red; right histogram in each panel) across-sampling pseudoscales when estimating the effect of drug burden on death, dementia, or delirium. In the right-hand corner of each panel, the top median OR refers to general pseudoscales, the bottom median OR refers to anticholinergic pseudoscales. OR, odds ratio.

**Table 1.** Descriptive statistics for covariates used in models. For the descriptive statistics of other covariates specific to each outcome, see Table S6. The below numbers hold for the sample before the removal of outlier burden scores and before application of SMOTE.

	*N* (%)					
Outcome	Death		Dementia		Delirium	
	No (*n* = 182 538)	Yes (*n* = 11 968)	No (*n* = 157 756)	Yes (*n* = 2563)	No (*n* = 187 909)	Yes (*n* = 3102)
**Follow-up (years), median (Q1-Q3)**	6.8 (6.8-6.8)	4.2 (2.4-5.6)	6.8 (6.8-6.8)	4.1 (2.5-5.6)	6.8 (6.8-6.8)	4.5 (2.9-5.8)
**Age (years), median (Q1-Q3)**	64.7 (57.2-70.0)	71.0 (66.7-74.2)	65.0 (57.4-70.2)	72.7 (69.7-75.1)	65.0 (57.4-70.3)	72.4 (68.8-74.9)
**Female sex**	102 035 (55.9)	4973 (41.6)	86 295 (54.7)	1204 (47.0)	103 776 (55.2)	1348 (43.5)
**Data provider** ^a^						
***Missing***	1828 (1.0)	69 (0.6)	1150 (0.73)	8 (0.31)	1852 (1.0)	13 (0.41)
***England (Vision)***	16 332 (8.9)	897 (7.5)	15 828 (10.0)	267 (10.4)	16 551 (8.8)	360 (11.6)
***England (TPP)***	127 485 (69.8)	8182 (68.4)	124 473 (78.9)	2060 (80.4)	131 036 (69.7)	2210 (71.2)
***Wales***	16 591 (9.1)	1155 (9.7)	16 305 (10.3)	228 (8.9)	17 294 (9.2)	144 (4.6)
***Scotland*** ^b^	20 302 (11.1)	1665 (13.9)			21176 (9.2)	375 (12.1)
**Graduate degree**	60 337 (33.1)	2735 (22.9)	50.663 (32.1)	487 (19.0)	61 506 (32.7)	627 (20.2)
**Deprivation, median (Q1-Q3)**	−2.2 (−3.7-0.26)	−1.8 (−3.4-1.4)	−2.3 (−3.7-0.12)	−2.1 (−3.6-0.73)	−2.2 (−3.7-0.37)	−1.8 (−3.4-1.3)
**Alcohol consumption**						
***Daily or almost daily***	35 938 (19.7)	2713 (22.7)	32 167 (20.4)	528 (19.7)	37 430 (19.9)	673 (21.7)
***Three or four times a week***	43 106 (23.6)	2378 (19.9)	37 402 (23.7)	505 (19.7)	44 237 (23.5)	567 (18.3)
***Once or twice a week***	48 677 (26.7)	2808 (23.5)	41 562 (26.3)	613 (23.9)	49 918 (26.6)	690 (22.2)
***One to three times a month***	20 793 (11.4)	1192 (10.0)	17 717 (11.2)	253 (9.9)	21 269 (11.3)	318 (10.3)
***Special occasions***	20 190 (11.1)	1535 (12.8)	17 204 (10.9)	350 (13.7)	20 769 (11.1)	425 (13.7)
***Never***	13 834 (7.6)	1342 (11.2)	11 704 (7.4)	314 (12.3)	14 286 (7.6)	429 (13.8)
**Waist circumference (cm), median (Q1-Q3)**	89 (80-98)	95 (85-104)	90 (80.1-99.0)	92 (83-101.5)	90 (80-99)	95 (85-104)
**Smoking status**						
***Never smoker***	103 401 (56.6)	4755 (39.7)	87 690 (55.6)	1200 (46.8)	104 882 (55.8)	1308 (42.2)
***Former smoker***	61 564 (33.7)	5061 (42.3)	54 578 (34.6)	1125 (43.9)	64 189 (34.2)	1364 (44.0)
***Current smoker***	17 573 (9.6)	2152 (18.0)	15 488 (9.8)	238 (9.3)	18 838 (10.0)	430 (13.9)
**Physical activity**						
***None***	11 116 (6.1)	1392 (11.6)	9177 (5.8)	252 (9.8)	11 716 (6.2)	362 (11.7)
***Light***	6595 (3.6)	694 (5.8)	5886 (3.7)	139 (5.4)	6966 (3.7)	173 (5.6)
***Moderate***	145 631 (79.8)	9306 (77.8)	126 317 (80.1)	2068 (80.7)	149 808 (79.7)	2435 (78.5)
***Strenuous***	19 196 (10.5)	576 (4.8)	16 376 (10.4)	104 (4.1)	19 419 (10.3)	132 (4.3)
**Cerebrovascular disease**	4927 (2.7)	1147 (9.6)	4420 (2.8)	300 (11.7)	5516 (3.0)	356 (11.5)

Q1-Q3: interquartile range. SMOTE, synthetic minority oversampling technique.

a The name in parentheses refers to the data provider of primary care data of which there were two for participants from England.

b Participants from Scotland were excluded from analyses of dementia due to a lack of data on air pollution.

For each outcome, the population of interest was adults without previous experience of that outcome. For our primary analyses, we restricted the prescribing period to the year 2015, the end of which was time zero. We chose this year for 2 reasons. First, to maximize the completeness of the primary care record, which increases with time and ends at the time of extraction (years 2016-2017). Second, to maximize the age of the sample, as previous studies on this topic have mostly focused on older individuals. We inferred periods of continuous primary-care ascertainment as previously described.^19^ Follow-up for a participant ended when they experienced the outcome, were lost to monitoring, or when ascertainment ended. The latter was dependent on the nation-specific data provider of inpatient records and was on 31st October, 31st August, and 31st May 2022 for participants with inpatient data from England, Scotland, and Wales, respectively.

### Causal structure and variables

We formulated a causal structure for drug burden (Figure S2), defining confounders as assumed common causes of exposure and outcome based on the previous literature on risk factors for dementia,^20^ delirium,^21^^,^^22^ and death.^23^ The dementia outcome^24^ had been validated for its positive predictive value,^25^ whereas the delirium outcome^26^ was based on the ICD10 code F05, as previously.^27–29^ Death was ascertained by linkage to the death register. For residual polypharmacy (node B_0_ in ­Figure S2), we used the count of prescribed drugs that were not included in the calculation of the exposure scale (see below). We also included age, data provider (primary care computer system supplier), sex, education, socioeconomic deprivation, alcohol consumption, waist circumference, smoking status, physical activity, and history of cerebrovascular disease. Depending on the outcome, we also included further confounders. The details of all variables, including operationalization and UK Biobank field IDs are available as Tables S2-S4 and Text S1. Variables ascertained by self-report were measured during the first UK Biobank assessment between the years 2006 and 2010. The history of each disorder is coded as present if it occurred before the start of the prescription period.

**Figure 2.**
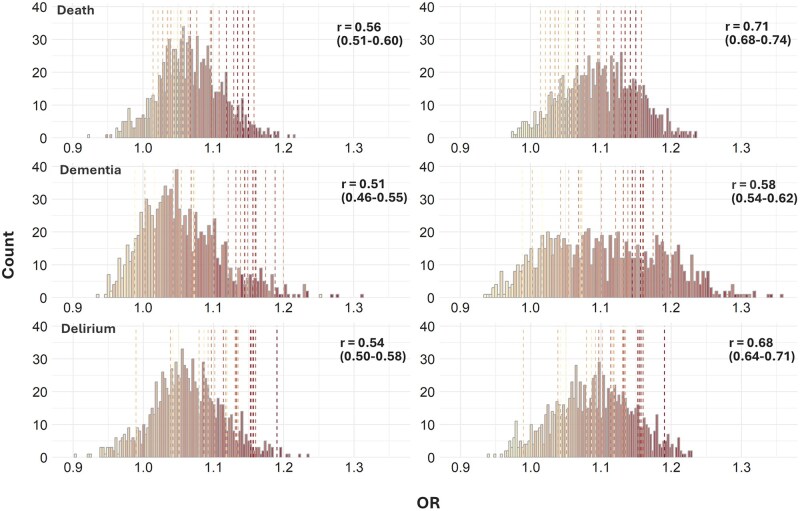
Histograms and heatmaps of effect sizes for general (left) and anticholinergic pseudoscales (right) for death, dementia, or delirium. The distributions are the same as presented in Figure 1, the colors/shades indicate the average scale size for a given group of effect sizes from 15 (yellow; light shade) to 150 (red; dark shade). The dotted lines represent the effect sizes for existing ABS and are identical between the right and left panels. The numbers in the top right corner of each graph represent Pearson’s correlation coefficient (and the 95% CI) for the correlation between the OR (indicated on the *x*-axis) and the size of the pseudoscales (indicated by the color). ABS, anticholinergic burden scales; CI, confidence interval; OR, odds ratio.

**Table 2.** Association of burden scores with the outcomes. The rows depict for all 3 outcomes the 95% SI, medians, and interquartile ranges of the ORs, and the results of the Wilcoxon Rank Sum test of the difference between the ORs between general and anticholinergic pseudoscales.

Outcome	Death		Dementia		Delirium	
Pseudoscale type	general	antichol.	general	antichol.	general	antichol.
**95% OR SI**	0.98-1.16	1.00-1.20	0.97-1.18	0.97-1.26	0.96-1.17	0.98-1.20
**Median (IQR)**	1.06 (0.06)	1.10 (0.08)	1.05 (0.08)	1.10 (0.13)	1.06 (0.06)	1.09 (0.08)
**Difference (*p*)**	<.001		<.001		<.001	

95% SI, 95% simulation intervals; antichol., anticholinergic; IQRs, interquartile ranges; OR, odds ratios.

### Scale construction

We used 2 different procedures to construct pseudoscales, “across-sampling” and “within-sampling.” Across-sampling was intended to separate the effects of anticholinergic polypharmacy as ascertained with a theoretical, generic ABS from those of general polypharmacy. The number of drugs to be included in pseudoscales was determined by repeated random draws from a discrete uniform distribution with the range bounded by the minimum and the maximum numbers of drugs prescribed in the prescription period (range = 15-150 for 2015) that were scored as anticholinergic across the 23 ABS. For each drug, the probability of being assigned a potency score was based on the frequency of that potency score across ABS (probabilities of assignment: 0.017, 0.25, 0.20, 0.52, and 0.009 for potency scores 4, 3, 2, 1, and 0.5, respectively). Within-sampling was performed separately for each ABS and was intended to generate pseudoscales comparable to that ABS. This was achieved by creating pseudoscales with the same number of included drugs for each ABS and the same distribution of potency scores as in the ABS. We generated 250 within-sampling pseudoscales for each of the 23 ABS, totaling 5750. The algorithm of pseudoscale construction is described in Text S2.

Both across-sampling and within-sampling were performed twice. First, among all drugs in the sample (general polypharmacy) and second among those drugs present in the sample that were considered anticholinergic by at least one ABS (anticholinergic polypharmacy). Because our aim was to distinguish the effects of anticholinergic drugs from those of polypharmacy (due to all drugs, including anticholinergics), the procedure of pseudoscale construction will have resulted in some overlap between anticholinergic and general polypharmacy pseudoscales; that is, many drugs will have been randomly allocated to some anticholinergic and some general pseudoscales. The total number of drugs that could be sampled was 525 for general polypharmacy and 214 for anticholinergic polypharmacy. Each scale (ABS or pseudoscale) was then used to calculate an annual burden score for each participant as had been done before.^14^^,^^15^^,^^18^ Briefly, for any participant, each prescription was linked to a medicine, which was assigned a numerical value as per the scale (or 0 if not present on the scale). These numerical values were then summed over the period of interest and an average annual cumulative burden was computed for that period. Drugs with ophthalmic, otic, nasal, or topical administration routes (∼12.5% of prescriptions) were assigned potency scores of 0, as previously.^15^^,^^30–32^

### Modeling

Drug burden scores and residual polypharmacy that exhibited unusually high values (>4 standard deviations above the mean) and observations missing values for any modeling variables were removed (Table S5). Numerical variables were standardized and centered to have a mean of 0 and standard deviation of 1. We used logistic regression and effect sizes were expressed in odds ratios (OR). Due to the high number of models, we randomly selected a subset of models (<0.5%) for performance evaluation. Burden scores generally did not show linear relationships with the log odds. Moreover, large residuals were often disproportionately represented among cases. Because of this, before running the models, we applied synthetic minority oversampling technique (SMOTE)^33^ (Text S2), which decreased the imbalance of residuals. The overlap between distributions of ORs was calculated by the kernel density estimation using the Sheather–Jones method.^34^ We used Python 3.11.5 and R 4.3.2 for analysis, and the code is available at https://github.com/JuM24/Pseudoscales_simulation.

### Sensitivity analyses

For the primary analyses, we present unadjusted and confounder-adjusted ORs, as well as ORs without the application of SMOTE. To test the robustness of our results, we repeated the models for the prescription period for the years 2004-2006 instead of 2015, assuming that result patterns would be similar but would show overall smaller effects due to the lower age of the sample. To incorporate length of follow-up and right-censoring, across-sampling pseudoscales were additionally evaluated using Cox proportional hazards models and competing risk models, where effects are presented as hazards ratios (HR) (for more details, see Text S2).

## Results

Among the ∼160 000-190 000 participants (numbers differ between models), ∼6.2% died, ∼1.6% were diagnosed with dementia, and ∼1.6% were diagnosed with delirium (Table 1, Table S6). In the year 2015, between 10% and 45% of participants—depending on the scale—were prescribed at least one anticholinergic drug. Among the latter, the median anticholinergic burden ranged from 4 to 18 across scales (Table S1). Among participants with complete data for all analytical variables, there was broad overlap in the distribution of variables between the subsample of participants with primary-care data (who were included in the analyses) and those without primary-care data (who were excluded from the analyses) (Table S7).

### Across-sampling

In across-sampling, the ORs of general pseudoscales ranged from 0.92 to 1.22 (death), from 0.94 to 1.31 (dementia), and from 0.90 to 1.24 (delirium). Odds ratios of anticholinergic pseudoscales ranged from 0.97 to 1.24 (death), from 0.93 to 1.36 (dementia), and from 0.94 to 1.23 (delirium). The 95% simulation intervals (SI) that express the values in which 95% of ORs are situated were of similar magnitudes for general and anticholinergic pseudoscales. On average, anticholinergic pseudoscales exhibited larger effect sizes than general pseudoscales (Table 2). The distributions of ORs for general and anticholinergic polypharmacy exhibited an overlap of 0.74, 0.69, and 0.78 for death, dementia, and delirium, respectively (Figure 1).

At *p* = .05, the association between drug burden and the outcome was significantly positive for 909/1000, 772/1000, and 866/1000 general pseudoscales for death, dementia, and delirium, respectively. For anticholinergic pseudoscales, the corresponding numbers were 955/1000, 874/1000, and 917/1000. Thus, the associations between drug burden and the outcome exhibited a 5% (death), 13% (dementia), and 6% (delirium) higher probability of being significant if the burden scale was constructed using only putatively anticholinergic drugs as opposed to all drugs. Effects of the 23 ABS-derived scores ranged from 1.01 to 1.16, 0.99 to 1.20, and 0.99 to 1.19 on the risk of death, dementia, and delirium, respectively (Table S8). The OR correlated with the scale size (ie, number of drugs included on a scale) for all types of scales but was greatest for ABS [*r* = 0.82 (95% CI = 0.62-0.92), *r* = 0.72 (95% CI = 0.44-0.87), and *r* = 0.77 (95% CI = 0.53-0.90)] for death, dementia, and delirium, respectively. This correlation was smaller for pseudoscales (Figure 2).

### Within-sampling

**Figure 3.**
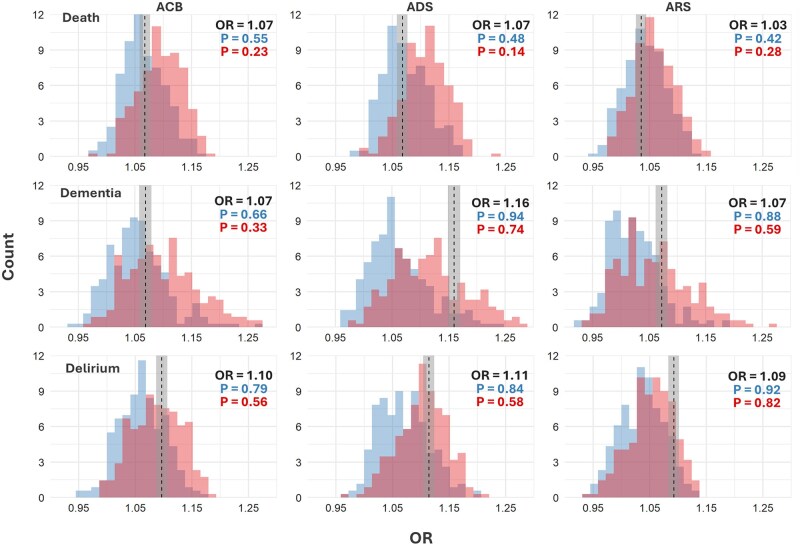
Histograms of effect sizes for 3 exemplar ABS (dashed line) against distributions of general (blue; left histogram in each panel) and anticholinergic (red; right histogram in each panel) within-sampling pseudoscales when estimating the effects of drug burden on death (top row), dementia (middle row), or delirium (bottom row). The shaded vertical rectangle in each panel represents the 95% CI. Depicted are the results for the frequently investigated Anticholinergic Cognitive Burden Scale (ACB),^41^ the Anticholinergic Drug Scale (ADS),^42^ and the Anticholinergic Risk Scale (ARS).^31^ The plots display in the top right corner the ORs for the 3 ABS and the cumulative probability at the ORs (top/blue: general; bottom/red: anticholinergic). The latter expresses the proportion of ORs of general or anticholinergic pseudoscales that are smaller than the OR of the ABS. The plots for all other scales are in Figure S3. ABS, anticholinergic burden scales; CI, confidence interval; OR, odds ratio.

Among pseudoscales, 70%, 91%, and 96% of general and 35%, 74%, and 87% of anticholinergic pseudoscales for death, dementia, and delirium, respectively, exhibited weaker effects than their corresponding ABS. The range in differences between the ORs of ABS and the median ORs of general polypharmacy were −0.03 to 0.09 for death, −0.03 to 0.17 for dementia, and −0.05 to 0.10 for delirium. The range in differences between the ORs of ABS and the median ORs of anticholinergic polypharmacy was −0.05 to 0.06, −0.06 to 0.14, and −0.07 to 0.07 for death, dementia, and delirium, respectively. ABS that exhibited the strongest effects beyond polypharmacy were DRS for death, YS, AAS, and AEC for dementia, and AAS, DDS, and AEC for delirium (Figure 3, Figure S3, Table S9).

### Sensitivity analyses

The results were similar when SMOTE was not applied, although the SIs were narrower and the difference between general and anticholinergic polypharmacy was smaller (by OR = 0.031-0.034). In the unadjusted models, the effects were larger by OR = 0.25-0.41, and the difference between general and anticholinergic polypharmacy was smaller (OR = 0.016-0.018). Similar patterns of results were observed in time-to-event analyses, both for the cause-specific and the subdistribution hazards (Table S8). See Figure S4 for Kaplan–Meier curves comparing the effects of general and anticholinergic pseudoscales over the follow-up period. In analyses using the 2004-2006 prescribing period, effects were lower for unadjusted (by OR = 0.11-0.14) and adjusted models (by OR = 0.002-0.08) compared to the 2015 period. Further, the difference between general and anticholinergic polypharmacy was smaller for both unadjusted (OR ≤0.005) and adjusted (OR = 0.002-0.03) models compared to the 2015 period (Table S8). The relative effects of ABS after within-sampling mostly did not show consistent effects when the analyses were unadjusted or when the 2004-2006 prescribing period was used (compared to 2015) (Table S9).

## Discussion

The aim of our study was to determine the extent to which anticholinergic use and burden scores according to ABS exhibit effects on the risks of adverse long-term health outcomes that are not attributable to polypharmacy alone. We found that regardless of the exact model specifications, over half of pseudoscales consistently exhibited positive associations with death, dementia, and delirium, despite them not being based on any theoretical considerations of drug mechanism of action. The strength of the effect was moderately positively associated with the number of drugs included on the scales. Our findings demonstrate that if one combines enough drugs into a score, the effect between the latter and the risk of death, dementia, or delirium is highly likely to be positive. This raises questions about the utility of reporting findings of such associations without contrasts with valid non-null comparators.

Our comparator for the effect of anticholinergic polypharmacy was general, nonspecific, polypharmacy. Its median effect was halfway between the zero and the median of the effect of scales of anticholinergic polypharmacy. In other words, about half of the effect of anticholinergic use may be due to polypharmacy, with the rest attributable to anticholinergic drugs ­specifically. The above was true especially when the models were adjusted for confounders, underlining the importance of removing residual confounding when assessing the effects of drug burden.

Our results demonstrate that anticholinergic drugs, do on average, exhibit effects beyond polypharmacy. However, in the clinic and in research, we do not apply generic pseudoscales of anticholinergic use but use existing ABS that apply numerical weights based on expert evaluation to yield anticholinergic burden scores. When we compared the effects of the latter to polypharmacy comparators, most ABS exhibited stronger effects than the median effect of polypharmacy. However, these relative effects varied strongly between ABS. Further, the effect size for a scale for some outcome—compared to polypharmacy—did not necessarily correspond to its effect size for a different outcome. This indicates the need to develop risk scales specific for each outcome. Moreover, considering that all ABS are supposed to measure the same construct, such ­variability in effects challenges the validity of at least some commonly used ABS.

Anticholinergic burden scales are meant to capture a construct that is at best poorly measurable,^35^ and that combines drugs prescribed for dozens of indications. This precludes the use of appropriate comparators for the use of active-comparator, new-user designs^36^^,^^37^ that can be applied to determine causal effects in observational studies. Deprescribing trials—another approach to study causality in pharmacoepidemiology—are feasible with anticholinergic drugs, but do not decouple anticholinergic prescribing from polypharmacy, which was the goal of the present study. Our study was not intended to determine the absolute causal effect of any group of drugs or tool of drug use and to isolate it from residual or unmeasured confounding. An effect specific to anticholinergic drugs as is suggested by our results does not provide evidence for any specific mechanism. The identified effect is not due to polypharmacy but could be due to an antimuscarinic effect (direct causation), a more general effect of individual drugs or drug classes common in ABS, or an effect of residual confounding. A further exploration of these mechanisms should be a topic of future research.

### Strengths and limitations

Our study has several strengths. First, this is the first study to systematically distinguish between the effects of polypharmacy and anticholinergic drugs. Second, the study is methodologically robust as we adjusted for multiple potential confounders based on informed assumptions about the causal relationships between the studied variables, allowing a structured way of addressing the risk of bias. Third, the sample size was larger than in most other studies of anticholinergic burden, included a substantial number of cases, and the estimated effect sizes of commonly used ABS were compared against a large set of effect sizes from pseudoscales constructed through different approaches.

We acknowledge the possibility of residual confounding, especially confounding by indication and healthcare utilization. However, we assumed that these biases were present to the same extent in both sets of pseudoscales (general vs anticholinergic). We used UK Biobank because of the large sample size and continuous access to healthcare records, but the participants in our study were healthier than the target population,^38^ and exhibited relatively limited follow-up between prescribing ascertainment and right-censoring. Thus, the relative importance of anticholinergic prescribing vs polypharmacy may differ for longer follow-up periods. Moreover, they may have been prevalent (as opposed to incident) users of medications. We also had no information on drug adherence and implicitly assumed complete adherence for all analyses. Further, although we used the same inpatient ICD-codes to ascertain delirium diagnoses as previous authors using data from UK Biobank,^27^^,^^28^ we are unaware of any studies confirming the diagnostic accuracy of these codes. Also, due to the unavailability of primary-care data after the years 2016/2017, we did not use primary-care records to ascertain dementia, which likely reduced the sensitivity of dementia ascertainment. Next, we assumed that scale scores were additive and that linear relationships existed between scale scores and the risks of the outcomes. Despite the ubiquity of such assumptions in previous work, they may not hold.^39^ We also used scales to assess the effect of cumulative longitudinal use of drugs. Although this approach has been applied in some previous studies,^14^^,^^15^^,^^18^ most ABS are not intended to be used in this way. Moreover, the measure of polypharmacy resulting from a cumulative count may not necessarily reflect the number of unique drugs prescribed to an individual, but the total number of drugs (unique or not). However, we chose this approach because it captured not only the drugs prescribed to participants but also their frequency/amount, which better reflects the assumed biological mechanism of drug load and muscarinic inhibition. Finally, due to the relatively young age of the participants, we could not assess anticholinergic prescribing in the oldest individuals, often identified as those most at risk of drug side effects.^40^

In conclusion, without a valid polypharmacy control, the positive effect of a drug score on an outcome is uninformative. Although drugs with presumed antimuscarinic activity do exhibit effects on the risk of death, dementia, and delirium, the prescribing frequency/volume, irrespective of anticholinergic load, may be contributing a substantial proportion of the observed effect. Finally, the effects of existing ABS are variable and inconsistent across outcomes, and their validity and/or utility should be further scrutinized.

## Supplementary Material

glaf232_Supplementary_Data

## Data Availability

UK Biobank data are available to all approved researchers directly from UK Biobank. The code to process and analyze the data is available at: https://github.com/JuM24/Pseudoscales_simulation
