# Supplementary material for: A simulation study comparing anticholinergic drug use with polypharmacy for risk of death, dementia, and delirium in UK Biobank
Source: J Gerontol A Biol Sci Med Sci. 2025 Oct 23;80(12):glaf232. doi: 10.1093/gerona/glaf232 (PMC12643546; doi:10.1093/gerona/glaf232)
Supplement: glaf232_Supplementary_Data [file glaf232_supplementary_data.zip › supplement_plain.docx]

**A simulation study comparing anticholinergic drug use against polypharmacy on death, dementia, and delirium**

**Supplementary material**

**Mur, Jure PhD^^[[1]](#footnote-1)^,^[[2]](#footnote-2)^,*^, Stirland, Lucy E.** **MBChB PhD MRCPsych^1,^[[3]](#footnote-3)^^, Muniz-Terrera, Graciela PhD^1,^[[4]](#footnote-4)^^, & Leist, Anja K. PhD^2^**

**Table of contents**

Suppl. Figure 1 3

Suppl. Table 1 4

Suppl. Figure 2 5

Suppl. Table 2 6

Suppl. Table 3 9

Suppl. Text 1 10

Suppl. Text 2 12

Suppl. Table 5 13

Suppl. Table 6 14

Suppl. Table 7 17

Suppl. Figure 3 19

Suppl. Figure 4 24

Supplementary References 27


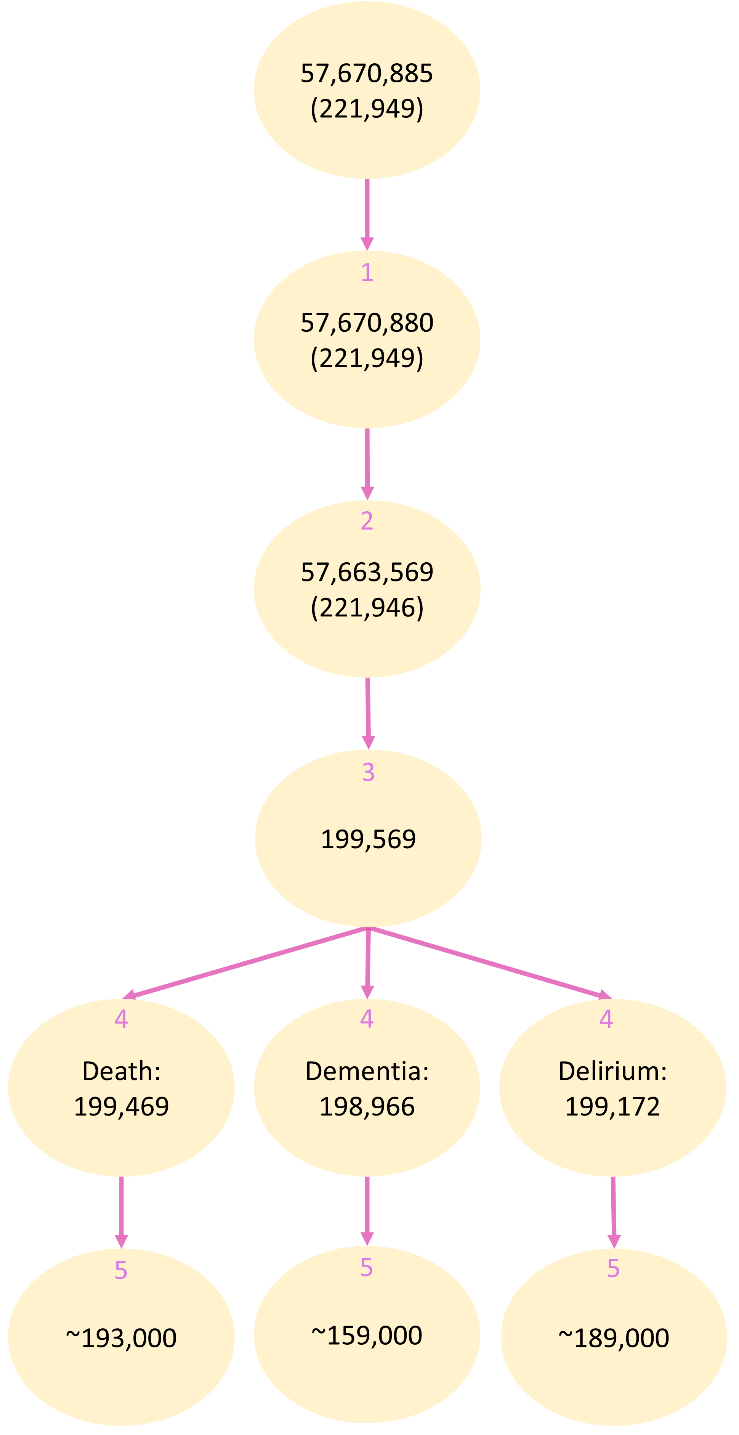
**Suppl. Figure 1**: changes in sample size during data cleaning. In each ellipse, the upper row represents the number of individual observations, the second row represents the number of participants. Where only a single row is present, participants are individual observations. The data cleaning steps are (1) removal of prescriptions missing their contents; (2) removal of invalid dates; (3) transformation from a prescription-based dataset into an id-year dataset and the removal of participants with no data for the year 2015 (either lost to follow-up before 2015 or only added to dataset after 2015); (4) removal of observations of the outcome that occurred before or during the year 2015, and (5) removal of observations with incomplete data for any of the covariates used in the model. The final number of observations for the modelling (after step 5) is the average across scales, because the number of removed outlier observations for the exposure (drug burden) differed between the scales.

**Suppl. Table 1**: some properties of the ABS used in the analysis. Two scales were updated since the initial publication (Aging Brain Care, 2012; Carnahan, 2014, personal communication on 21.10.2019) ^1,2^. The table also displays the scale size (i.e., the number of drugs with a score of >0 that were included in the scale and present in the sample in the year 2015), the proportion of participants with a burden score above zero, and the median burden score among participants with burden scores above zero. The last two rows display these statistics for pseudoscales. For general and anticholinergic pseudoscales, the median across the two pseudoscales groups, respectively, is shown for both the proportions and the median burden score values. This ABS list was updated from a list published previously^3^.

| **First author** | **Scale name** | **Year of publication** | **Size** | **Prop. >0** | **Median** |
| --- | --- | --- | --- | --- | --- |
| Summers^4^ | Drug Risk Number (DRN) | 1978 | 41 | 0.25 | 12 |
| Han^5^ | Clinician-rated Anticholinergic Scale (CrAS) | 2001 | 51 | 0.27 | 7 |
| Ancelin^6^ | Anticholinergic Burden Classification scale (ABC) | 2006 | 17 | 0.10 | 14 |
| Carnahan^2^ | Anticholinergic Drug Scale (ADS) | 2006 (2014) | 96 | 0.28 | 7 |
| Chew^7^ | Anticholinergic Activity Scale (AAS) | 2008 | 35 | 0.37 | 4 |
| Cancelli^8^ | Cancelli’s Anticholinergic Burden Scale (CABS) | 2008 | 13 | 0.12 | 18 |
| Rudolph^9^ | Anticholinergic Risk Scale (ARS) | 2008 | 40 | 0.19 | 7 |
| Ehrt^10^ | Revised Anticholinergic Activity Scale (AAS-r) | 2010 | 23 | 0.25 | 9 |
| Sittironnarit^11^ | Anticholinergic Loading Scale (ALS) | 2011 | 44 | 0.32 | 7 |
| Boustani^1^ | Anticholinergic Cognitive Burden (ACB) | 2008 (2012) | 80 | 0.29 | 8 |
| Sumukadas^12^ | Modified Anticholinergic Risk Scale (m-ARS) | 2013 | 57 | 0.20 | 8 |
| Durán^13^ | Durán Scale (DS) | 2013 | 74 | 0.28 | 6 |
| Hefner^14^ | Delirogenic Risk Scale (DRS) | 2015 | 88 | 0.43 | 12 |
| Nguyen^15^ | Drug Delirium Scale (DDS) | 2016 | 81 | 0.27 | 6 |
| Bishara^16^ | Anticholinergic effect on cognition scale (AEC) | 2017 | 56 | 0.24 | 7 |
| Briet^17^ | Anticholinergic impregnation scale (AIS) | 2017 | 109 | 0.38 | 8 |
| Kiesel^18^ | German Anticholinergic Burden Scale (GABS) | 2018 | 134 | 0.45 | 9 |
| Nery^19^ | Brazilian anticholinergic activity drug scale (BAAS) | 2019 | 100 | 0.37 | 8 |
| Jun^20^ | Korean Anticholinergic Burden Scale (KABS) | 2019 | 87 | 0.32 | 7 |
| Kable^21^ | Modified Anticholinergic Burden Scale (mACB) | 2019 | 72 | 0.33 | 8 |
| Ramos^22^ | CRIDECO Anticholinergic Load Scale (CALS) | 2020 | 150 | 0.43 | 9 |
| Al Rihani^23^ | AntiCholinergic and Sedative Burden Catalog (ACSBC) | 2021 | 133 | 0.44 | 9 |
| *Yamada^24^ | Yamada’s scale (YS) | 2023 | 55 | 0.27 | 9 |
|  | General pseudoscales |  | 83 | 0.39 | 9 |
|  | Anticholinergic pseudoscales |  | 83 | 0.35 | 9.5 |

*Note: Fluticasone fluorate and fluticasone propionate are scored differently according to this scale. Due to difficulties in distinguishing the two drugs in the sample, both were classified as having the same potency of 1 on this scale.

**Suppl. Figure 2:** simplified directed acyclic graphs (DAGs) constructed using *dagitty* (<https://dagitty.net>)^25^ for the assumed causal relationships between anticholinergic drugs and an idealised health outcome Y. For simplicity and illustration purposes, the DAGs do not depict any other common causes of exposure and outcome except underlying health conditions (U) and the total number of prescribed drugs (total polypharmacy, B). B can be decomposed into the count of anticholinergic drugs (B_1_) and the count of non-anticholinergic drugs (B_0_) such that B = B_0_ + B_1_. **Left**: no adjustment for confounders. **Right**: adjustment for B_0_ and U.

The exposure (anticholinergic burden, X) is determined by B_1_ and the potency scores assigned to each drug (not depicted). To determine the causal effect of X on Y, we need to close the backdoor paths between X and Y (**left**). However, while we can adjust for B_0_ and U (**right**), we cannot adjust for B_1_ due to the high collinearity between X and B_1_. Thus, the cause of the effect – (anticholinergic) polypharmacy or anticholinergic burden – is unclear.


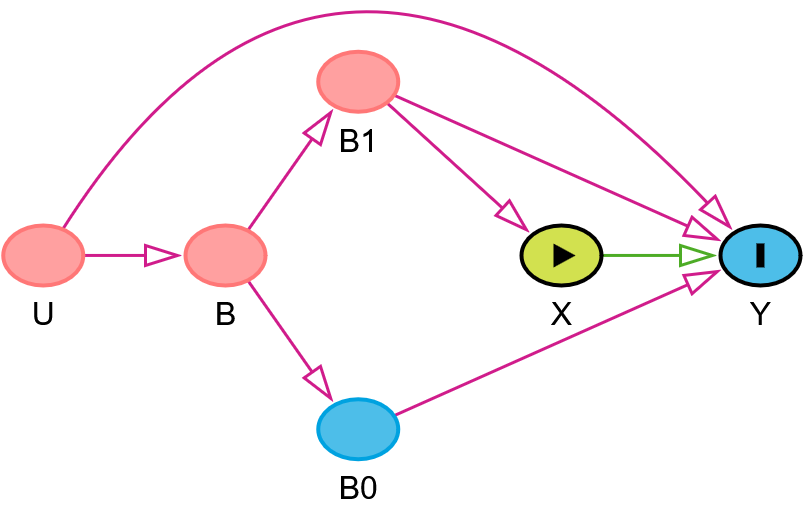

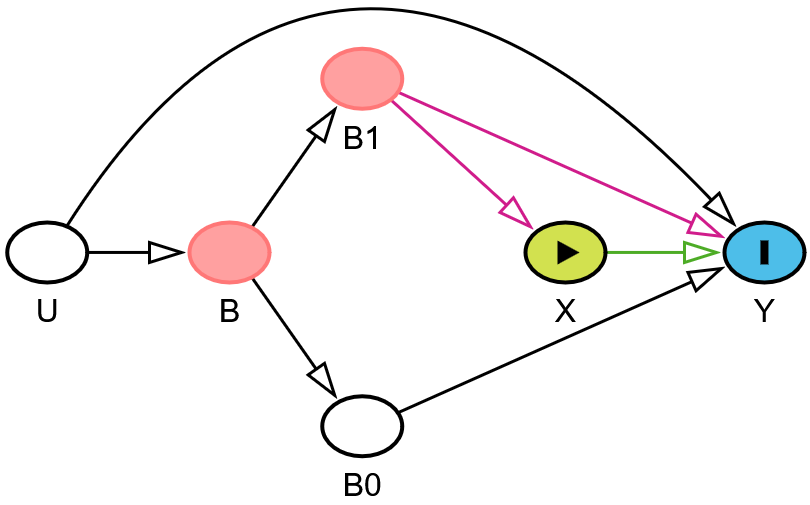


Legend: X: anticholinergic drug burden, Y: outcome, B: polypharmacy, B_1_: anticholinergic polypharmacy, B_0_: non-anticholinergic polypharmacy, U: underlying disorders. Causal paths are represented with arrows; red arrows signify biasing paths, the green arrow is the causal paths from the exposure to the outcome, white arrows are paths that do not confound the estimation of the causal path between the exposure and the outcome. Green, blue, and red ellipses signify ancestors of exposure, ancestors of outcome, and ancestor of exposure and outcome, respectively.

**Suppl. Table 2**: variables used in our analyses. The individual columns give the names of the variables, the field IDs in the UK Biobank showcase (<https://biobank.ndph.ox.ac.uk/ukb/>) used to derive the variables, the role of the variables in the models, and brief descriptions.

| **Name** | **Field ID** | **Role** | **Description** |
| --- | --- | --- | --- |
| *Drug burden | 42039 | predictor (all) | *drug_name* column in record table 1062 |
| Death | 40000 | outcome |  |
| Dementia | 42018 | outcome, covariate (death, delirium) |  |
| Delirium | 130846 | outcome |  |
| *Data provider | 42039 | covariate (all) | *data_provider* column in record table 1062 |
| Age | 34, 52 | covariate (all) | date of birth approximated from month and year of birth. |
| Sex | 31 | covariate (all) | binary variable |
| Non-scale polypharmacy | 42039 | covariate (all) | *drug_name* column in record table 1062 |
| *Education | 6138 | covariate (all) |  |
| *Deprivation | 189 | covariate (all) |  |
| *General cognitive ability | 20016, 20018, 20023, 399, 4282 | covariate (dementia, delirium) |  |
| *Air pollution | 24003, 24004, 24006 | covariate (dementia) |  |
| *Alcohol use frequency | 1558 | covariate (all) |  |
| Waist circumference | 48 | covariate (all) |  |
| Smoking status | 20116 | covariate (all) | three categories: smokers, non-smokers, previous smokers |
| *Physical activity | 6164 | covariate (all) |  |
| Mood disorder | 130890, 130892, 130894, 130896, 130898, 130900, 130902 | covariate (dementia, delirium) |  |
| Diabetes | 130706, 130708, 130710, 130712, 130714 | covariate (dementia, death) |  |
| Hypertension | 131286 | covariate (dementia) |  |
| Hyperlipidaemia | 130814 | covariate (dementia) |  |
| Psychotic disorder | 130874, 130876, 130878, 130880, 130882, 130884, 130886, 130888 | covariate (delirium) |  |
| Visual impairment | 131212 | covariate (delirium) |  |
| *Hearing impairment | 2247, 2257, 131258, 131260, 20019, 20021, 41270, 41280, 41271, 41281, category ID 2006, 42040 | covariate (dementia, delirium) |  |
| Sleep disorder | 131060, 130920 | covariate (delirium) |  |
| Vascular CNS disorder | 42006, 131370, 131372, 131374, 131376, 131378 | covariate (dementia, delirium) |  |
| Inflammatory CNS disorder | 131000, 130992, 130994, 130996, 130998, 131002, 131004, 131006, 131008, 131010 | covariate (dementia, delirium) |  |
| Atrophic CNS disorder | 131012, 131014, 131016, 131018, 131020, 42028 | covariate (dementia, delirium) |  |
| Movement CNS disorder | 131022, 131024, 131026, 131028, 131030, 131032 | covariate (dementia, delirium) |  |
| Demyelinating CNS disorder | 131042, 131044, 131046 | covariate (dementia, delirium) |  |
| Paroxysmal CNS disorder | 131048, 131050, 131056, 131058 | covariate (dementia, delirium) |  |
| Brain tumour | 40005, 40006, 40013 | covariate (dementia, delirium) | for ICD-codes, see **Suppl. Table 3** |
| *Traumatic brain injury (TBI) | 41270, 41280, 41271, 41281, category ID 2006, 42040 | covariate (dementia, delirium) |  |
| Other CNS disorder | 131038, 131040, 131100, 131110, 131112, 131114, 131116, 131120 |  |  |
| Endocrinopathy | 130690, 130692, 130694, 130696, 130698, 130700, 130702, 130704, 130718, 130720, 130722, 130724, 130726, 130728, 130730, 130732, 130734, 130736, 130738, 130742, 130744, 130746, 130748, 40005, 40006, 40013 | covariate (delirium) | includes endocrine cancer in **Suppl. Table 3** |
| Nutritional deficiency | 130750, 130752, 130756, 130758, 130760, 130762, 130764, 130766, 130768, 130770, 130772, 130774, 130776, 130778, 130780, 130782, 130784, 130786, 130788 | covariate (delirium) |  |
| Metabolic disorder | 130798, 130800, 130802, 130806, 130808, 130810, 130812, 130814, 130816, 130818, 130820, 130822, 130824, 130826, 130828, 130830, 130832, 130834 | covariate (delirium) |  |
| Cerebrovascular disease | 131360, 131362, 131364, 131366, 131368, 131370, 131372, 131374, 131376, 131378 | covariate (all) |  |
| Chronic lower respiratory disease | 131484, 131486, 131488, 131490, 131492, 131494, 131496, 131498 | covariate (death) |  |
| Liver disease | 131658, 131660, 131662, 131664, 131666, 131668, 131670 | covariate (death) |  |
| Influenza or pneumonia | 131438, 131440, 131442, 131444, 131446, 131448, 131450, 131452, 131454, 131456 | covariate (death) |  |
| Ischaemic heart disease | 131296, 131298, 131300, 131302, 131304, 131306 | covariate (dementia, death) |  |
| Colon cancer | 40005, 40006, 40013 | covariate (death) | for ICD-codes, see **Suppl. Table 3** |
| Prostate cancer | 40005, 40006, 40013 | covariate (death) | for ICD-codes, see **Suppl. Table 3** |
| Lung cancer | 40005, 40006, 40013 | covariate (death) | for ICD-codes, see **Suppl. Table 3** |
| Breast cancer | 40005, 40006, 40013 | covariate (death) | for ICD-codes, see **Suppl. Table 3** |
| Ovarian cancer | 40005, 40006, 40013 | covariate (death) | for ICD-codes, see **Suppl. Table 3** |
| *Social isolation | 709, 1031, 6160 | covariate (dementia, delirium) |  |
| Loneliness | 2020 | covariate (dementia, delirium) | binary variable |
| *Depressive mood | 2050 | covariate (dementia) |  |
| Date of loss to follow-up | 191 |  |  |

**Note: variables that were either recoded or otherwise transformed and for which the explanation is not provided in the main text, are described in further detail in* ***Suppl. Text 1****.*

**Suppl. Table 3**: ICD-codes for diagnoses of cancer which were unavailable as derived variables (first occurrences or algorithmically-defined outcomes) in UK Biobank. We instead used the UK Biobank cancer register (field IDs 40005, 40006, 40013) which is linked to UK national cancer registries.

| **Disorder** | **ICD10 codes** | **ICD9 codes** |
| --- | --- | --- |
| Colon cancer | C18, C180-189, C19-21, C210-212, C218 | 153, 1530-1539, 154, 1540-1543, 1548, 2303, 2304 |
| Prostate cancer | C61 | 185, 2365 |
| Lung cancer | C33, C34, C340-343, C348, C349 | 162, 1620, 1622, 1623, 1624, 1625, 1628, 1629, 2357 |
| Breast cancer | C50, C500-506, C508, C509 | 174, 1740-1746, 1748, 1749, 2330 |
| Ovarian cancer | C56 | 83, 1830, 1832, 184, 1840, 1841, 1844, 1848, 1849, 1986, 2362 |
| Brain tumour | D32, D320, D321, D329, D33, D331-334, D337, C70, C700, C709, C71, C710-719, C793 | 225, 2258, 2259, 2250-2254, 1921, 191, 1910, 1911, 1912, 1916-1919 |
| Endocrine cancer | C25, C250-254, C257-259, C73, C74, C740, C741, C749, C75, C750-755, D093, D34, D35, D351, D352, D44, D440-440 | 157, 1570, 1572, 1574, 1579, 193, 194, 1940, 1941, 1943, 1944, 1949, 226, 227, 2273, 2279, 2370 |

**Suppl. Text 1**: description of selected variables used in the analyses. When we write *inpatient diagnoses*, we are specifically referring to category ID 2002 (field IDs 41270, 41280, 41271, 41281) where diagnostic codes were mapped to dates in the summary diagnoses tables. When we write *primary care*, we are specifically referring to field ID 42040 that contains GP clinical event records. To query the latter, we used *readv2* and *readv3* codes provided by the NHS Digital Technology Reference Data Update Distribution (<https://isd.digital.nhs.uk/trud/user/guest/group/0/pack/9>).

- **Drug burden:** prescriptions were available from ~1990 until May 2017 for Scotland (EMIS/Vision), September 2017 for Wales, June 2017 for England (Vision) and August 2016 for England (TPP) ^26^.
- **Data provider**: each prescription is associated with a data provider (England: TPP, Vision; Scotland: EMIS/Vision, Wales: EMIS/Vision). When the data were transformed into the id-year format and restricted to the year 2015, the most frequent data provider for that period was chosen for each participant. For participants for which periods of continuous electronic health record ascertainment were inferred^27^, but no prescriptions were prescribed in the year 2015, the data provider was imputed by choosing that data provider which appeared closest in time to the year 2015. This imputed the data provider for 30,638 participants. Because some participants for whom the period of continuous health record ascertainment were inferred did not receive prescriptions (n = 1,941), those participants were classed as a separate group. In the sensitivity analysis where prescriptions in the years 2004-2006 were analysed, data providers were imputed in an analogous way, but participants from the Scottish data providers were excluded from the analyses for all three outcomes (as opposed to just dementia as in the main analysis). This was done due to a system-wide block of prescription records for Scotland before the year 2012^26^ and a consequent failure to assign many prescriptions to Scottish participants in that period. This would have led to a much lower prescription count for Scottish participants if the latter were retained in the analyses.
- **Education**: self-report on the ascertained qualifications was recorded in six categories: “*college or university degree*, *A levels/AS levels or equivalent*, *O levels/GCSEs or equivalent*, *CSEs or equivalent*, *NVQ or HND or HNC or equivalent*, and *other professional qualifications eg: nursing, teaching*. We recoded the classification into a binary variable, thus distinguishing between participants with and without a college degree.
- **Socioeconomic deprivation**: the Townsend deprivation index^28^ was provided by UK Biobank. The index is calculated based on the national census that preceded the participants’ joining UK Biobank. It scores several socioeconomic indicators and sums them up. The scores are then standardized, and each participant is assigned a score corresponding to their geographical area.
- **General cognitive ability**: a factor of general intelligence was calculated as before^3,29^. In short, we fitted a confirmatory factor analysis in a structural equation modelling framework using tests of visual declarative memory, processing speed, and – for a subsample of participants – tests of working memory, prospective memory, and verbal and numerical reasoning.
- **Air pollution**: a principal component analysis (PCA) was run on pollution due to nitrogen oxides (year 2010), nitrogen dioxide (year 2010), and particulate matter (pm_25_; year 2010). The pollutants were chosen due to having been previously associated with dementia^30^. We used the first principal component. Due to a lack of pollution data for participants in Scotland, the analysis for dementia was performed only on participants from England and Wales.
- **Alcohol use**: alcohol intake frequency was recorded in six categories: *daily or almost daily*, *three or four times a week*, *once or twice a week*, *one to three times a month*, *special occasions only*, and *never*.
- **Physical activity**: the types of physical activity undertaken in the four weeks before the assessment; recorded in six categories: *walking for pleasure (not as a means of transport)*, *other exercises (eg: swimming, cycling, keep fit, bowling)*, *strenuous sports*, *light DIY (eg: pruning, watering the lawn)*, *heavy DIY (eg: weeding, lawn mowing, carpentry, digging)*, and *none of the above*. We recoded the variable into three categories as previously: low (light DIY or none), medium (walking for pleasure, other exercises, or heavy DIY), and high (strenuous sports) ^31,32^.
- **Hearing impairment**: hearing impairment was identified based on self-report, objective hearing test during the assessment, and medical records. For self-report, hearing impairment was determined if the participant indicated to have both difficulties with their hearing and difficulties with their hearing in the presence of background noise. For the objective hearing assessment, hearing impairment was determined if the speech-reception-threshold (SRT) value for the better ear was above -5.5; this threshold was chosen based on previous studies^33,34^. For medical health records, hearing impairment was determined using the first occurrences variables in UK Biobank (H90: conductive and sensorineural hearing loss, H91: other hearing loss) and a custom search of the inpatient and primary-care medical record. The codes used to identify hearing loss in the EHR and a more detailed procedure to derive the variable from all three sources were performed previously^35^.
- **TBI**: TBI was determined through a custom search of the inpatient and primary care medical records. The codes used are available in **Suppl. Table 4**.
- **Social isolation**: based on self-reported number of people in the household, frequency of family/friend visits, and selection of social leisure activities. Each of the three answers was scored for social isolation and the score thus ranged from zero to three. Participants with scores of zero or one were classified as not socially isolated as previously^36^. We took further steps to avoid misclassification in cases where at least one of the three questions was not answered by the participants. Participants with a score of zero were classified as not socially isolated only if at most one of the answers was missing. Participants with a score of one were classified as not socially isolated only if no answers were missing. If two or more answers were missing, the score was set to NA.
- **Depressed mood**: self-reported frequency of depressed mood in the week before the assessment; recorded as four categories: *not at all*, *several days*, *more than half the days*, and *nearly every day*.

**Suppl. Text 2**: some notes on the methodology.

*Algorithm for pseudoscale* construction

1. Create a list of drugs.^^[[5]](#footnote-5)^^
2. Draw *n*^^[[6]](#footnote-6)^^ drugs from the above list to set the size of the pseudoscale.
3. Score each drug by assigning it a potency score *s*.^^[[7]](#footnote-7)^^
4. Repeat steps (2) and (3) *x*-times to create *x* pseudoscales.

*Synthetic Minority Oversampling Technique (SMOTE).*

SMOTE changes the distribution of the outcome by creating synthetic observations for the minority class. We generated *X_syn_* synthetic observations of the minority class for each existing observation of the minority class such that:

$X_{syn}=floor(\frac{\frac{n(A_{0})}{n(A_{1})}}{3})$ ,

where *floor* is a function that rounds down to the closest whole number, n(A_0_) is the size of the majority group (outcome=0), and n(A_1_) is the size of the minority group (outcome=1). For example, in a sample in which 3,000 individuals experienced the outcome and 157,000 individuals did not, X_syn_=17. Thus, 3,000*17=51,000 synthetic observations would be generated.

*Time-to-event modelling*

The survival curves were estimated using the Kaplan-Meier estimator. Each Kaplan-Meier curve was constructed as follows. First, for each burden scale, its burden score was dichotomised based on quartiles, with the lowest quartile labelled ad “low” and the highest quartile labelled as “high”; intermediate values (between the first and the third quartile) were removed. Second, for the adjusted models, we calculated inverse probability of treatment weights (IPTWs), where the propensity score was calculated using logistic regression and matching was performed using generalised full matching^37,38^ via the *MatchIt* package^39^ in R. Third, we performed unadjusted Cox proportional hazards regression and weighted the resulting survival curves with the IPTWs calculated in the previous step to yield adjusted survival curves. Fourth, across the results for the 1000 models within a pseudoscales set (e.g., across-sampling of anticholinergic polypharmacy), we used Rubin’s rules after complementary log-log transformation to pool the 1000 Kaplan-Meier curves. Fifth, we applied smoothing on the Kaplan-Meier curves using local polynomial regression fitting. Effects accounting for the competing risk of death were estimated as subdistribution hazards using the approach by Fine and Gray^40^.

**Suppl. Table 5**: numbers (and % of total sample size) of missing observations. There were differences between the sample sizes for the initial samples for the different outcomes due to the differential removal of cases before time zero (**Suppl. Figure 1**). The table below shows the numbers (and %) for each outcome separately.

|  | **N (%) missing** | | |
| --- | --- | --- | --- |
|  | **Death** | **Dementia** | **Delirium** |
| Age | 0 | 0 | 0 |
| Sex | 0 | 0 | 0 |
| Data provider | 0 | 0 | 0 |
| Graduate degree | 2,531 (1.27) | 2,510 (1.26) | 2,526 (1.27) |
| Deprivation | 285 (0.14) | 285 (0.14) | 285 (0.14) |
| Alcohol consumption | 483 (0.24) | 482 (0.24) | 483 (0.24) |
| Waist circumference | 765 (0.38) | 760 (0.38) | 762 (0.38) |
| Smoking status | 1,025 (0.51) | 1,020 (0.51) | 1,025 (0.51) |
| Physical activity | 1,320 (0.66) | 1308 (0.66) | 1,312 (0.66) |
| Cerebrovascular disease | 0 | 0 | 0 |
| Lower respiratory disease | 0 |  |  |
| Diabetes | 0 | 0 |  |
| Liver disease | 0 |  |  |
| Influenza/pneumonia | 0 |  |  |
| Ischaemic heart disease | 0 |  |  |
| Colon cancer | 0 |  |  |
| Ovarian or prostate cancer | 0 |  |  |
| Lung cancer | 0 |  |  |
| Breast cancer | 0 |  |  |
| General cognitive ability |  | 288 (0.14) | 288 (0.14) |
| Air pollution* |  |  |  |
| *nitrogen dioxide* |  | 2,873 (1.44) |  |
| *nitrogen oxides* |  | 2,873 (1.44) |  |
| *pm_25_* |  | 24,647 (12.4) |  |
| Hypertension |  | 0 |  |
| Hypercholesterolemia |  | 0 |  |
| Depressed mood |  | 9,323 (4.7) |  |
| Social isolation |  | 531 (0.27) | 530 (0.27) |
| Loneliness |  | 3,711 (1.87) | 3,712 (1.86) |
| Mood disorder |  | 0 | 0 |
| CNS disorder |  |  |  |
| *inflammatory* |  | 0 |  |
| *atrophic* |  | 0 |  |
| *movement* |  | 0 |  |
| *demyelinating* |  | 0 |  |
| *paroxysmal* |  | 0 |  |
| *CNS cancer* |  | 0 |  |
| *TBI* |  | 0 |  |
| *other* |  | 0 |  |
| Hearing impairment |  | 0 | 0 |
| Psychotic disorder |  |  | 0 |
| Visual impairment |  |  | 0 |
| Sleep disorder |  |  | 0 |
| Endocrinopathy |  |  | 0 |
| Nutritional deficiency |  |  | 0 |
| Metabolic disorder |  |  | 0 |

**Note: air pollution data were systematically missing for participants from Scotland; see* ***Suppl. Text 1****.*

**Suppl. Table 6**: descriptive statistics (number and percentages, or median and interquartile range) for covariates used in our models. **Top**: table with descriptive statistics for outcome-specific covariates not depicted in the main text that were used in models for the 2015 prescription period. See main text for descriptive statistics of covariates used in all models for the 2015 prescriptions period. **Bottom** (see next two pages): table with descriptive statistics for all covariates for the 2004-2006 prescription period.
Q1-Q3: interquartile range; CNS: central nervous system; TBI: traumatic brain injury.

| ***Prescription period 2015*** | **N (%)** | | | | | |
| --- | --- | --- | --- | --- | --- | --- |
|  | **Death** | | **Dementia** | | **Delirium** | |
|  | **No (n = 182,538)** | **Yes (n = 11,968)** | **No (n = 157,756)** | **Yes (n = 2,563)** | **No (187,909)** | **Yes (n = 3,102)** |
| Lower respiratory disease | 31,215 (17.1) | 3,214 (26.9) |  |  |  |  |
| Diabetes | 11,809 (6.5) | 2,078 (17.4) | 10,657 (6.8) | 463 (18.1) |  |  |
| Liver disease | 4,892 (2.7) | 726 (6.1) |  |  |  |  |
| Influenza/pneumonia | 15,388 (8.4) | 1,826 (15.3) |  |  |  |  |
| Ischaemic heart disease | 14,033 (7.7) | 2,561 (21.4) |  |  |  |  |
| Colon cancer | 1,677 (0.92) | 362 (3.0) |  |  |  |  |
| Ovarian or prostate cancer | 5,452 (3.0) | 1,005(8.4) |  |  |  |  |
| Lung cancer | 204 (0.11) | 242 (2.0) |  |  |  |  |
| Breast cancer | 5,137 (2.8) | 633 (5.3) |  |  |  |  |
| General cognitive ability, median (Q1-Q3) |  |  | 0.05 (-0.45-0.54) | -0.27 (-0.78-0.20) | 0.04 (-0.44-0.52) | -0.23 (-0.75-0.24) |
| *Air pollution, median (Q1-Q3) |  |  |  |  |  |  |
| *nitrogen dioxide (μg/m^3^)* |  |  | 25.5 (21-30.2) | 25.8 (21.6-30.3) |  |  |
| *nitrogen oxides (μg/m^3^)* |  |  | 41.5 (33.7-49.4) | 41.9 (34.7-49.8) |  |  |
| *pm_25_ (μg/m^3^)* |  |  | 9.9 (9.3-10.5) | 10.0 (9.3-10.6) |  |  |
| Hypertension |  |  | 51,287 (32.5) | 1,422 (55.5) |  |  |
| Hypercholesterolemia |  |  | 33,553 (21.3) | 1,039 (40.5) |  |  |
| Depressed mood |  |  |  |  |  |  |
| *not at all* |  |  | 121,324 (76.9) | 1,992 (77.7) |  |  |
| *several days* |  |  | 28,634 (18.2) | 421 (16.4) |  |  |
| *more than half the days* |  |  | 4,779 (3.0) | 91 (3.5) |  |  |
| *nearly every day* |  |  | 3,019 (1.9) | 60 (2.3) |  |  |
| Social isolation |  |  | 72,757 (46.1) | 1,300 (50.7) | 87,109 (46.4) | 1,654 (53.3) |
| Loneliness |  |  | 27,883 (17.7) | 460 (17.9) | 34,202 (18.2) | 680 (21.9) |
| Mood disorder |  |  | 21,324 (13.5) | 449 (17.5) | 25,415 (13.5) | 647 (20.9) |
| CNS disorder |  |  |  |  |  |  |
| *inflammatory* |  |  | 1,168 (0.74) | 24 (0.94) |  |  |
| *atrophic* |  |  | 211 (0.13) | 13 (0.51) |  |  |
| *movement* |  |  | 3,534 (2.2) | 226 (8.8) |  |  |
| *demyelinating* |  |  | 811 (0.51) | 21 (0.82) |  |  |
| *paroxysmal* |  |  | 5,167 (3.3) | 238 (9.3) |  |  |
| *CNS cancer* |  |  | 302 (0.19) | 13 (0.51) |  |  |
| *TBI* |  |  | 4,956 (3.1) | 130 (5.1) |  |  |
| *other* |  |  | 2,138 (1.4) | 89 (3.4) |  |  |
| Hearing impairment |  |  | 53,792 (34.1) | 1,115 (43.5) | 62,504 (33.3) | 1,328 (42.8) |
| Psychotic disorder |  |  |  |  | 698 (0.37) | 54 (1.7) |
| Visual impairment |  |  |  |  | 1,302 (0.69) | 57 (1.8) |
| Sleep disorder |  |  |  |  | 9,462 (5.0) | 264 (8.5) |
| Endocrinopathy |  |  |  |  | 20,698 (11.0) | 505 (16.3) |
| Nutritional deficiency |  |  |  |  | 2,538 (1.4) | 99 (3.2) |
| Metabolic disorder |  |  |  |  | 44,010 (23.4) | 1,478 (47.6) |

**Note: for pollution, the statistics of the components that were used to calculate the principal component are shown*.

| ***Prescription period 2004-2006*** | **N (%)** | | | | | |
| --- | --- | --- | --- | --- | --- | --- |
| Outcome | **Death** | | **Dementia** | | **Delirium** | |
|  | **No (n = 146,306)** | **Yes  (n = 13,442)** | **No (n = 145,362)** | **Yes (n = 2,709)** | **No (n = 154,401)** | **Yes (n = 2,762)** |
| Follow-up (years), median (Q1-Q3) | 15.8 (15.8-15.8) | 11.7 (8.1-14.0) | 15.8 (15.8-15.8) | 12.6 (10.4-14.4) | 15.8 (15.8-15.8) | 13.1 (11.3-14.6) |
| Age (years), median (Q1-Q3) | 55.9 (48.4-61.1) | 61.7 (54.4-64.9) | 56.4 (48.9-61.5) | 63.8 (60.7-66.2) | 56.4 (48.9-61.5) | 63.1 (59.7-65.7) |
| Female sex | 82,327 (56.3) | 5,442 (40.5) | 79,707 (54.8) | 1,271 (46.9) | 69,294 (44.9) | 1,596 (57.8) |
| Data provider |  |  |  |  |  |  |
| *Missing* | 1,575 (1.1) | 341 (2.5) | 477 (0.32) | 3 (0.11) | 1,869 (1.2) | 22 (0.80) |
| *England (Vision)* | 13,954 (9.5) | 774 (5.8) | 13,570 (9.3) | 250 (9.2) | 14,150 (9.2) | 336 (12.2) |
| *England (TPP)* | 114,980 (78.6) | 10,741 (79.9) | 115,384 (79.4) | 2188 (80.8) | 121,444 (78.7) | 2,249 (81.4) |
| *Wales* | 15,797 (10.8) | 1,589 (11.8) | 15,931 (11.0) | 268 (9.9) | 16,938 (11.0) | 155 (5.6) |
| *Scotland** |  |  |  |  |  |  |
| Graduate degree | 46,363 (31.7) | 2,989 (22.2) | 45,682 (31.4) | 522 (19.3) | 48,128 (31.2) | 542 (19.6) |
| Deprivation, median (Q1-Q3) | -2.3 (-3.7-0.03) | -1.9 (-3.5-0.98) | -2.4 (-3.7--0.04) | -2.2 (-3.6-0.55) | -2.3 (-3.7-0.02) | -1.9 (-3.5-0.97) |
| Alcohol consumption |  |  |  |  |  |  |
| *Daily or almost daily* | 29,133 (19.9) | 3,122 (23.2) | 29,669 (20.4) | 556 (20.5) | 31,260 (20.2) | 602 (21.8) |
| *Three or four times a week* | 34,806 (23.8) | 2,664 (19.8) | 34,591 (23.8) | 517 (19.1) | 36,430 (23.6) | 530 (19.2) |
| *Once or twice a week* | 39,033 (26.7) | 3,158 (23.5) | 38,446 (26.4) | 674 (24.9) | 40,907 (26.5) | 611 (22.1) |
| *One to three times a month* | 16,622 (11.4) | 1,315 (9.8) | 16,309 (11.2) | 259 (9.6) | 17,354 (11.2) | 270 (9.8) |
| *Special occasions* | 16,036 (11.0) | 1,704 (12.7) | 15,753 (10.8) | 374 (13.8) | 16,970 (11.0) | 383 (13.9) |
| *Never* | 10,676 (7.3) | 1,479 (11.0) | 10,594 (7.3) | 329 (12.1) | 11,480 (7.4) | 366 (13.3) |
| Waist circumference (cm), median (Q1-Q3) | 89 (80-98) | 95 (86-104) | 90 (81-99) | 92 (83-102) | 90 (81-99) | 95 (86-105) |
| Smoking status |  |  |  |  |  |  |
| *Never smoker* | 83,037 (56.8) | 5,192 (38.6) | 80,465 (55.4) | 1,288 (47.5) | 85,579 (55.4) | 1,162 (42.1) |
| *Former smoker* | 49,874 (34.1) | 5,866 (43.6) | 50,774 (34.9) | 1,185 (43.7) | 53,728 (34.8) | 1,236 (44.8) |
| *Current smoker* | 13,395 (9.2) | 2,384 (17.7) | 14,123 (9.7) | 236 (8.7) | 15,094 (9.8) | 364 (13.2) |
| Physical activity |  |  |  |  |  |  |
| *None* | 8,403 (5.7) | 1,575 (11.7) | 8,515 (5.9) | 267 (9.9) | 9,365 (6.1) | 321 (11.6) |
| *Light* | 5,382 (3.7) | 794 (5.9) | 5,514 (3.8) | 147 (5.4) | 5,898 (3.8) | 165 (6.0) |
| *Moderate* | 117,363 (80.2) | 10,402 (77.4) | 116,482 (80.1) | 2,184 (80.6) | 123,587 (80.0) | 2,157 (78.1) |
| *Strenuous* | 15,158 (10.4) | 671 (5.0) | 14,851 (10.2) | 111 (4.1) | 15,551 (10.1) | 119 (4.3) |
| Cerebrovascular disease | 1,769 (1.2) | 558 (4.2) | 1,989 (1.4) | 121 (4.5) | 2,146 (1.4) | 127 (4.6) |

**Note: participants from Scotland were excluded from analyses of all three outcomes due to a system-wide block of prescription records before the year 2012 (see also Suppl. Text 1 and UK Biobank^26^)*

| ***Prescription period 2004-2006*** | **N (%)** | | | | | |
| --- | --- | --- | --- | --- | --- | --- |
|  | **Death** | | **Dementia** | | **Delirium** | |
|  | **No (n = 146,306)** | **Yes  (n = 13,442)** | **No (n = 145,362)** | **Yes (n = 2,709)** | **No (n = 154,401)** | **Yes (n = 2,762)** |
| Lower respiratory disease | 20,252 (13.8) | 2,453 (18.2) |  |  |  |  |
| Diabetes | 4,879 (3.3) | 1,452 (10.8) | 5,494 (3.8) | 300 (11.1) |  |  |
| Liver disease | 1,301 (0.89) | 295 (2.2) |  |  |  |  |
| Influenza/pneumonia | 8,750 (6.0) | 1,030 (7.7) |  |  |  |  |
| Ischaemic heart disease | 5,563 (3.8) | 1,773 (13.2) |  |  |  |  |
| Colon cancer | 539 (0.37) | 141 (1.0) |  |  |  |  |
| Ovarian or prostate cancer | 4,355 (3.0) | 1,138 (8.5) |  |  |  |  |
| Lung cancer | 39 (0.03) | 76 (0.57) |  |  |  |  |
| Breast cancer | 2,302 (1.6) | 457 (3.4) |  |  |  |  |
| General cognitive ability, median (Q1-Q3) |  |  | 0.04 (-0.45-0.53) | -0.29 (-0.82-0.20) | 0.03 (-0.46-0.53) | -0.23 (-0.75-0.26) |
| *Air pollution, median (Q1-Q3) |  |  |  |  |  |  |
| *nitrogen dioxide (μg/m^3^)* |  |  | 25.3 (21.0-30.0) | 25.7 (21.4-30.1) |  |  |
| *nitrogen oxides (μg/m^3^)* |  |  | 41.4 (33.6-49.1) | 41.8 (34.6-49.6) |  |  |
| *pm_25_ (μg/m^3^)* |  |  | 9.9 (9.3-10.5) | 10.0 (9.3-10.5) |  |  |
| Hypertension |  |  | 31,951 (22.0) | 1,053 (38.9) |  |  |
| Hypercholesterolemia |  |  | 17,021 (11.7) | 680 (25.1) |  |  |
| Depressed mood |  |  |  |  |  |  |
| *not at all* |  |  | 112,209 (77.2) | 2,084 (76.9) |  |  |
| *several days* |  |  | 26,200 (18.0) | 455 (16.8) |  |  |
| *more than half the days* |  |  | 4,307 (3.0) | 106 (3.9) |  |  |
| *nearly every day* |  |  | 2,646 (1.8) | 64 (2.4) |  |  |
| Social isolation |  |  | 66,356 (45.6) | 1,373 (50.7) | 70,944 (45.9) | 1,458 (52.8) |
| Loneliness |  |  | 25,246 (17.4) | 498 (18.4) | 27,562 (17.9) | 613 (22.2) |
| Mood disorder |  |  | 14,430 (9.9) | 329 (12.1) | 15.405 (10.0) | 406 (14.7) |
| CNS disorder |  |  |  |  |  |  |
| *inflammatory* |  |  | 973 (0.67) | 23 (0.85) |  |  |
| *atrophic* |  |  | 102 (0.07) | 5 (0.18) |  |  |
| *movement* |  |  | 1,323 (0.91) | 99 (3.7) |  |  |
| *demyelinating* |  |  | 598 (0.41) | 20 (0.74) |  |  |
| *paroxysmal* |  |  | 2,717 (1.9) | 130 (4.8) |  |  |
| *CNS cancer* |  |  | 164 (0.11) | 7 (0.26) |  |  |
| *TBI* |  |  | 3,080 (2.1) | 78 (2.9) |  |  |
| *other* |  |  | 1,267 (0.87) | 134 (1.3) |  |  |
| Hearing impairment |  |  | 50,009 (34.4) | 1,160 (42.8) | 52,880 (34.2) | 1,244 (45.0) |
| Psychotic disorder |  |  |  |  | 388 (0.25) | 29 (1.0) |
| Visual impairment |  |  |  |  | 408 (0.26) | 12 (0.43) |
| Sleep disorder |  |  |  |  | 4,581 (3.0) | 126 (4.6) |
| Endocrinopathy |  |  |  |  | 11,691 (7.6) | 288 (10.4) |
| Nutritional deficiency |  |  |  |  | 356 (0.23) | 11 (0.40) |
| Metabolic disorder |  |  |  |  | 20,217 (13.1) | 789 (28.6) |

**Suppl. Table 7**: comparison between subset with primary-care data and subset without primary-care data. Descriptive statistics are shown for participants that were alive at the end of 2015, that by then had not yet been diagnosed with either dementia or delirium, and for whom complete data for covariates for all three outcomes were available. The second and third columns contain values for participants for whom primary-care data were available or unavailable, respectively. Thus, in the second column are most participants that were included in analytical samples for analyses in the present study. The variable *data provider* was not included in the table because it refers to the data provider in primary-care data and thus for all participants without primary-care data, this variable is missing.
Q1-Q3: interquartile range; CNS: central nervous system; TBI: traumatic brain injury.

|  | **Sample** | |
| --- | --- | --- |
|  | analytical GP (n=151,047) | non-GP (n=232,317) |
| Death | 9,102 (6.0) | 13,636 (5.9) |
| Dementia | 2,408 (1.6) | 3,572 (1.5) |
| Delirium | 2,389 (1.6) | 3,647 (1.6) |
| Age (years), median (Q1-Q3) | 65.2 (57.5-70.4) | 64.9 (57.2-70.4) |
| Female sex | 82,785 (54.8) | 126,121 (54.3) |
| Graduate degree | 48,493 (32.1) | 78,603 (33.8) |
| Deprivation, median (Q1-Q3) | -2.3 (-3.7-0.14) | -2.2 (-3.7-0.4) |
| Alcohol consumption |  |  |
| *Daily or almost daily* | 30,782 (20.4) | 49,433 (21.3) |
| *Three or four times a week* | 35,659 (23.6) | 54,856 (23.6) |
| *Once or twice a week* | 39,640 (26.2) | 59,180 (25.5) |
| *One to three times a month* | 16,917 (11.2) | 25,709 (11.1) |
| *Special occasions* | 16,617 (11.0) | 25,909 (11.2) |
| *Never* | 11,432 (7.6) | 17,230 (7.4) |
| Waist circumference (cm), median (Q1-Q3) | 90 (81-99) | 90 (80-99) |
| Smoking status |  |  |
| *Never smoker* | 83,599 (55.3) | 128,323 (55.2) |
| *Former smoker* | 52,567 (34.8) | 80,873 (34.8) |
| *Current smoker* | 14,881 (9.9) | 23,121 (10.0) |
| Physical activity |  |  |
| *None* | 8,930 (5.9) | 13,466 (5.8) |
| *Light* | 5,691 (3.8) | 8,455 (3.6) |
| *Moderate* | 120,919 (80.1) | 185,422 (79.8) |
| *Strenuous* | 15,507 (10.3) | 24,974 (10.7) |
| Cerebrovascular disease | 4,469 (3.0) | 5,766 (2.5) |
| Lower respiratory disease | 27,338 (18.1) | 35,619 (15.3) |
| Diabetes | 10,603 (7.0) | 15,096 (6.5) |
| Liver disease | 4,461 (3.0) | 3,088 (1.3) |
| Influenza/pneumonia | 13,828 (9.2) | 8,071 (3.5) |
| Ischaemic heart disease | 12,670 (8.4) | 17,911 (7.2) |
| Colon cancer | 1,567 (1.0) | 2,347 (1.0) |
| Ovarian or prostate cancer | 5,096 (3.4) | 8,087 (3.5) |
| Lung cancer | 336 (0.22) | 534 (0.23) |
| Breast cancer | 4,486 (3.0) | 7,006 (3.0) |
| General cognitive ability, median (Q1-Q3) | 0.03 (-0.41-0.48) | 0.04 (-0.39-0.47) |
| Air pollution, median (Q1-Q3) |  |  |
| *nitrogen dioxide (μg/m^3^)* | 25.5 (21.0-30.2) | 26.3 (21.4-31.7) |
| *nitrogen oxides (μg/m^3^)* | 41.5 (33.7-49.4) | 42.3 (34.1-51.2) |
| *pm_25_ (μg/m^3^)* | 9.9 (9.3-10.5) | 9.9 (9.3-10.6) |
| Hypertension | 49,999 (33.1) | 72,666 (31.3) |
| Hypercholesterolemia | 33,137 (21.9) | 42,979 (18.5) |
| Depressed mood |  |  |
| *not at all* | 116,046 (76.8) | 178,995 (77.0) |
| *several days* | 27,449 (18.2) | 42,473 (18.3) |
| *more than half the days* | 4,621 (3.1) | 6,694 (2.9) |
| *nearly every day* | 2,931 (1.9) | 4,155 (1.8) |
| Social isolation | 69,881 (46.3) | 108,284 (46.6) |
| Loneliness | 26,826 (17.8) | 40,643 (17.5) |
| Mood disorder | 20,898 (13.8) | 18,842 (8.1) |
| CNS disorder |  |  |
| *inflammatory* | 1,124 (0.74) | 1,539 (0.66) |
| *atrophic* | 207 (0.14) | 145 (0.06) |
| *movement* | 3,567 (2.4) | 1,232 (0.53) |
| *demyelinating* | 782 (0.52) | 939 (0.40) |
| *paroxysmal* | 5,093 (3.4) | 4,755 (2.1) |
| *CNS cancer* | 299 (0.20) | 482 (0.21) |
| *TBI* | 4,806 (3.2) | 1,643 (0.71) |
| *other* | 2,108 (1.4) | 2,026 (0.87) |
| Hearing impairment | 53,018 (35.1) | 69,172 (29.8) |
| Psychotic disorder | 560 (0.37) | 673 (0.29) |
| Visual impairment | 1,141 (0.76) | 479 (0.21) |
| Sleep disorder | 8,024 (5.3) | 3,704 (1.6) |
| Endocrinopathy | 16,897 (11.2) | 18,994 (8.2) |
| Nutritional deficiency | 2,036 (1.3) | 1,007 (0.43) |
| Metabolic disorder | 37,247 (24.7) | 46,166 (19.9) |

**Suppl. Figure 3**: histograms of effect sizes for general (**blue**) and anticholinergic (**red**) within-sampling pseudoscales when estimating the effects of drug burden on death, dementia, or delirium. The dashed line represents the effect size for ABS to which the distributions of effect sizes for the pseudoscales correspond (in terms of scale size and distribution of potency scores); the shaded rectangle represents the 95% CI for the effect estimate of the association between the score according to the ABS and the outcome.

*Death*


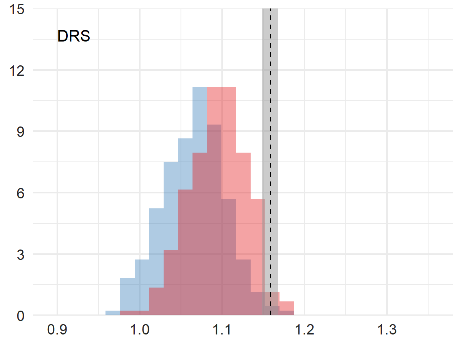

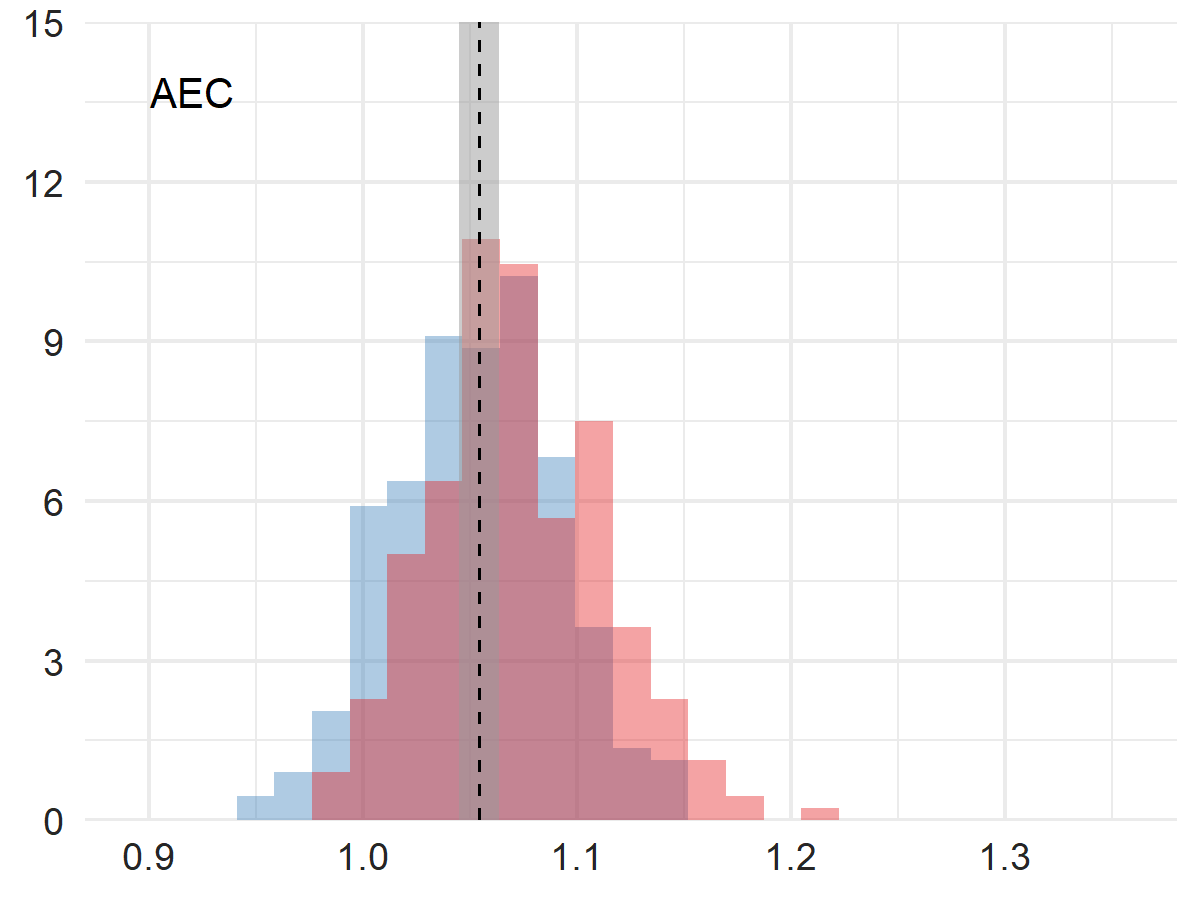

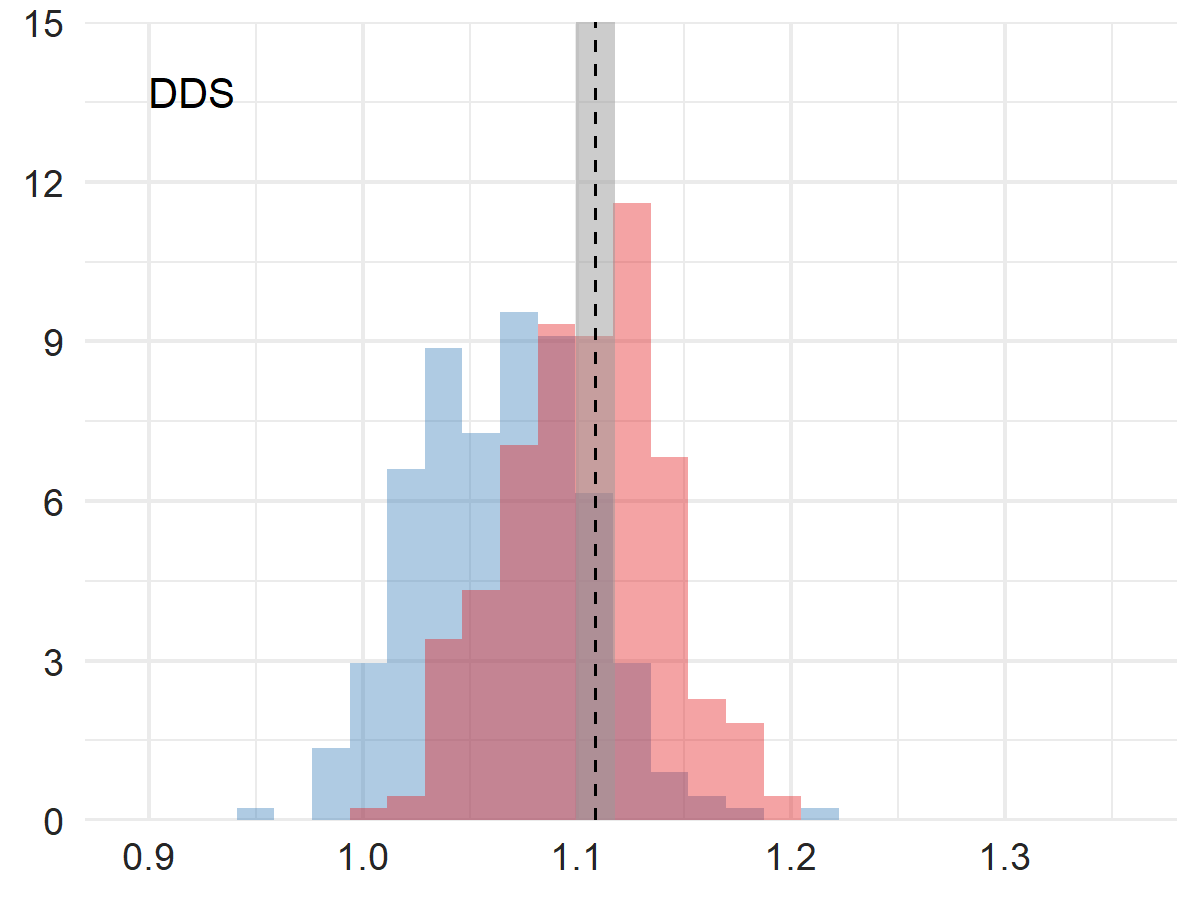

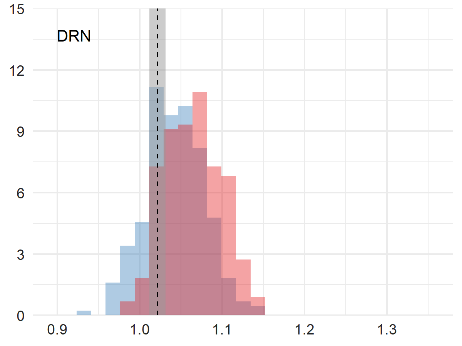

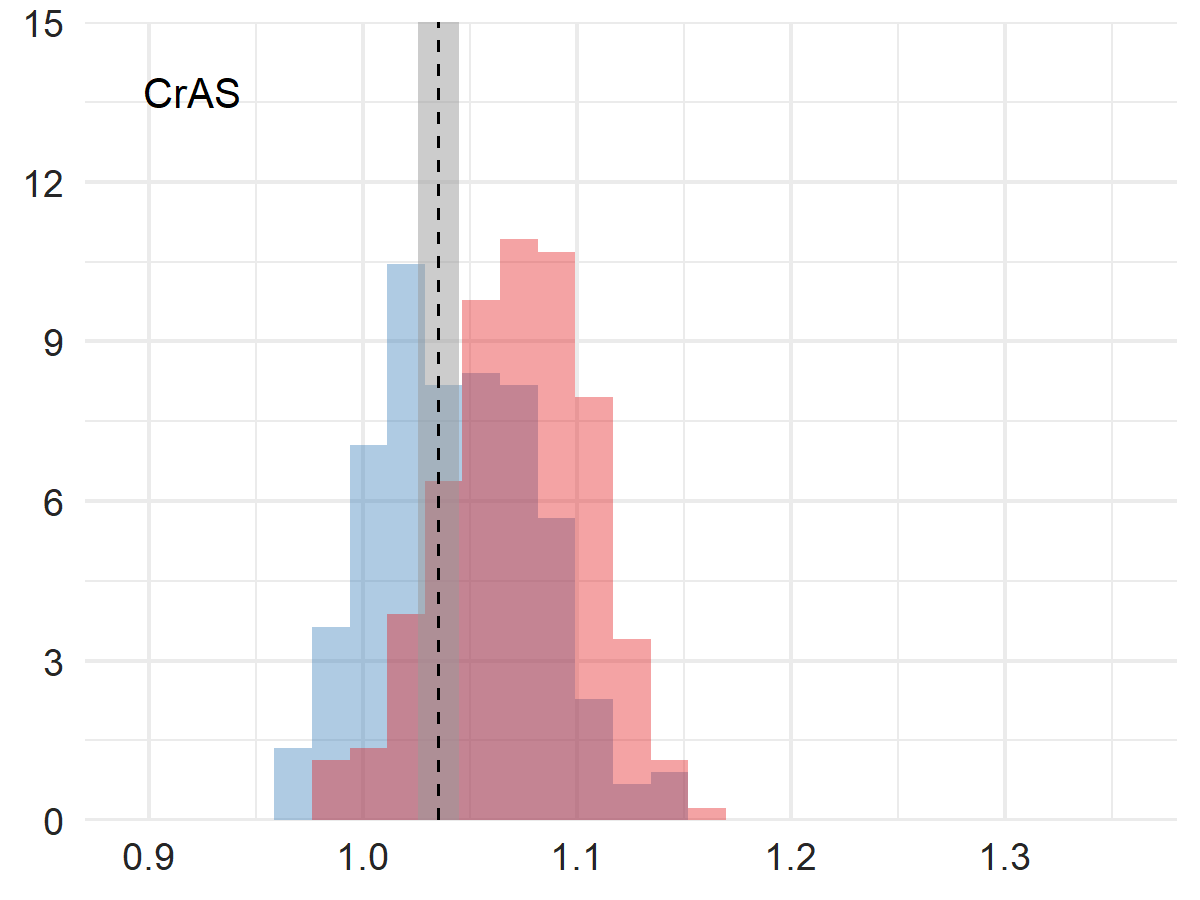

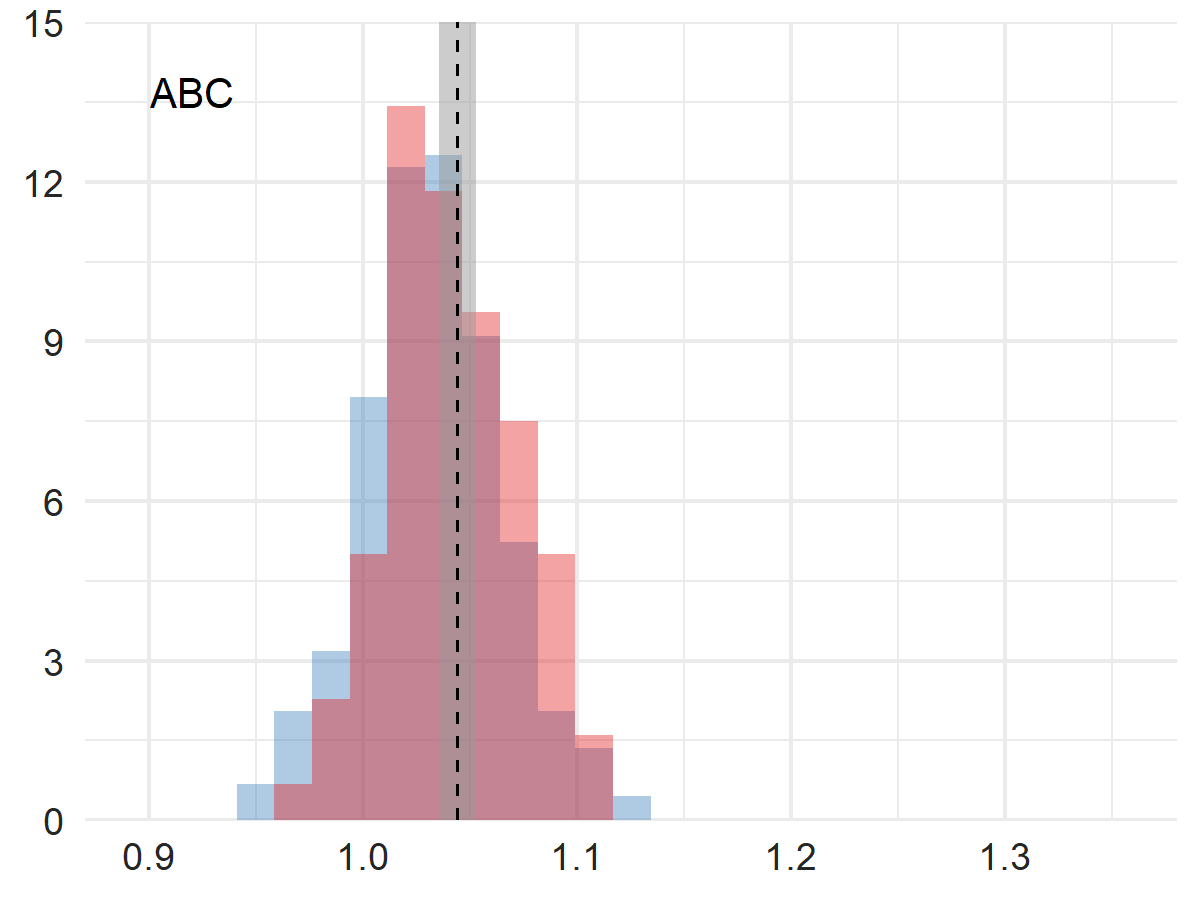

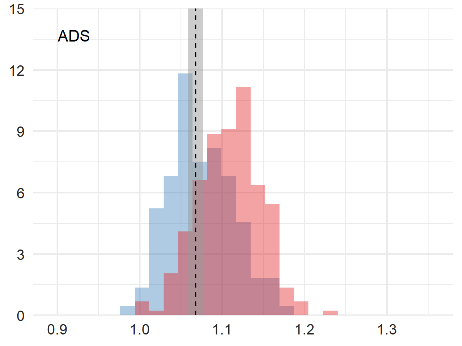

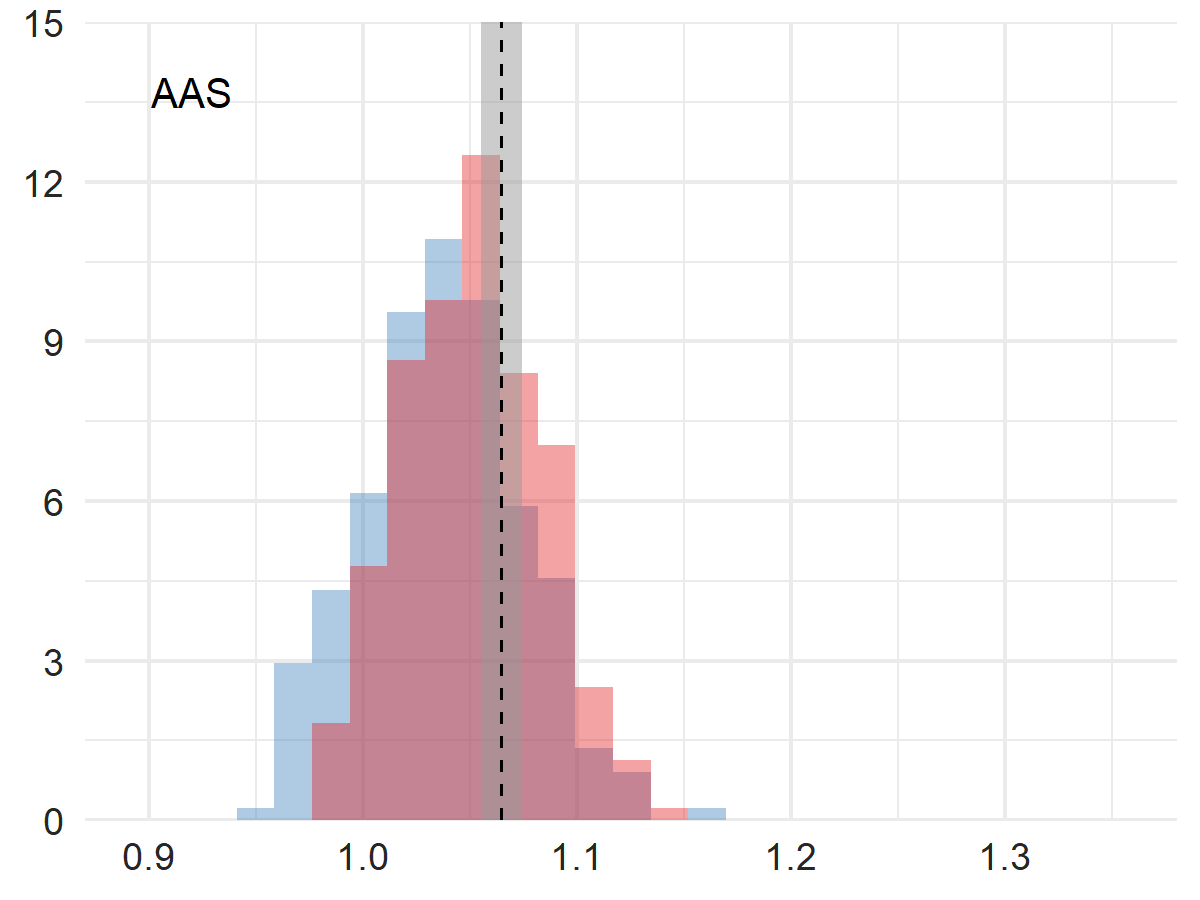

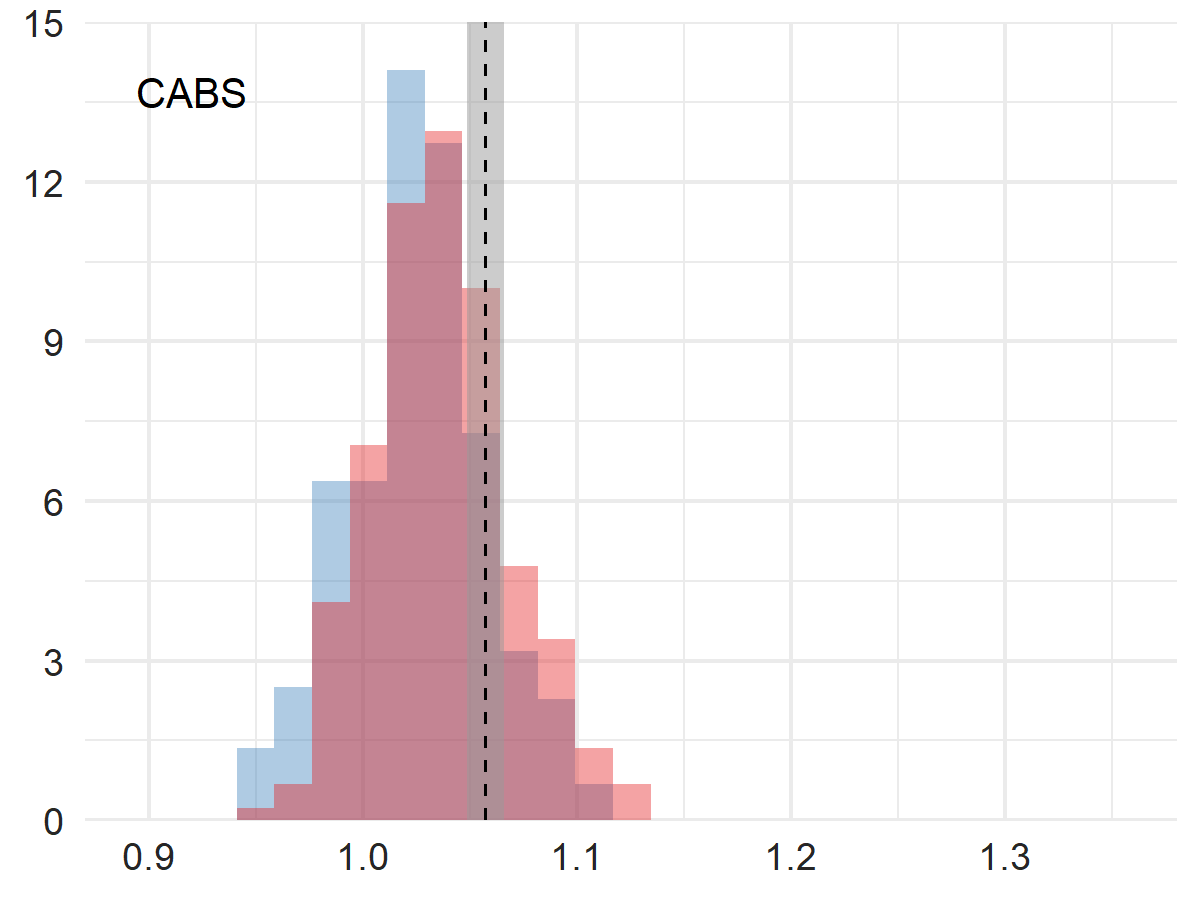

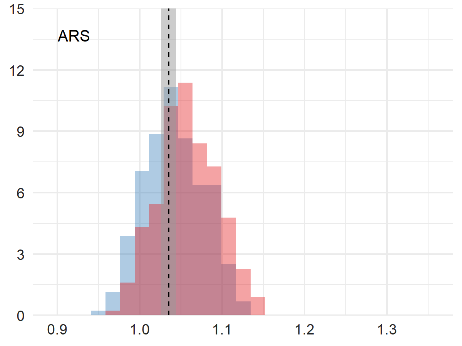

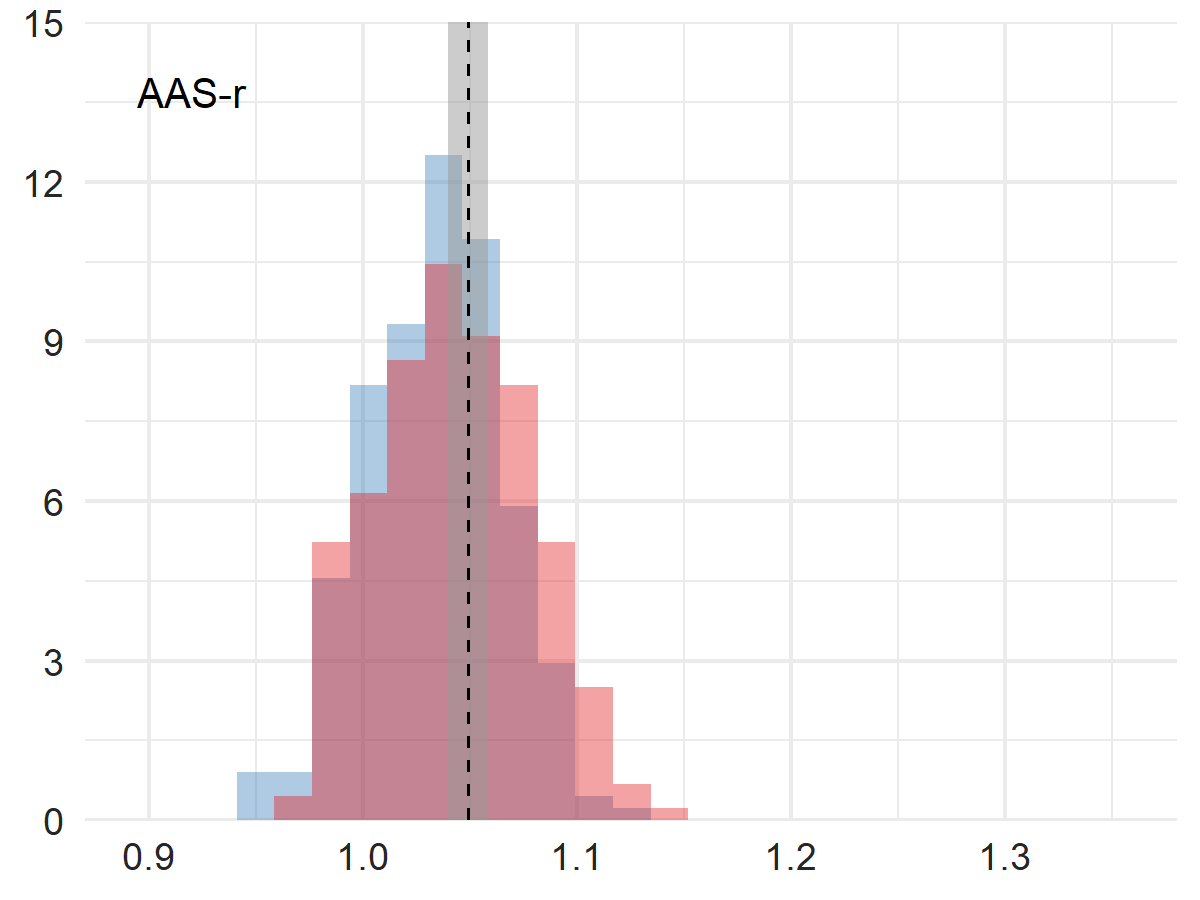

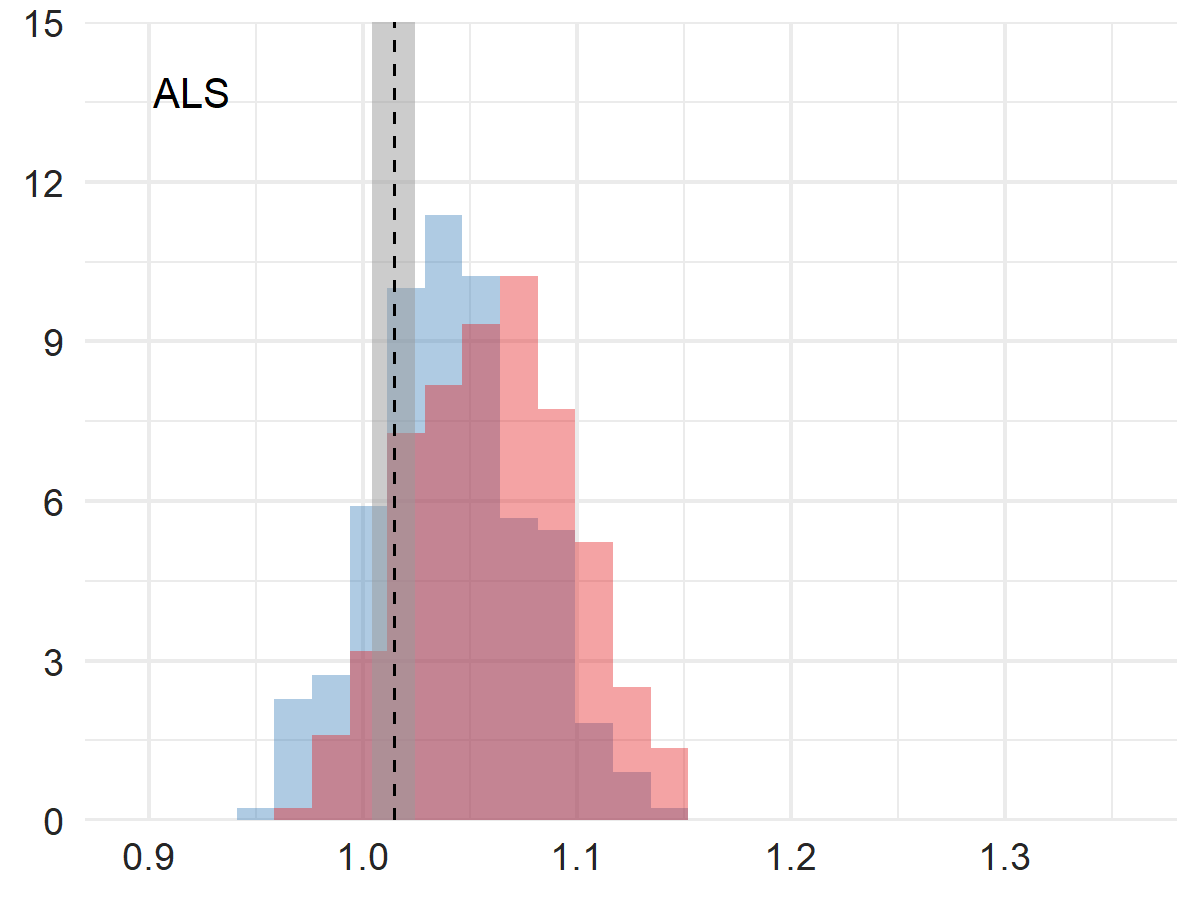

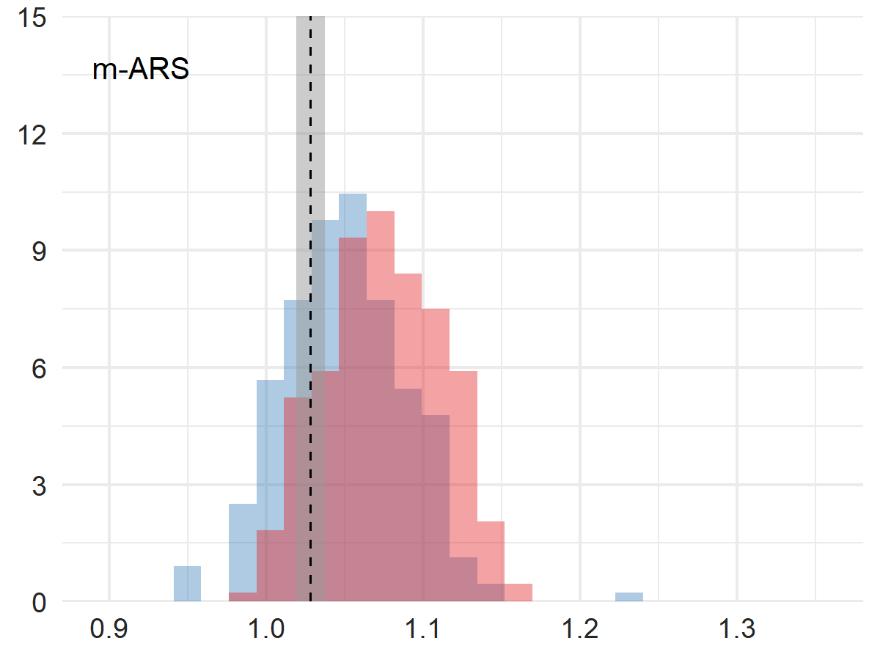

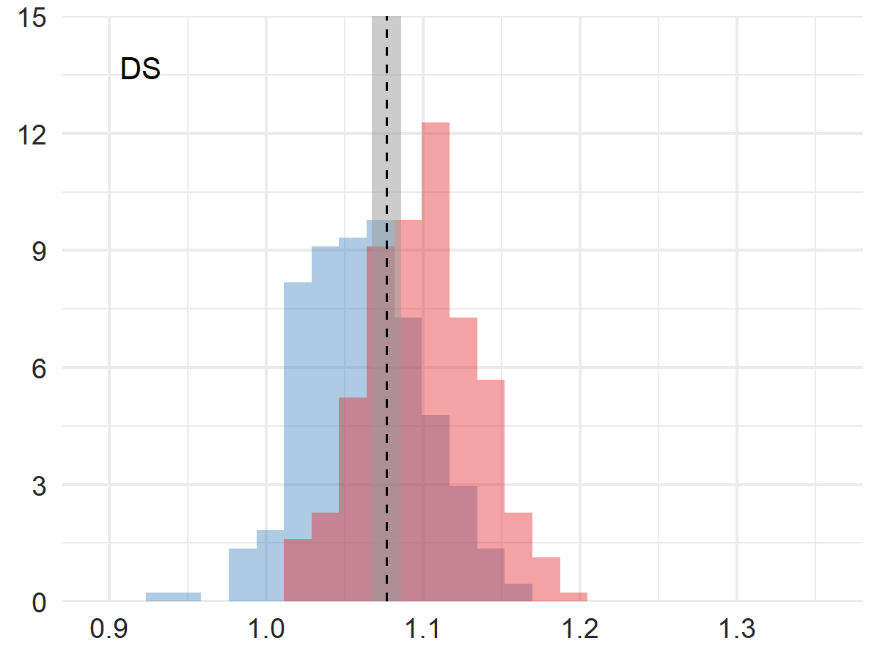

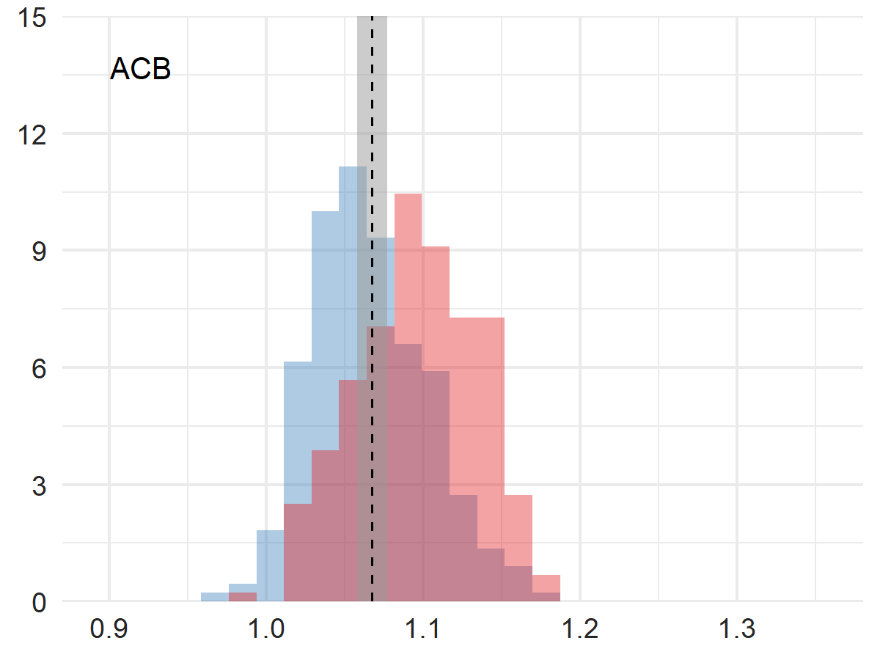

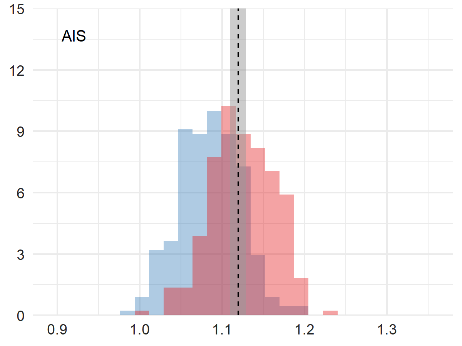

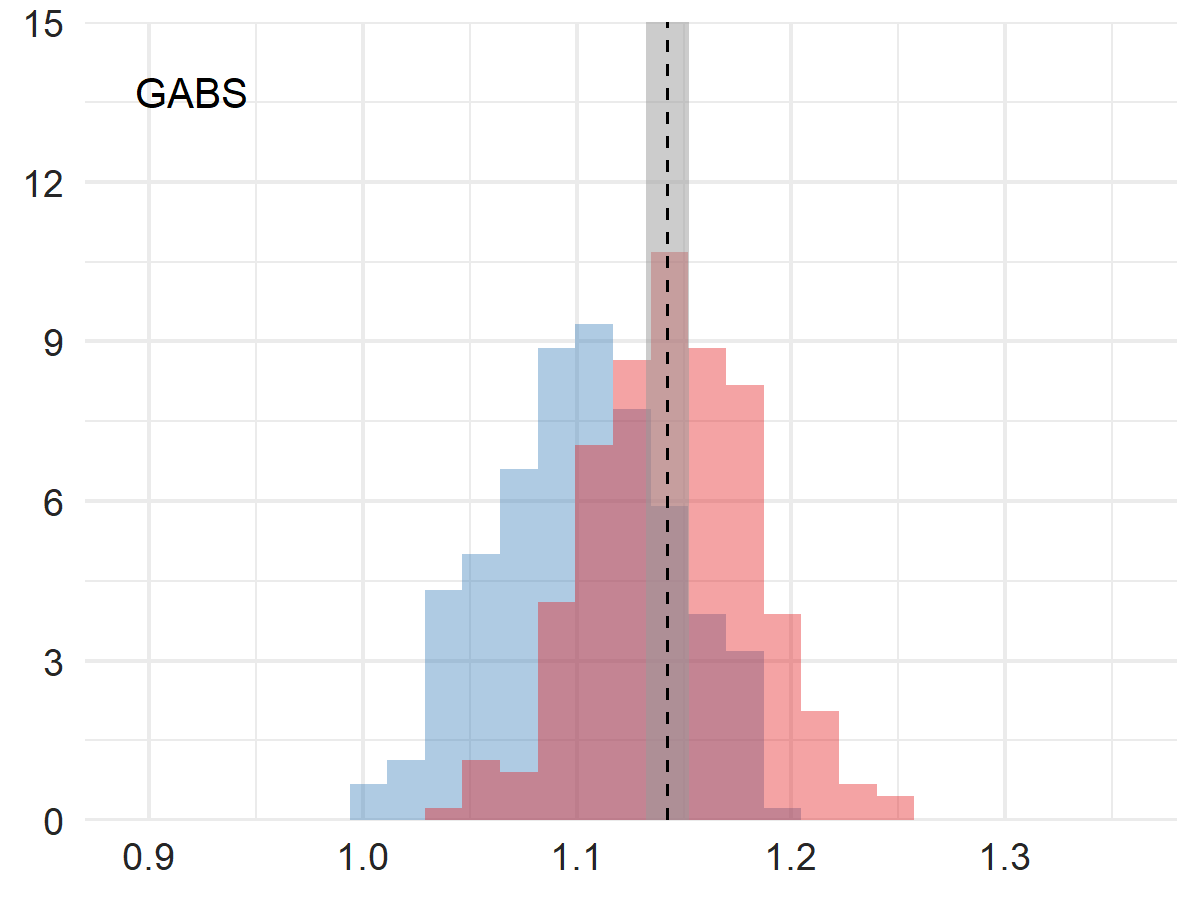

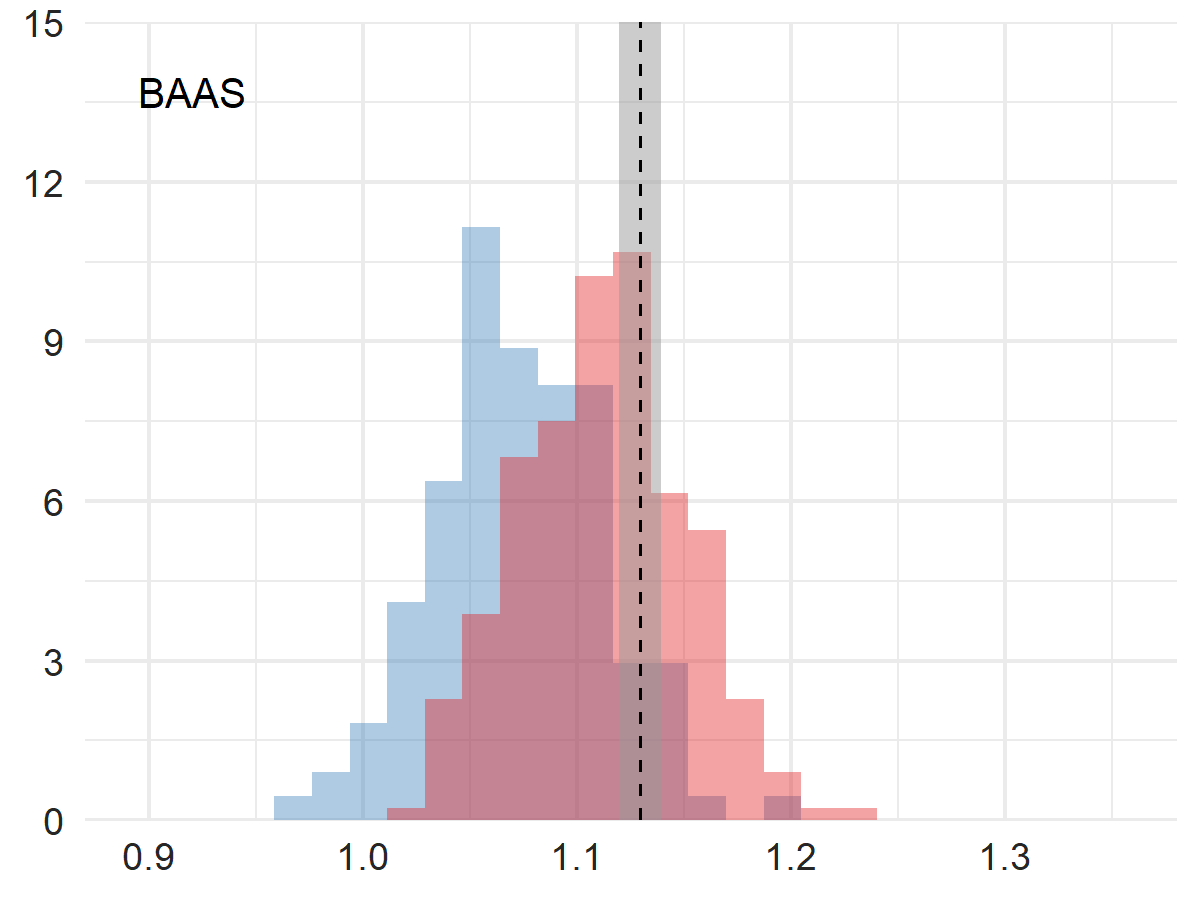

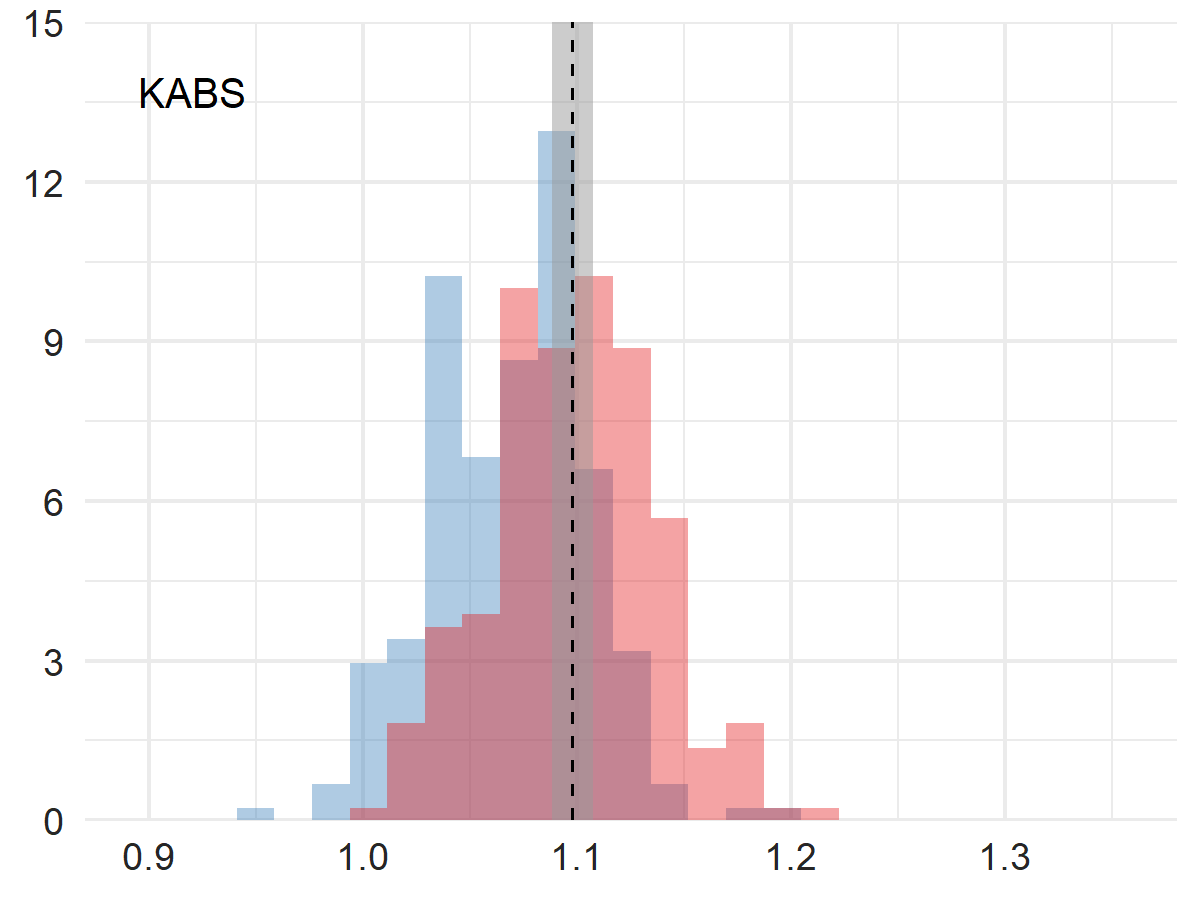

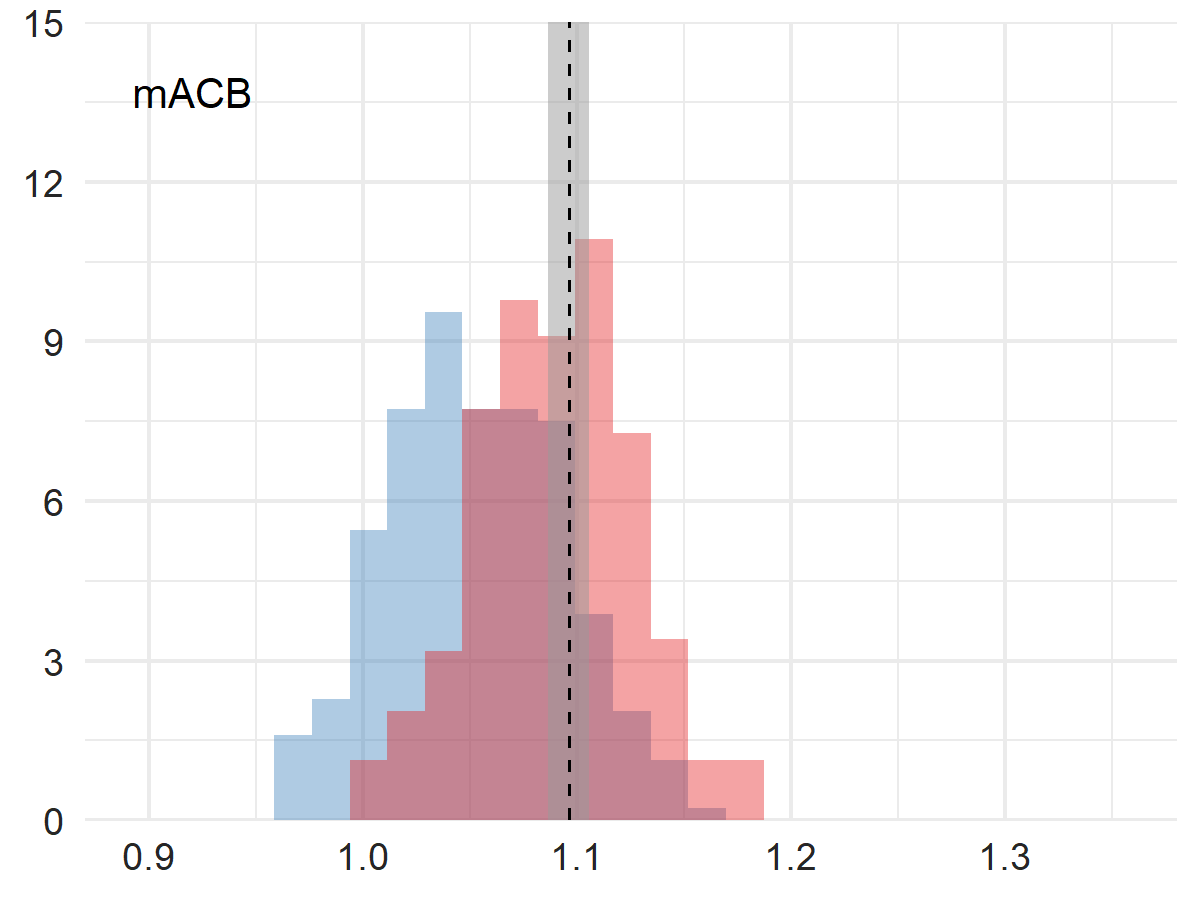

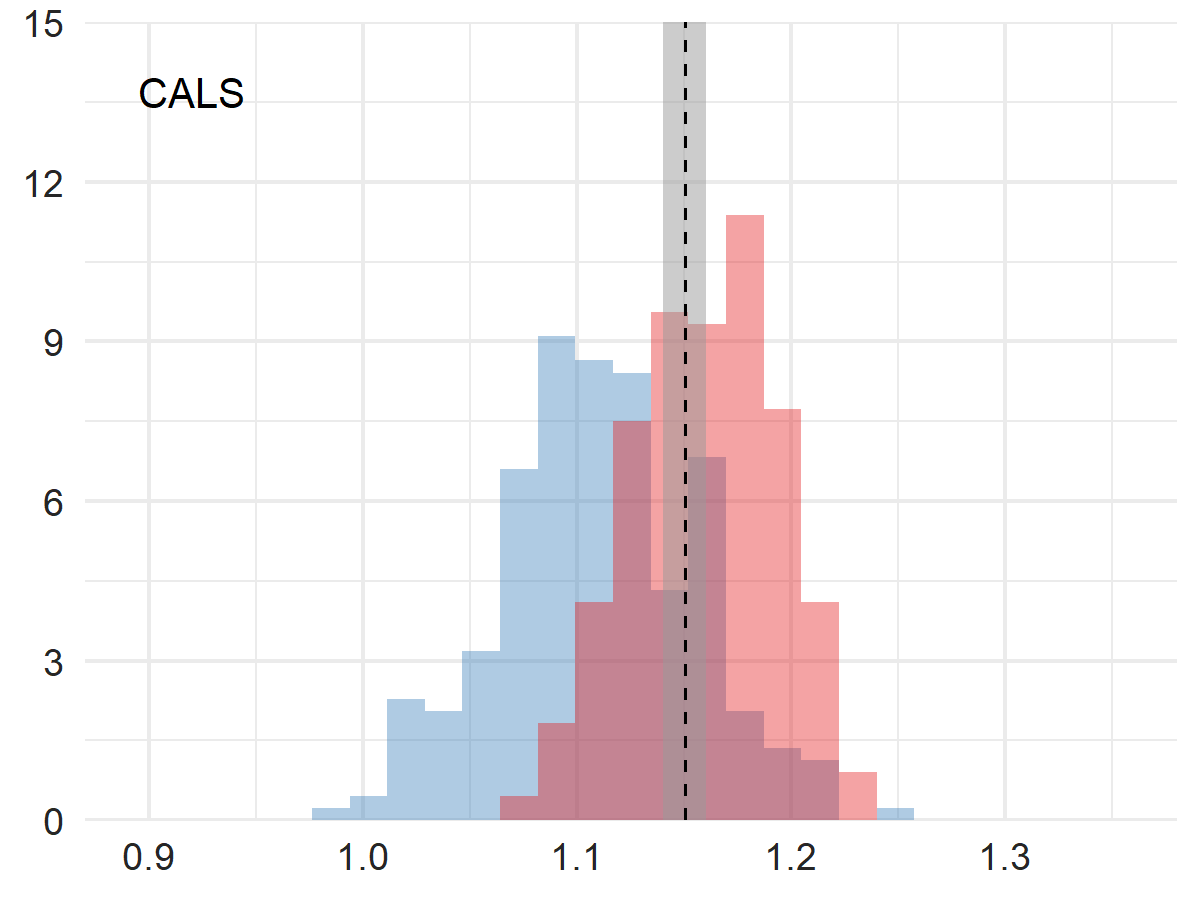

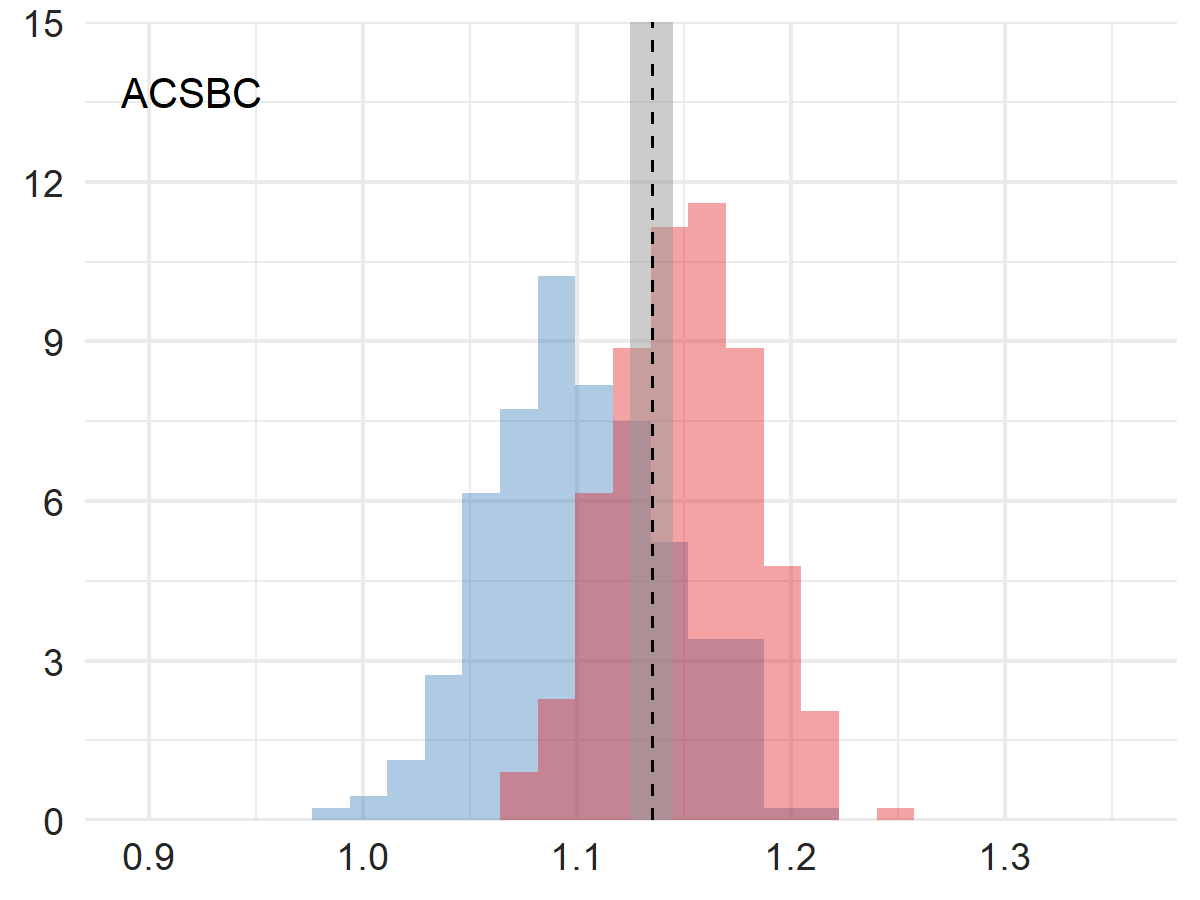

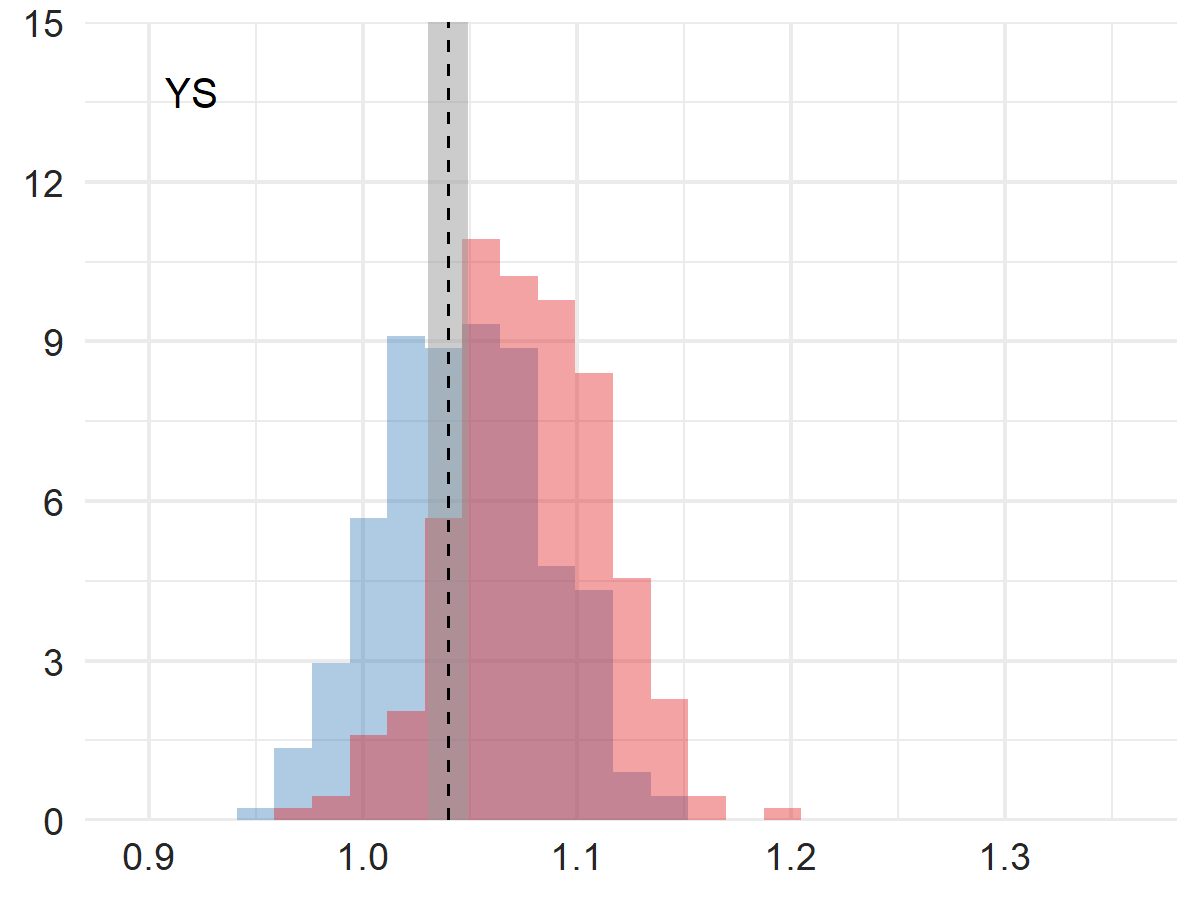


*Dementia*


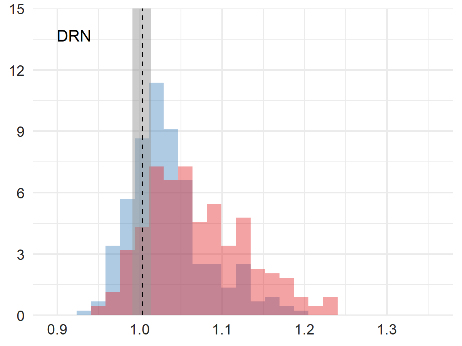

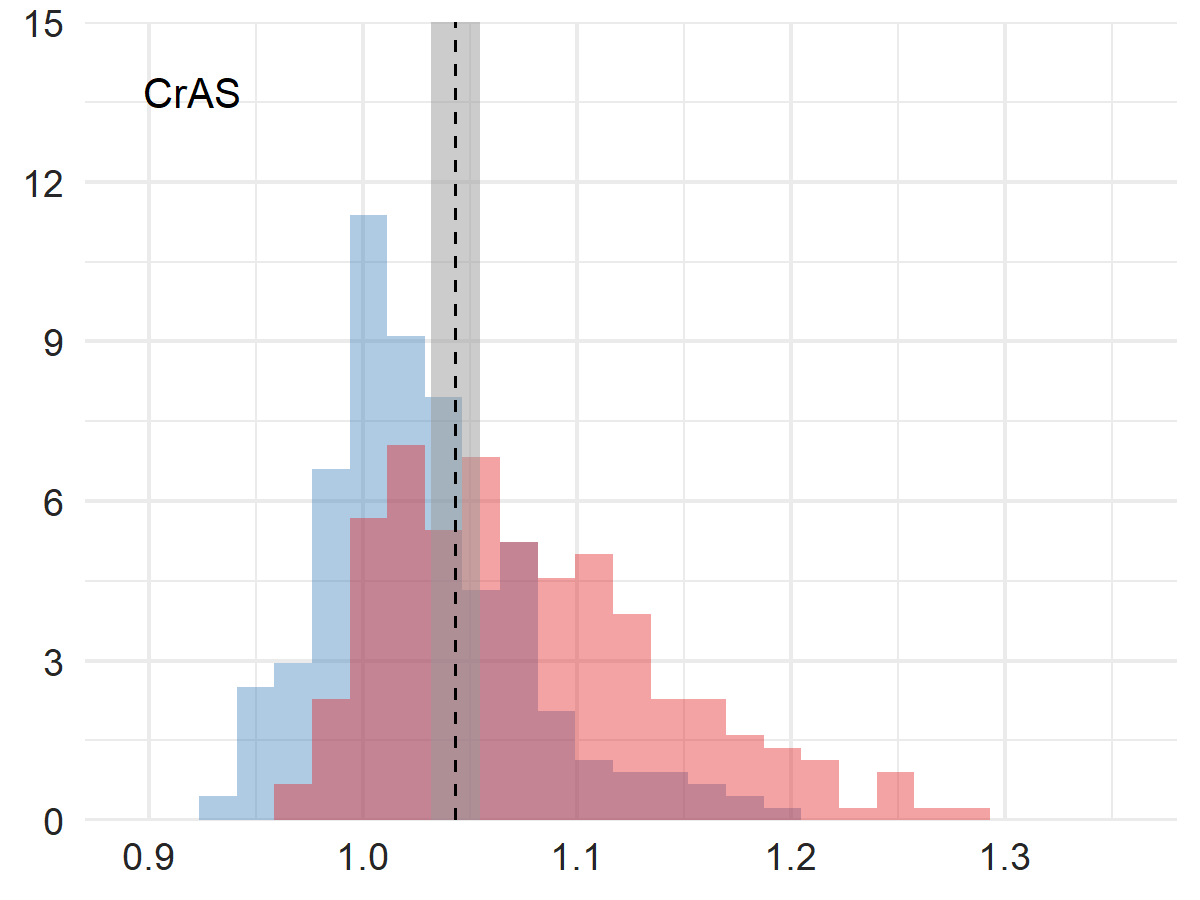

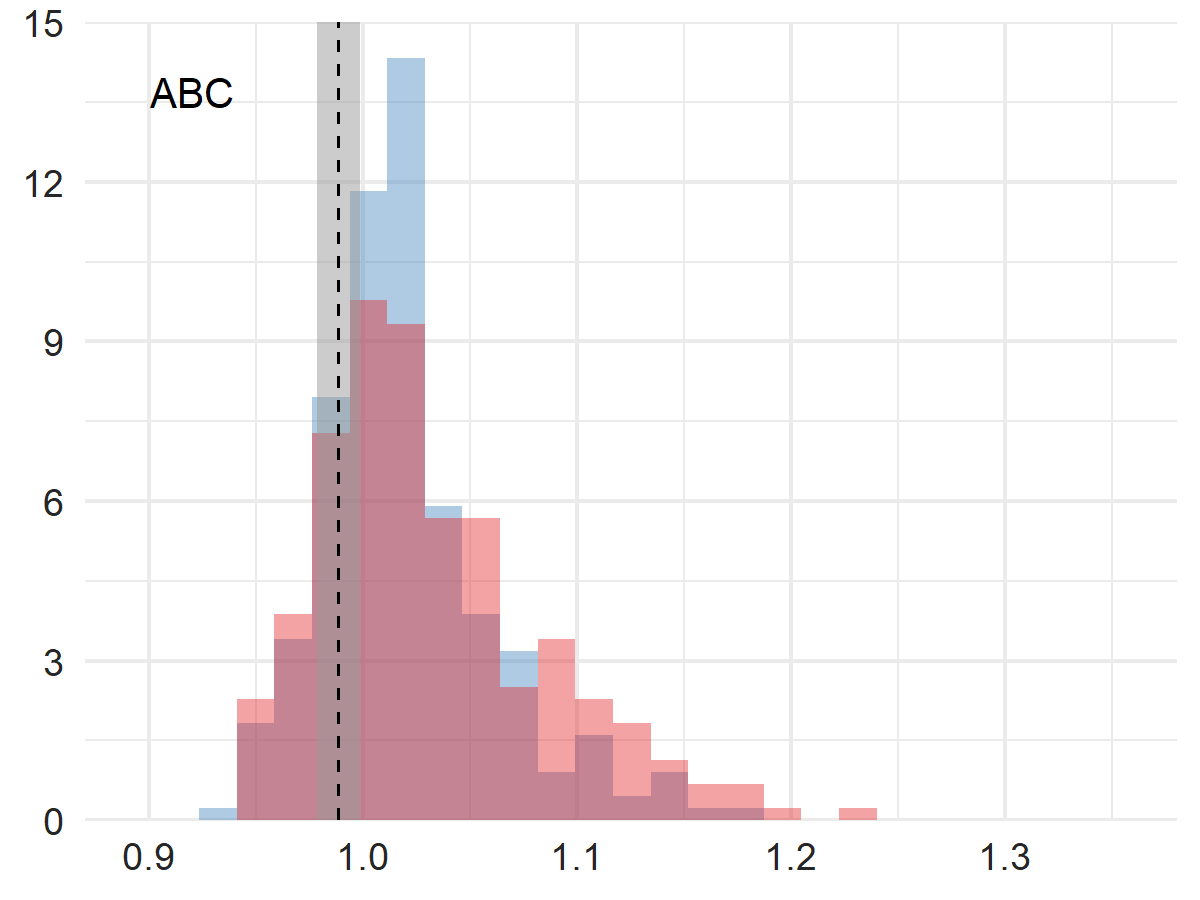

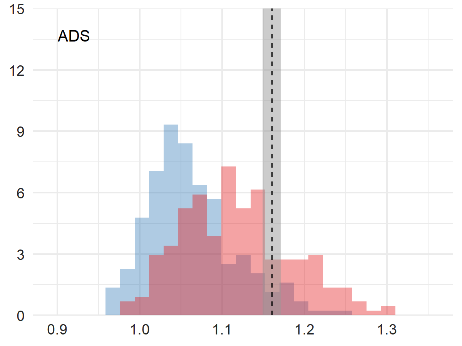

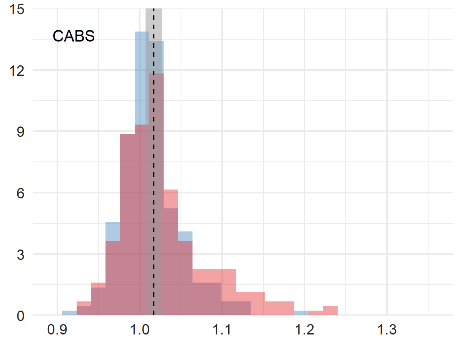

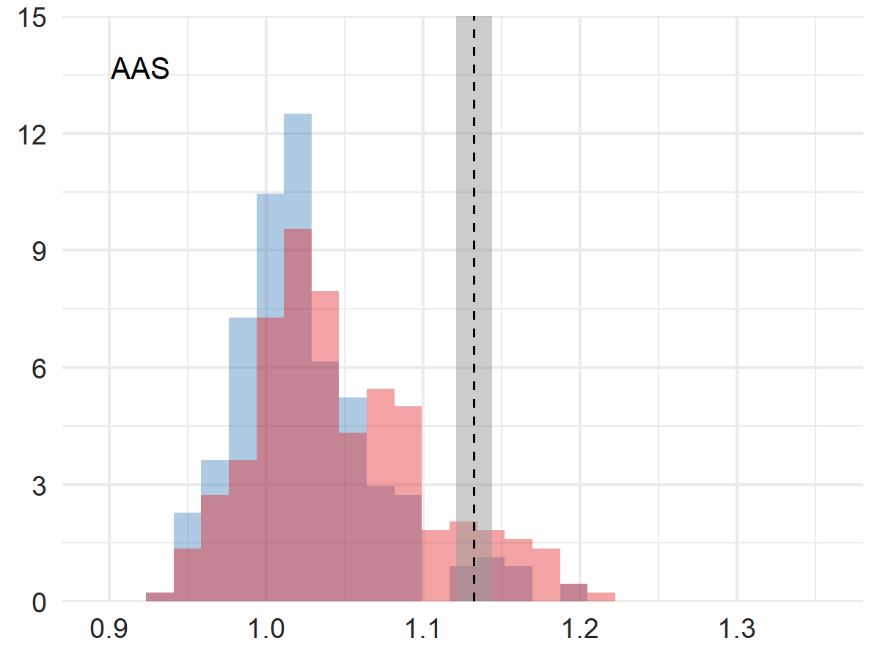

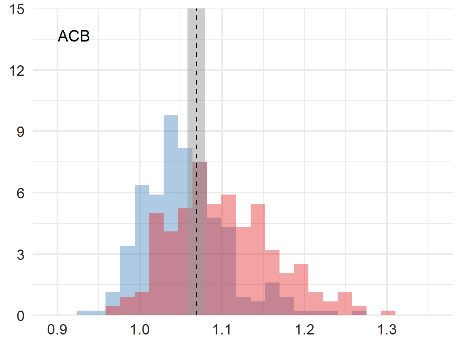

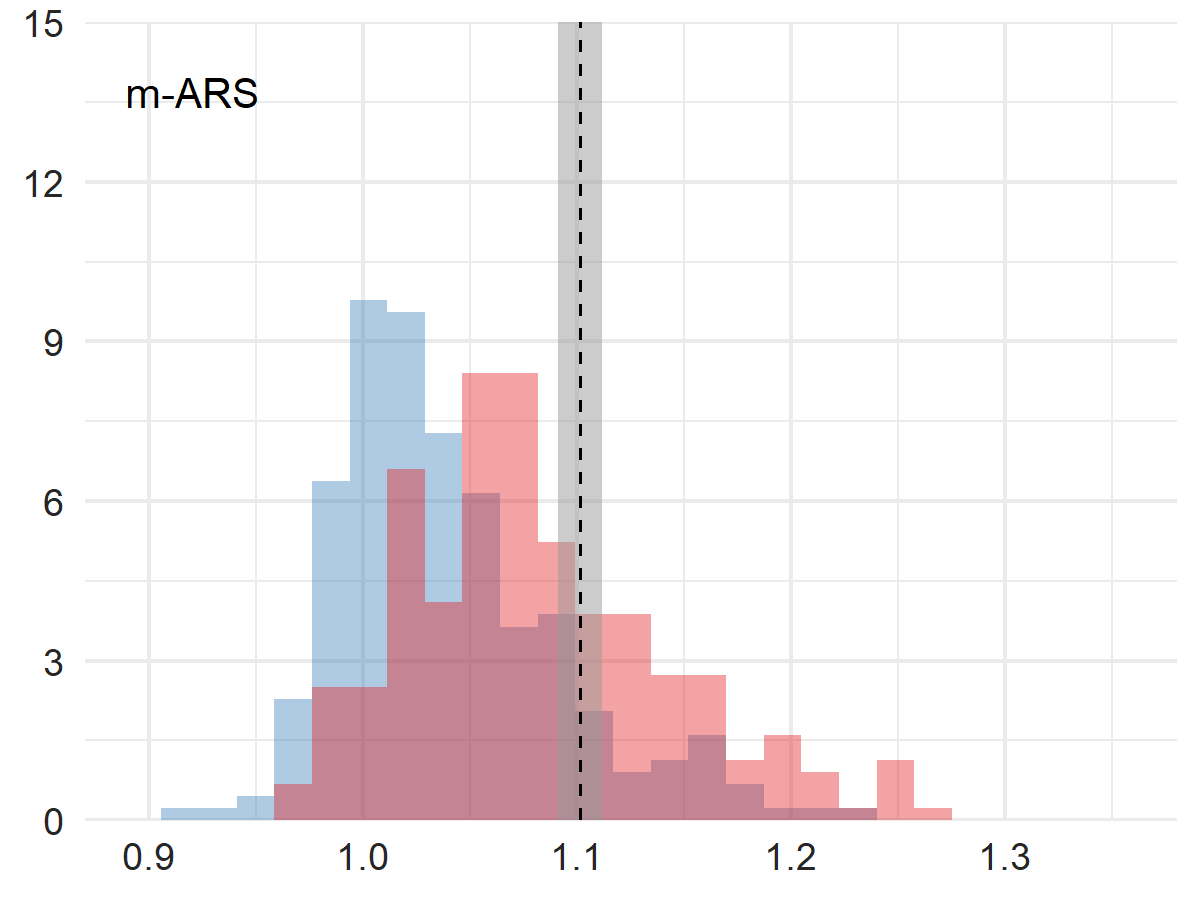

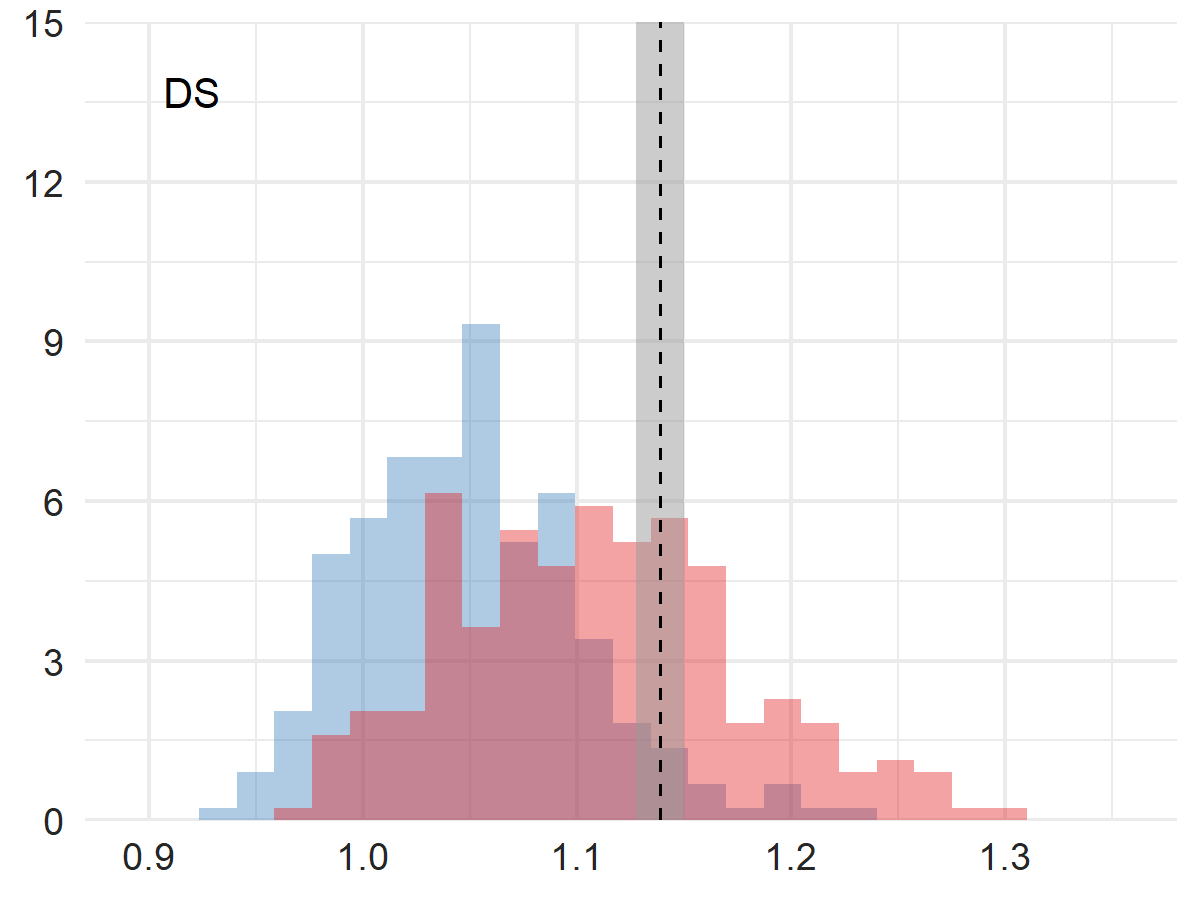

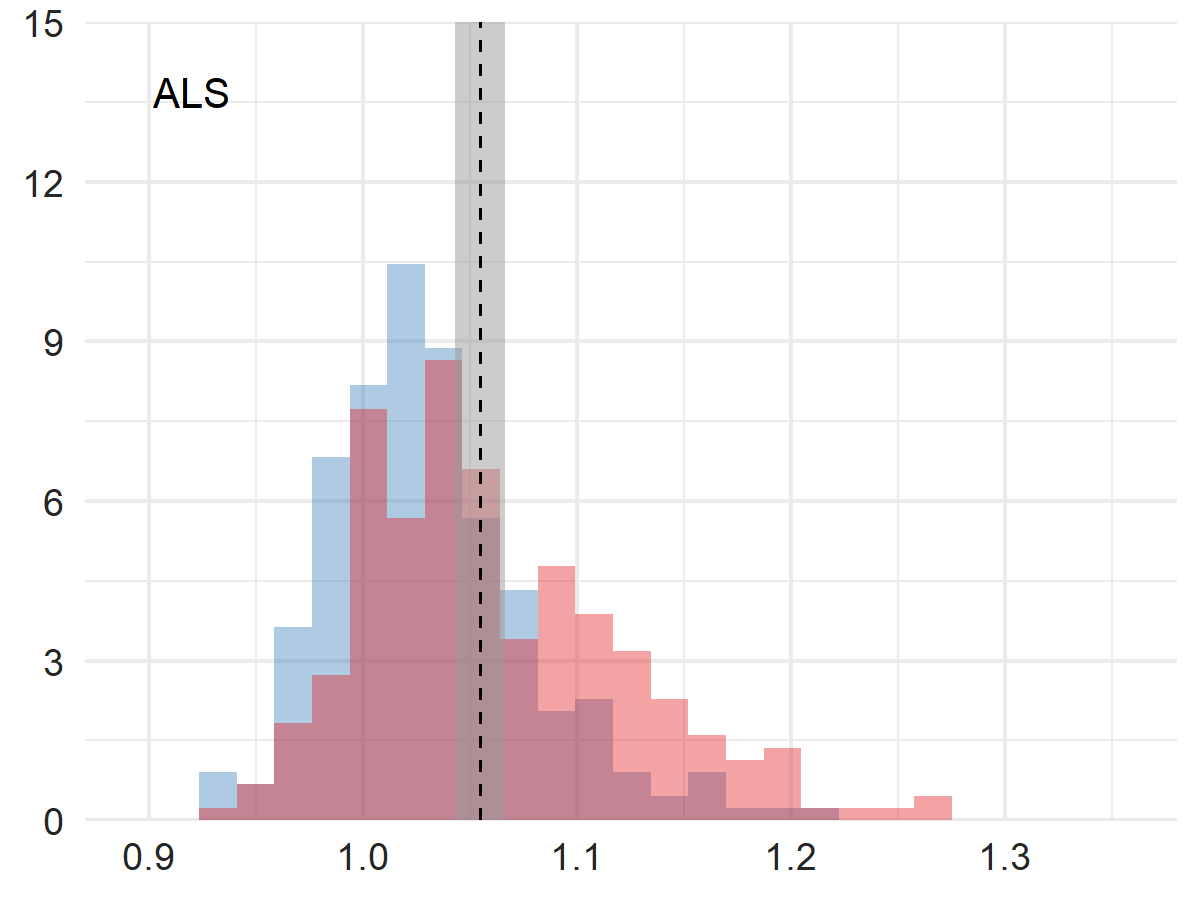

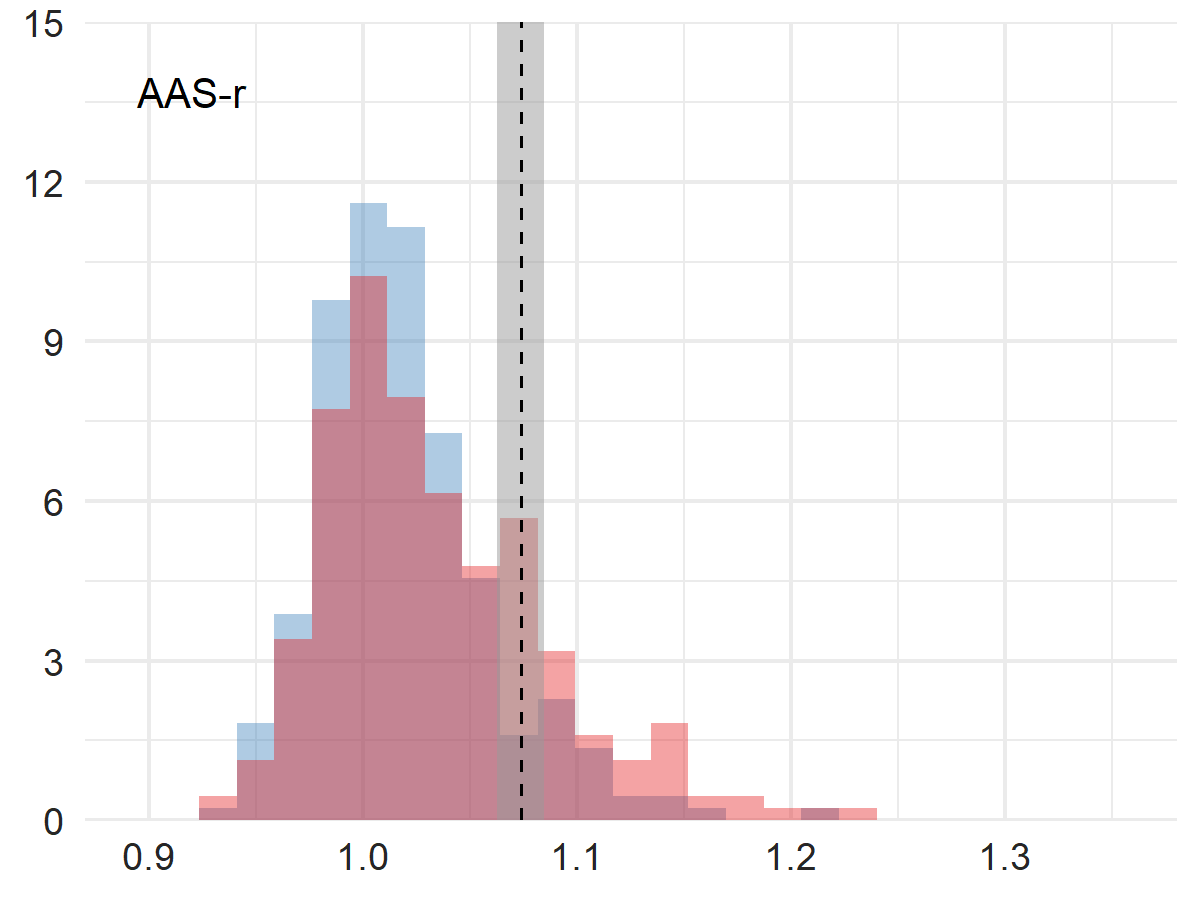

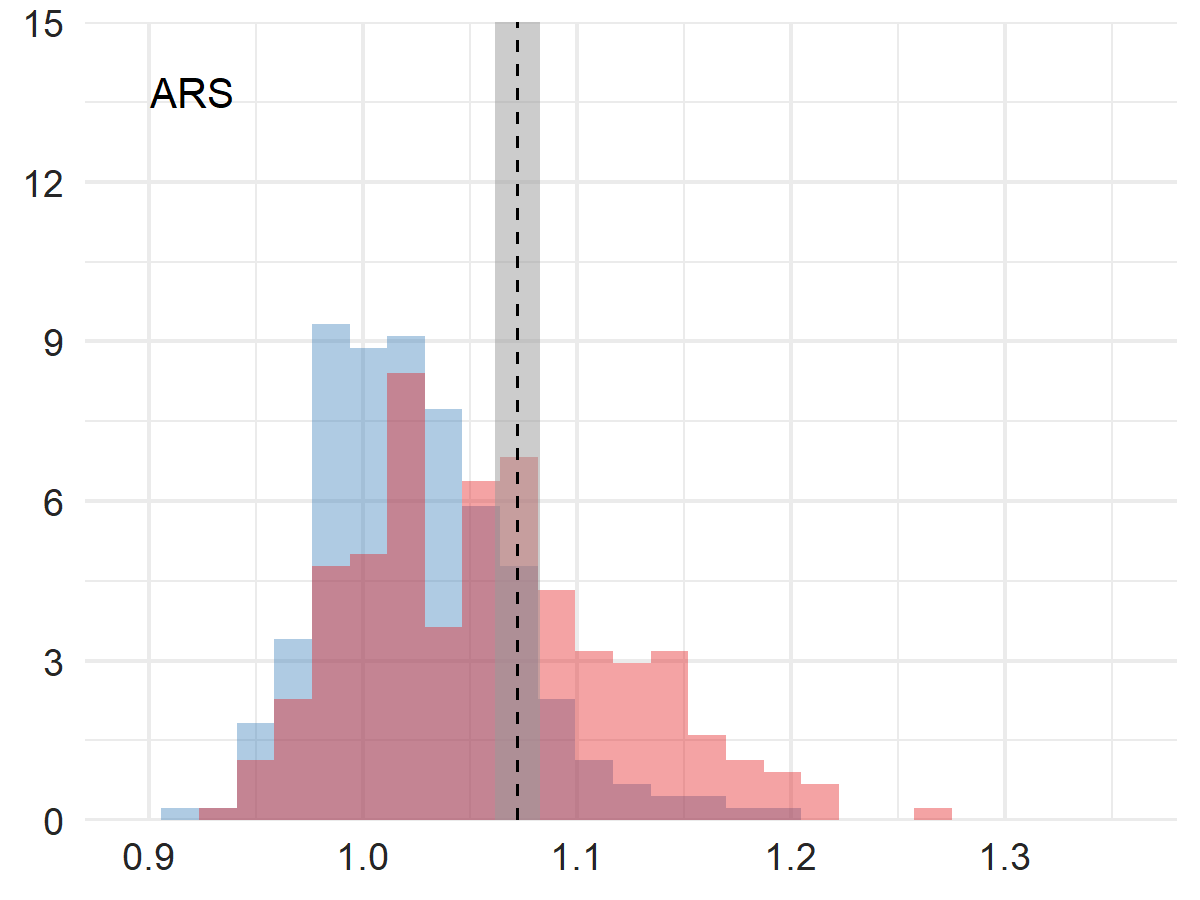

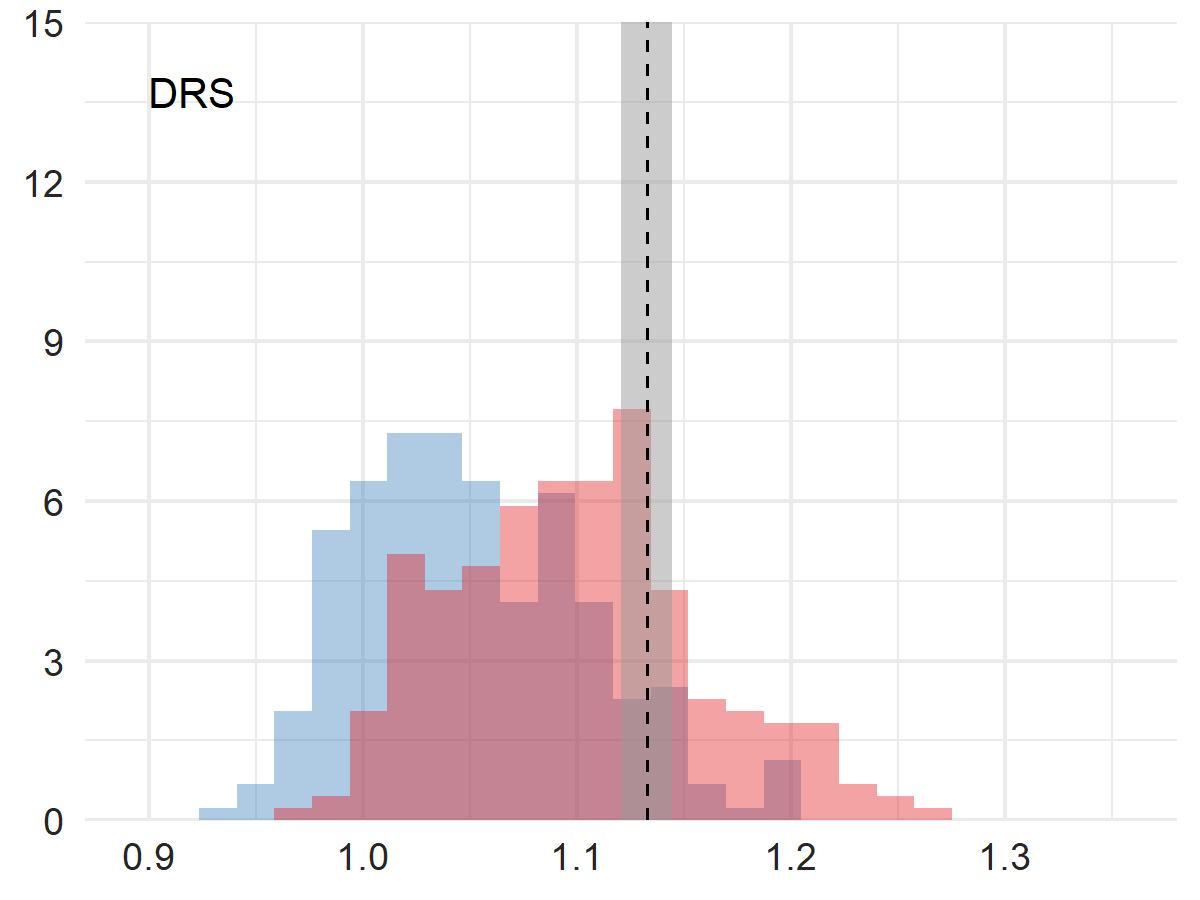

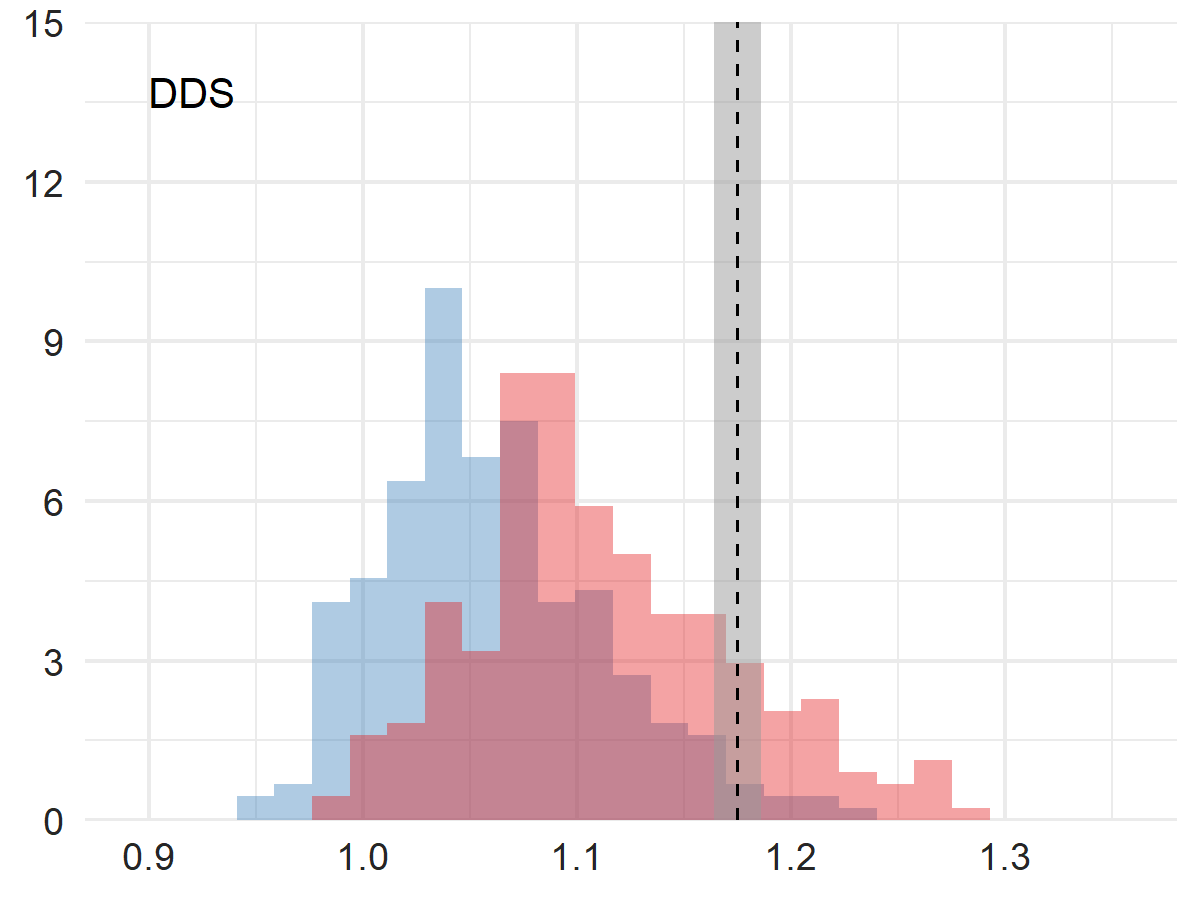

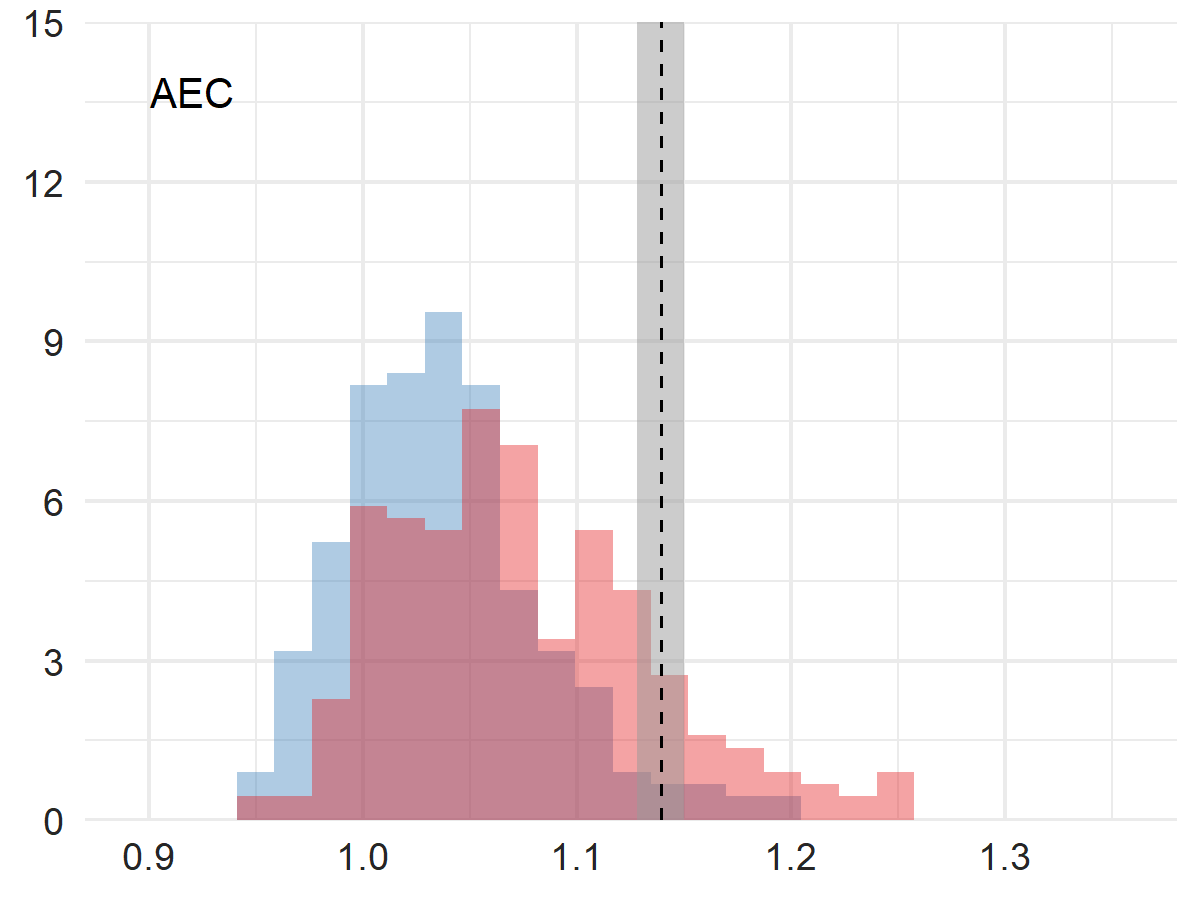

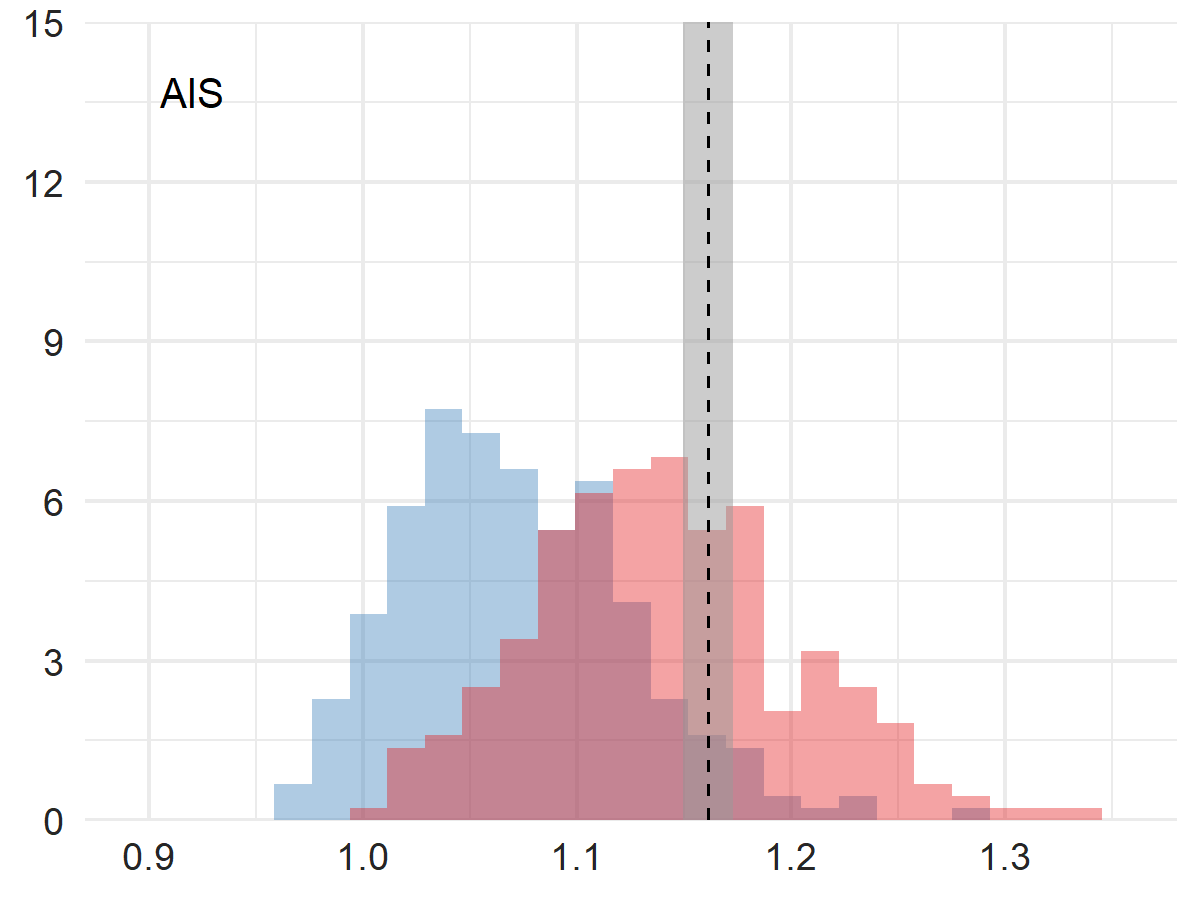

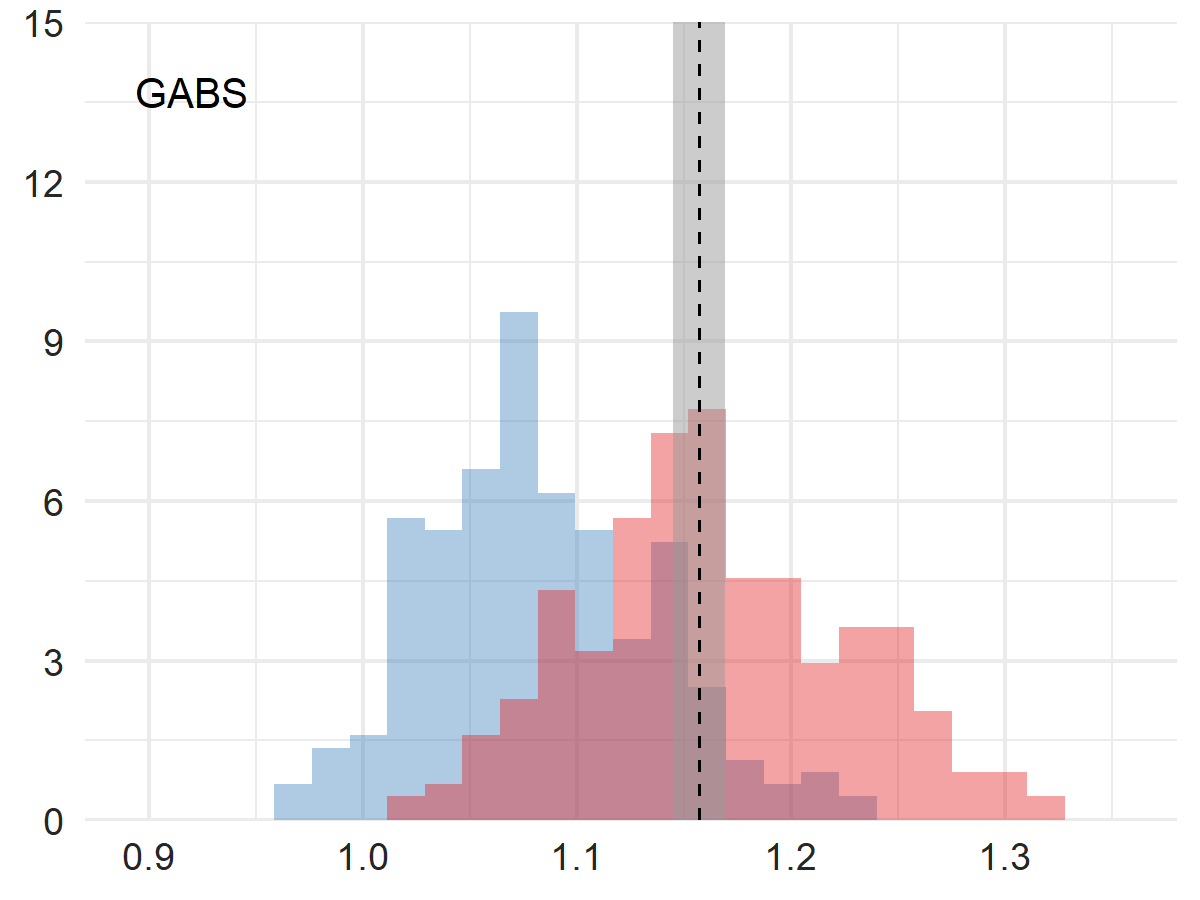

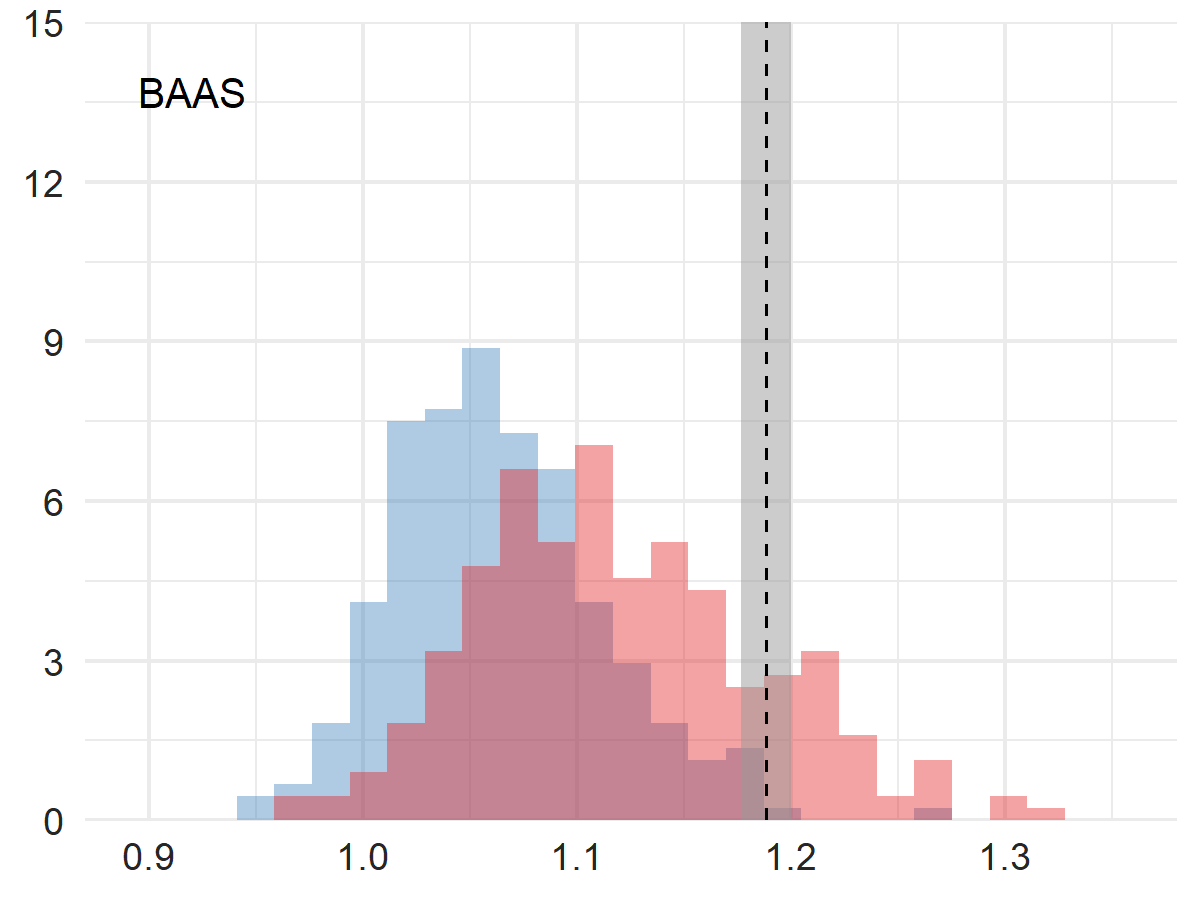

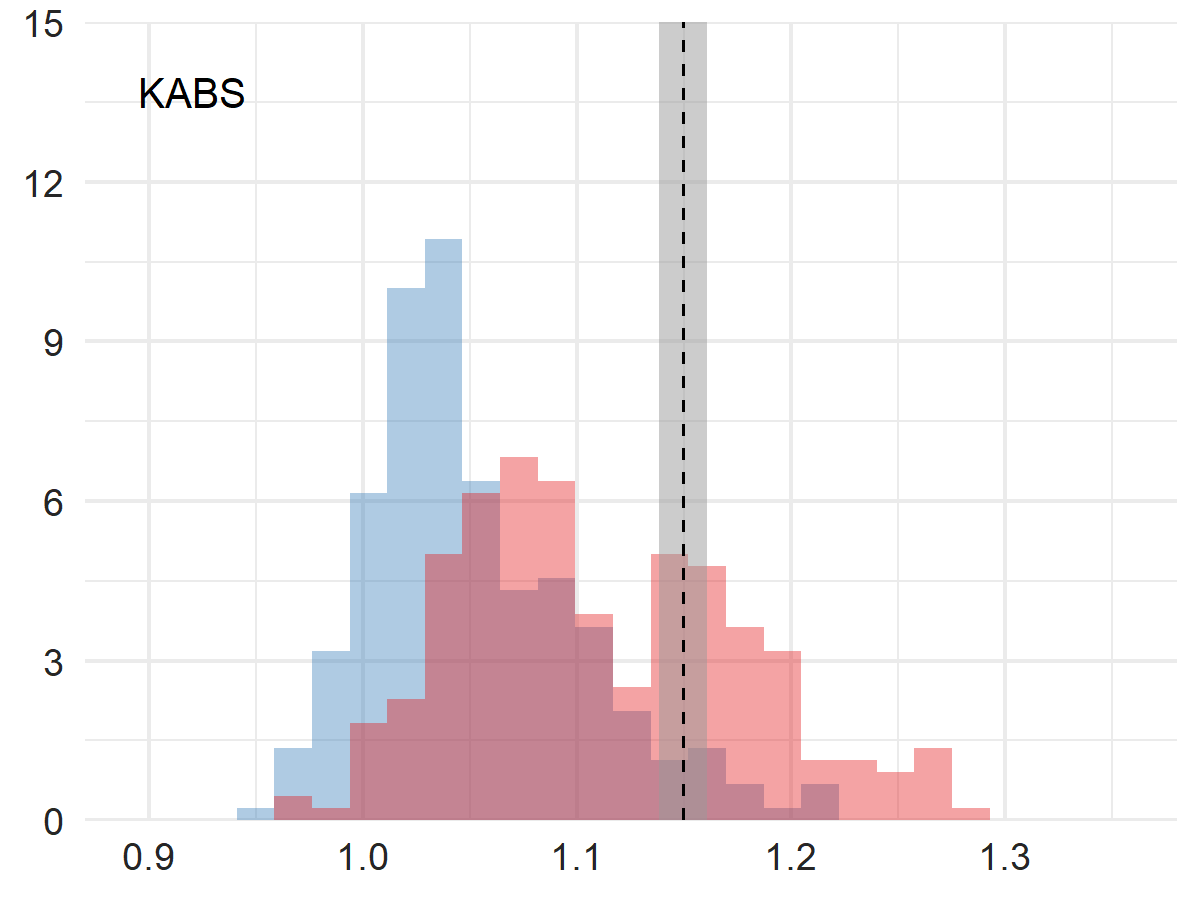

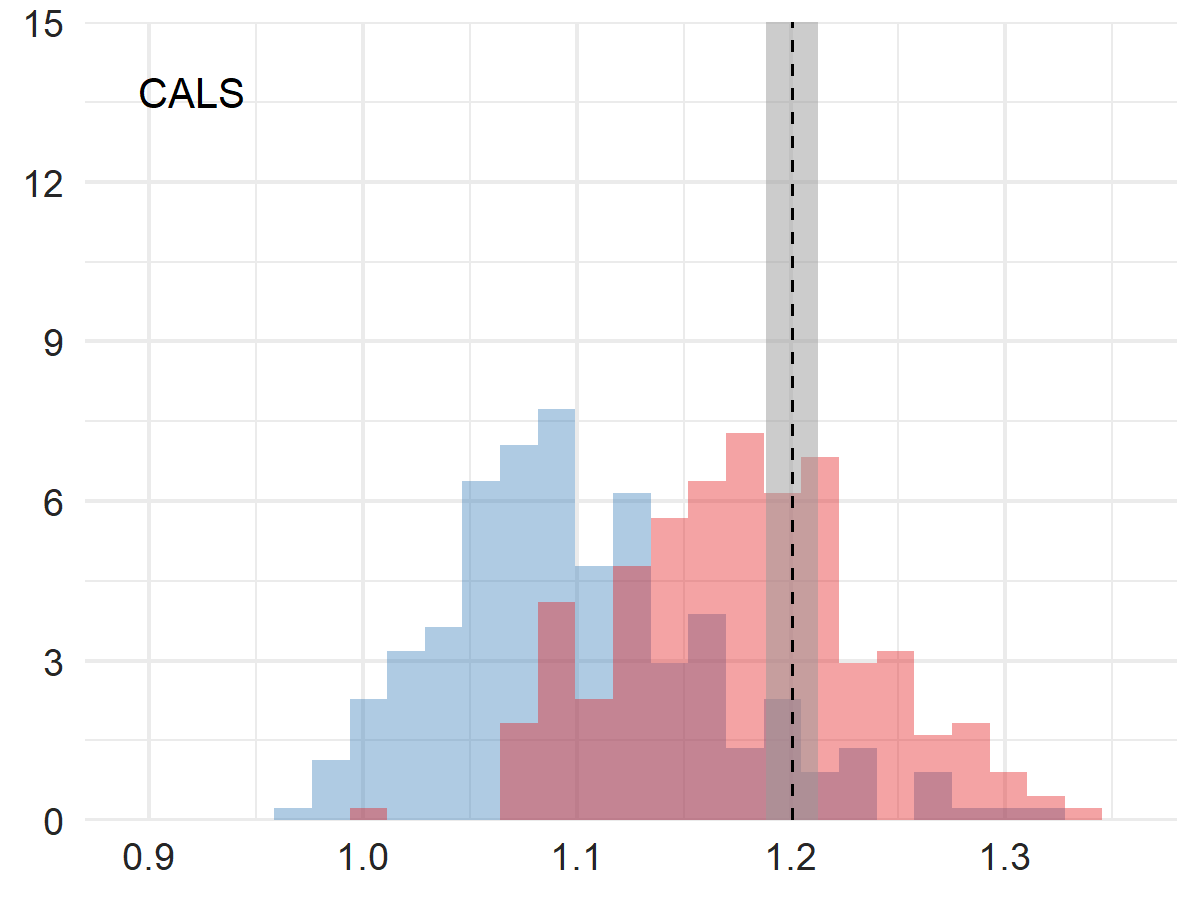

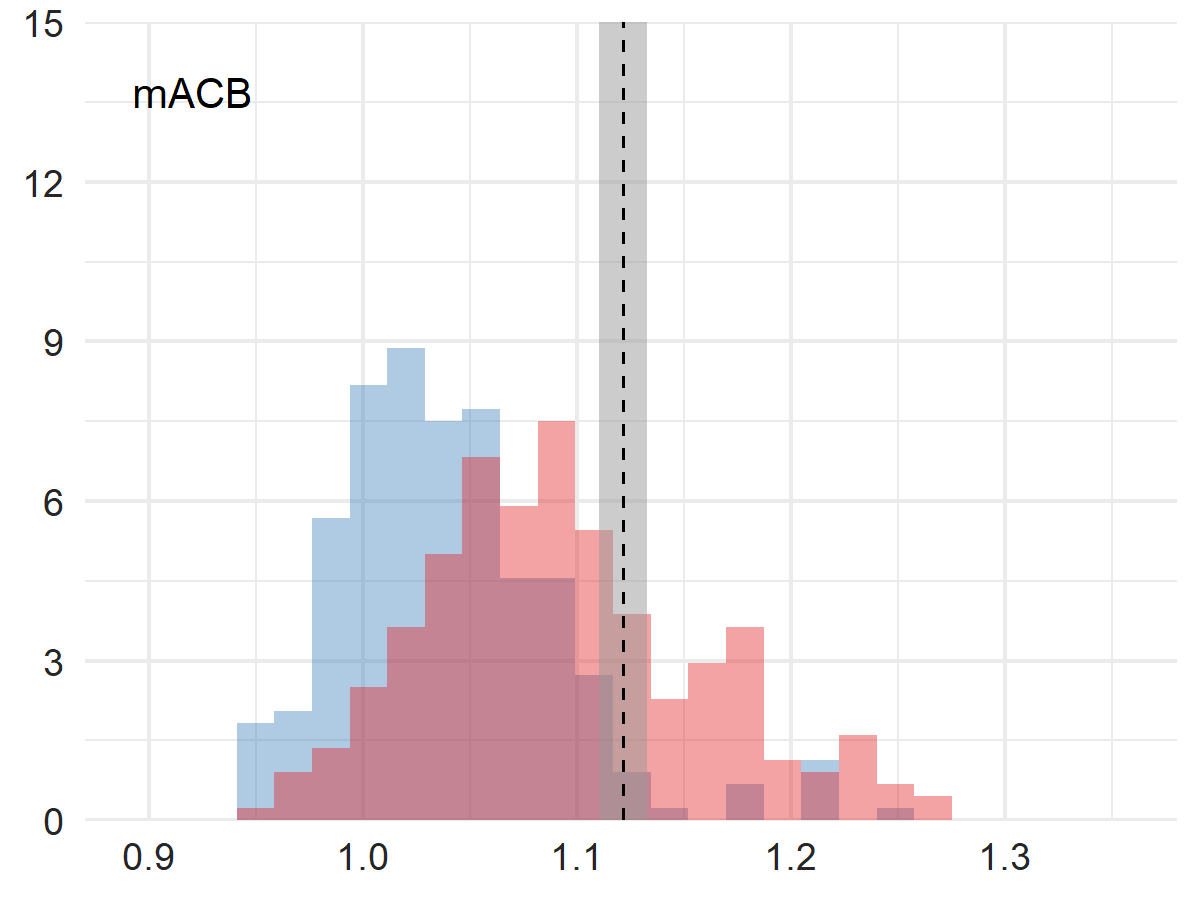

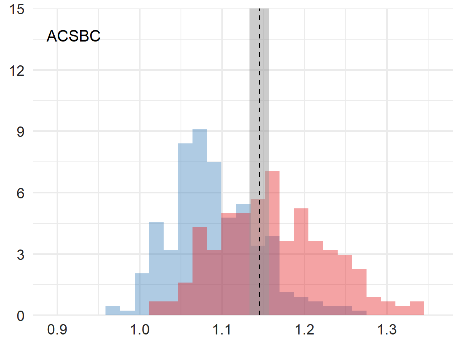

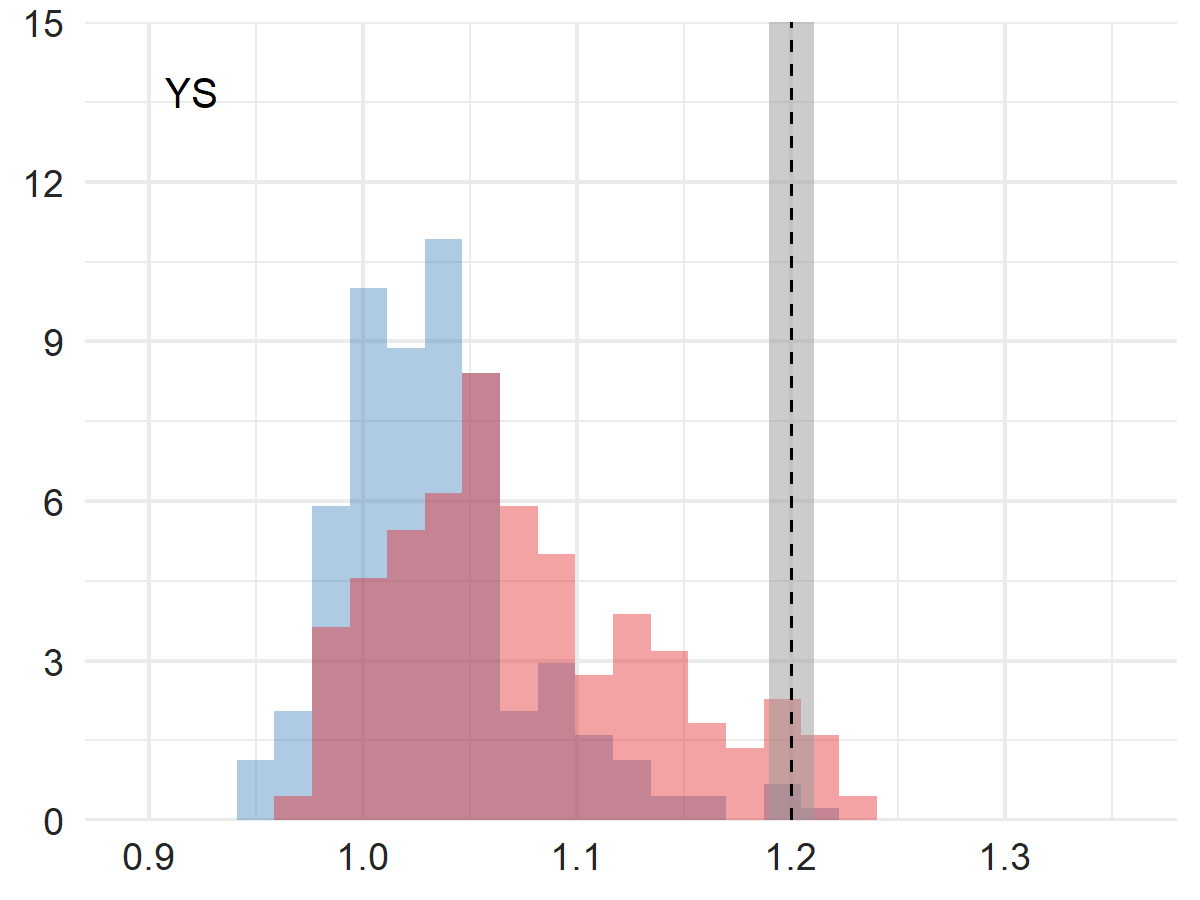


*Delirium*


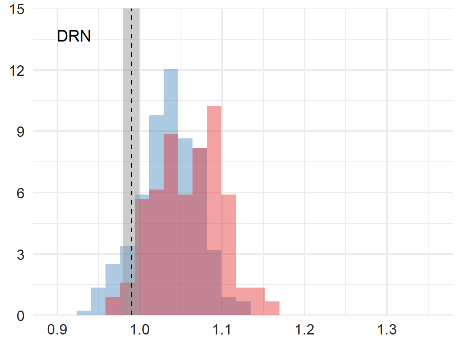

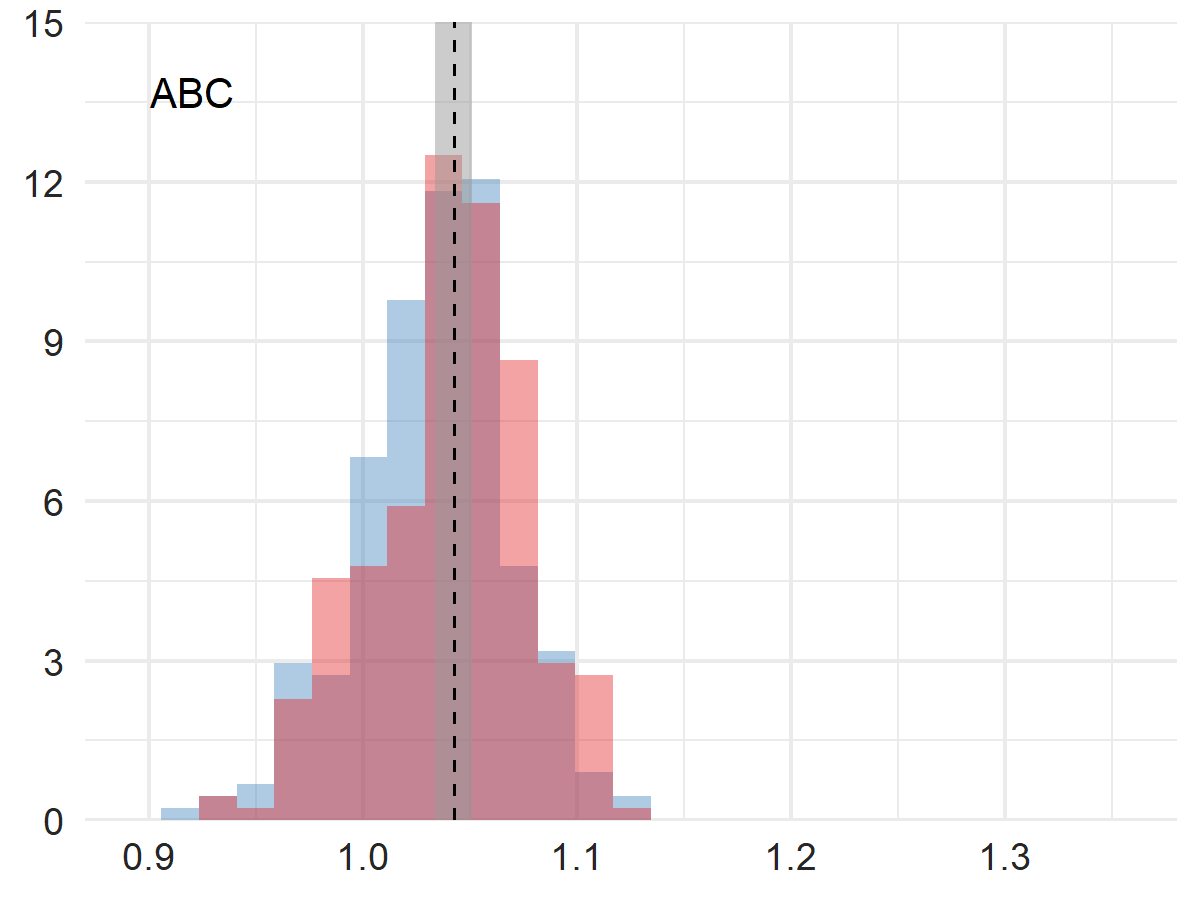

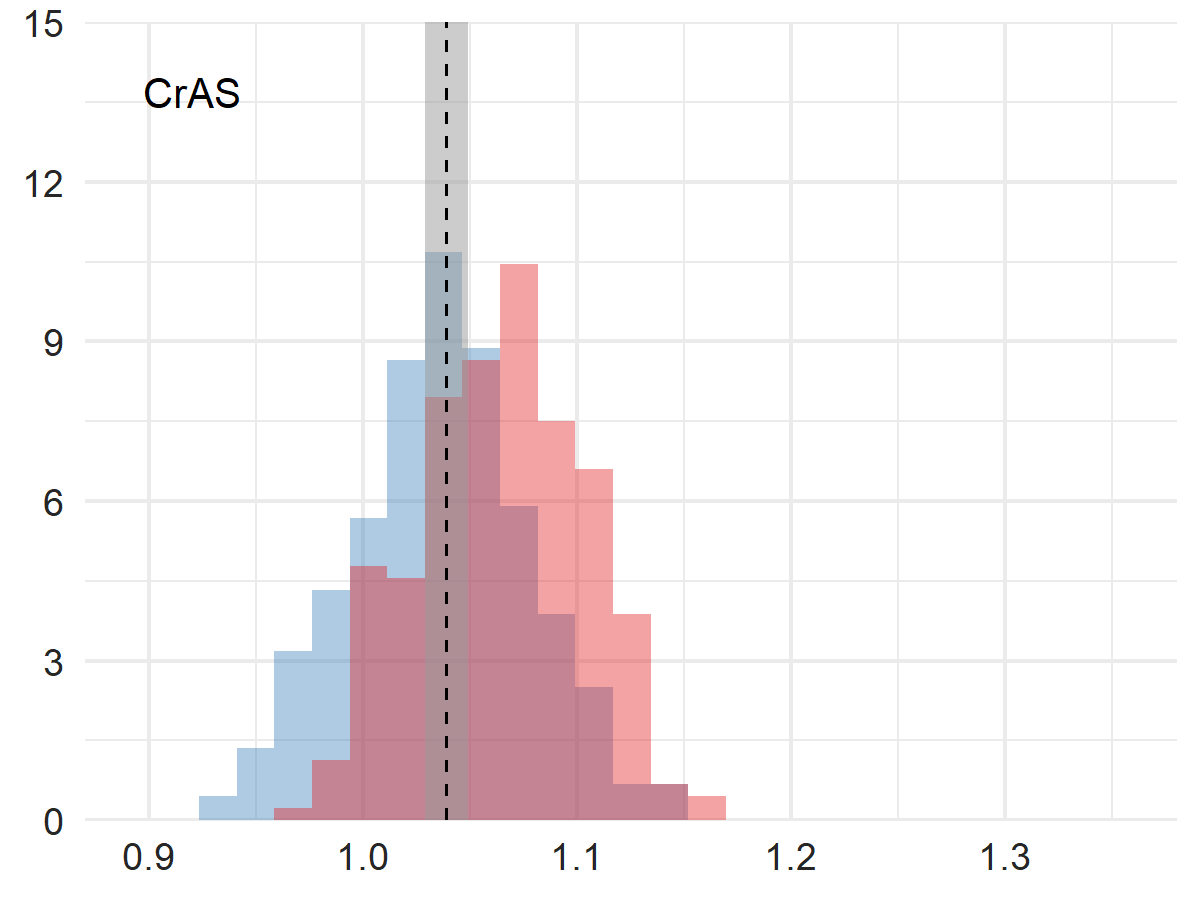

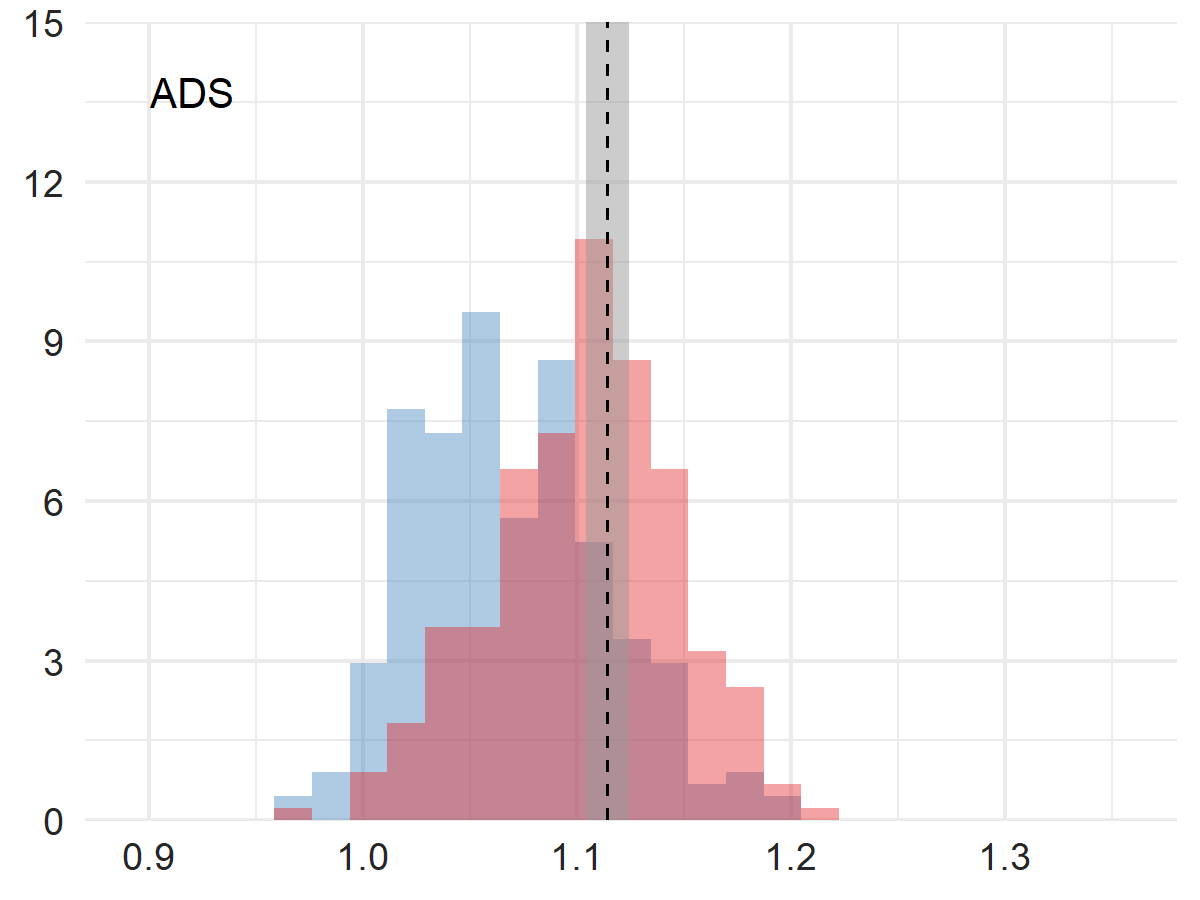

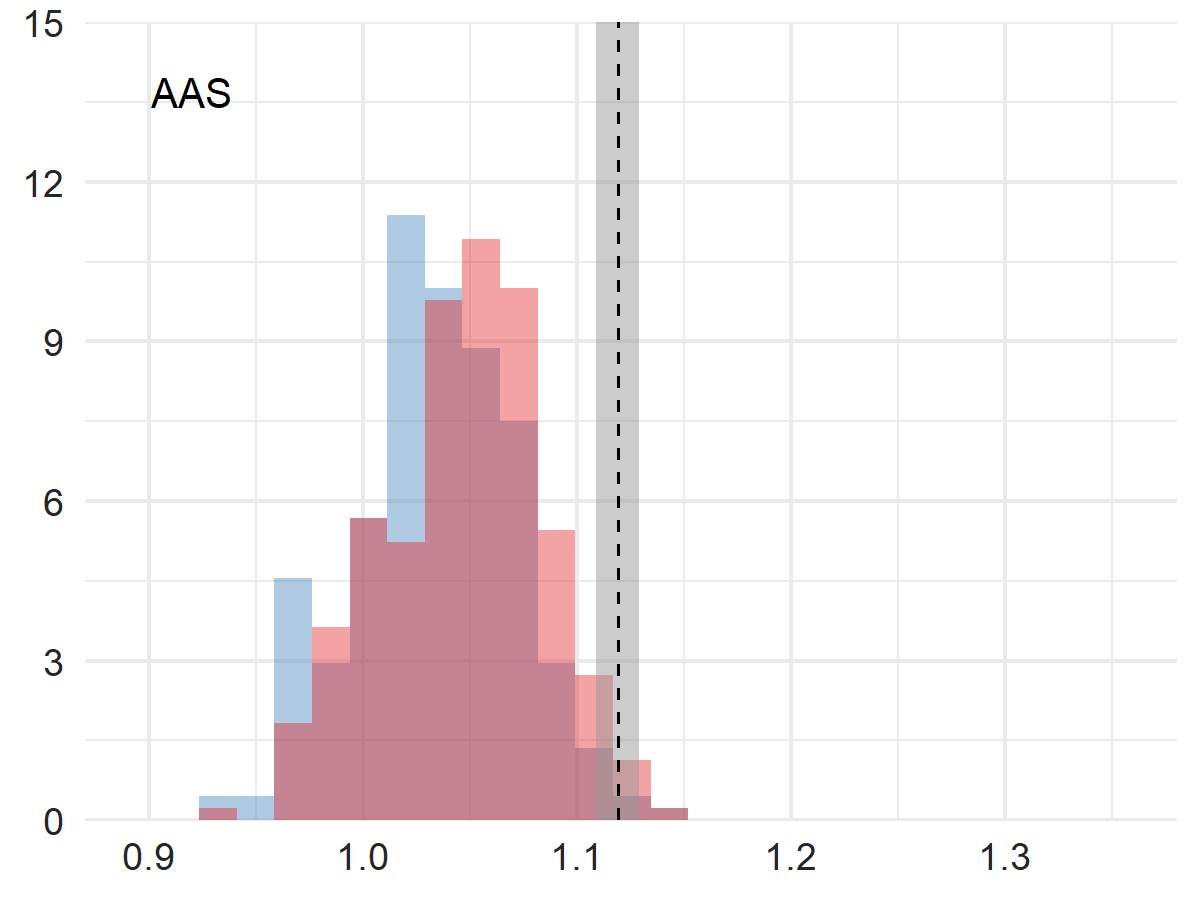

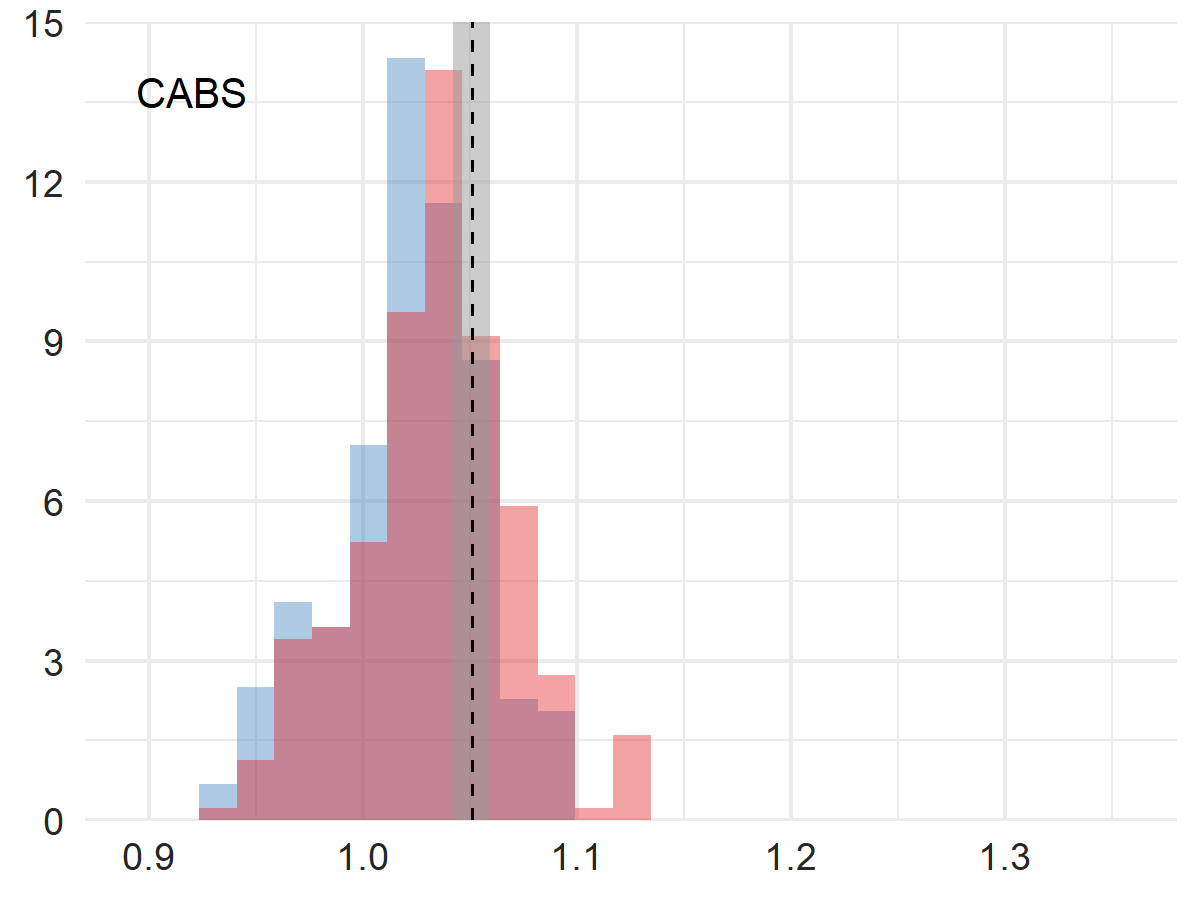

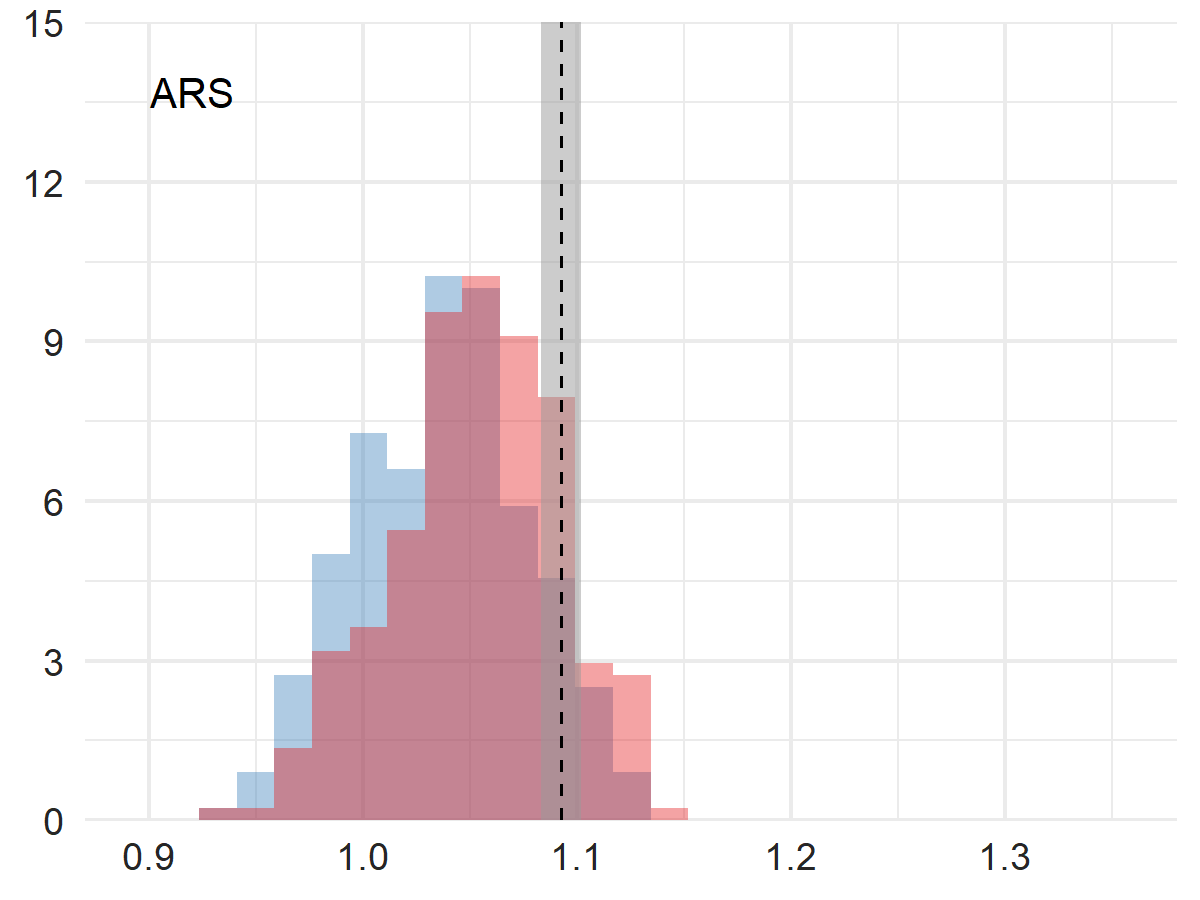

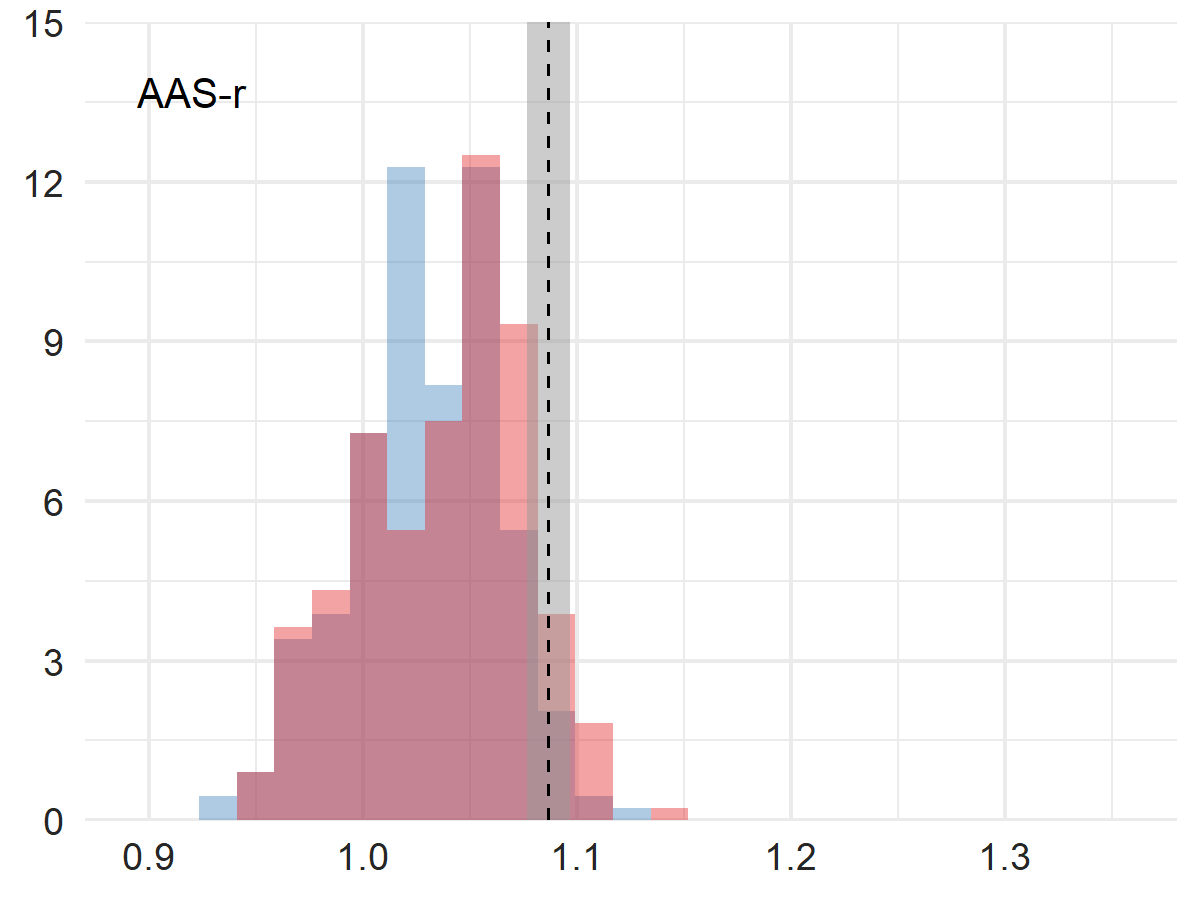

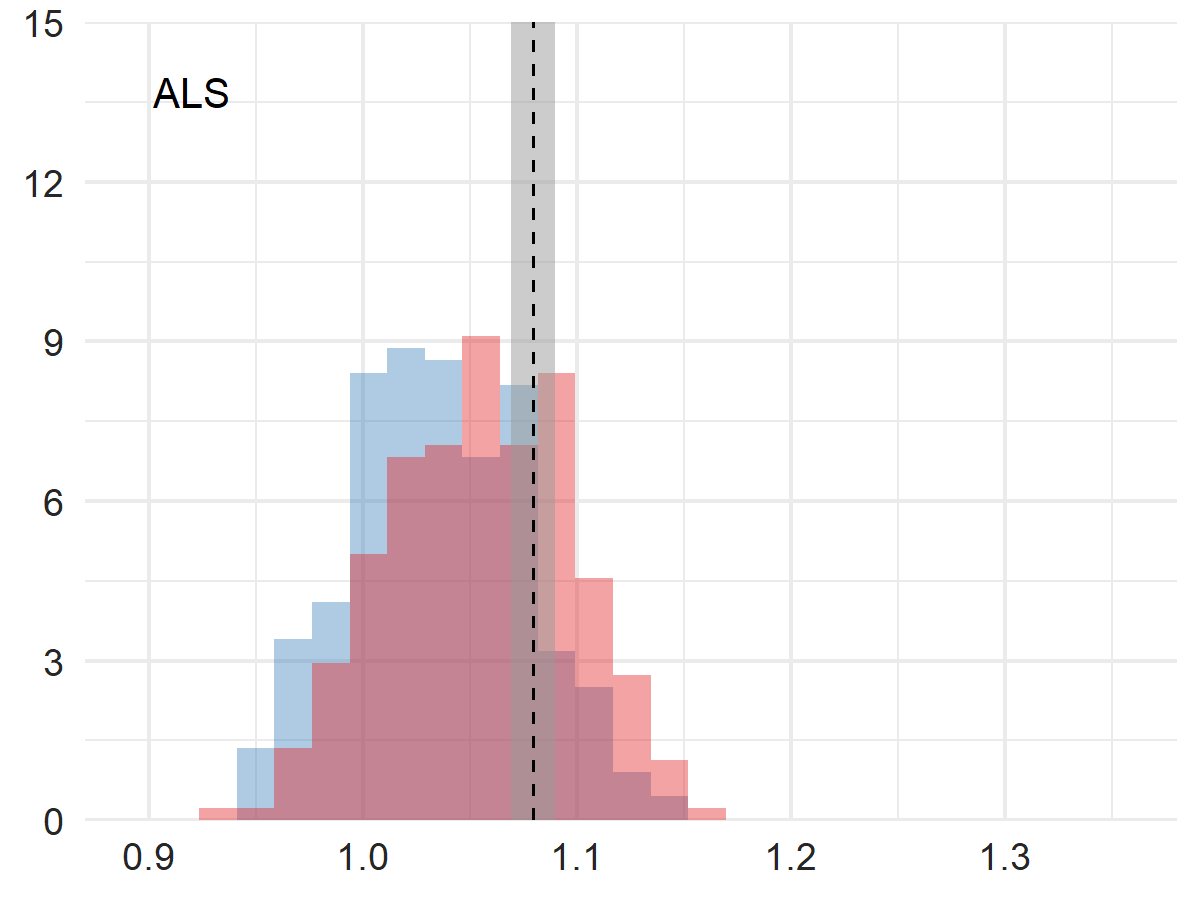

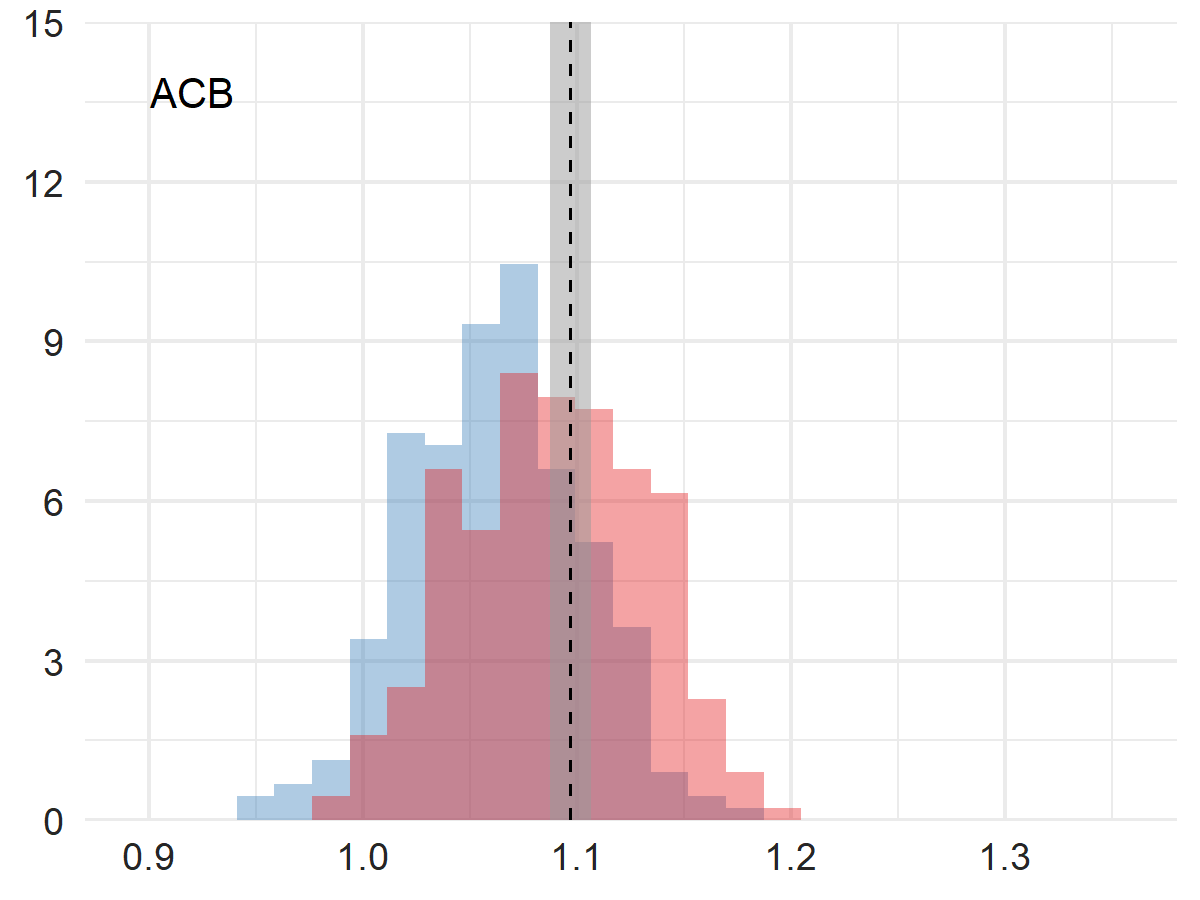

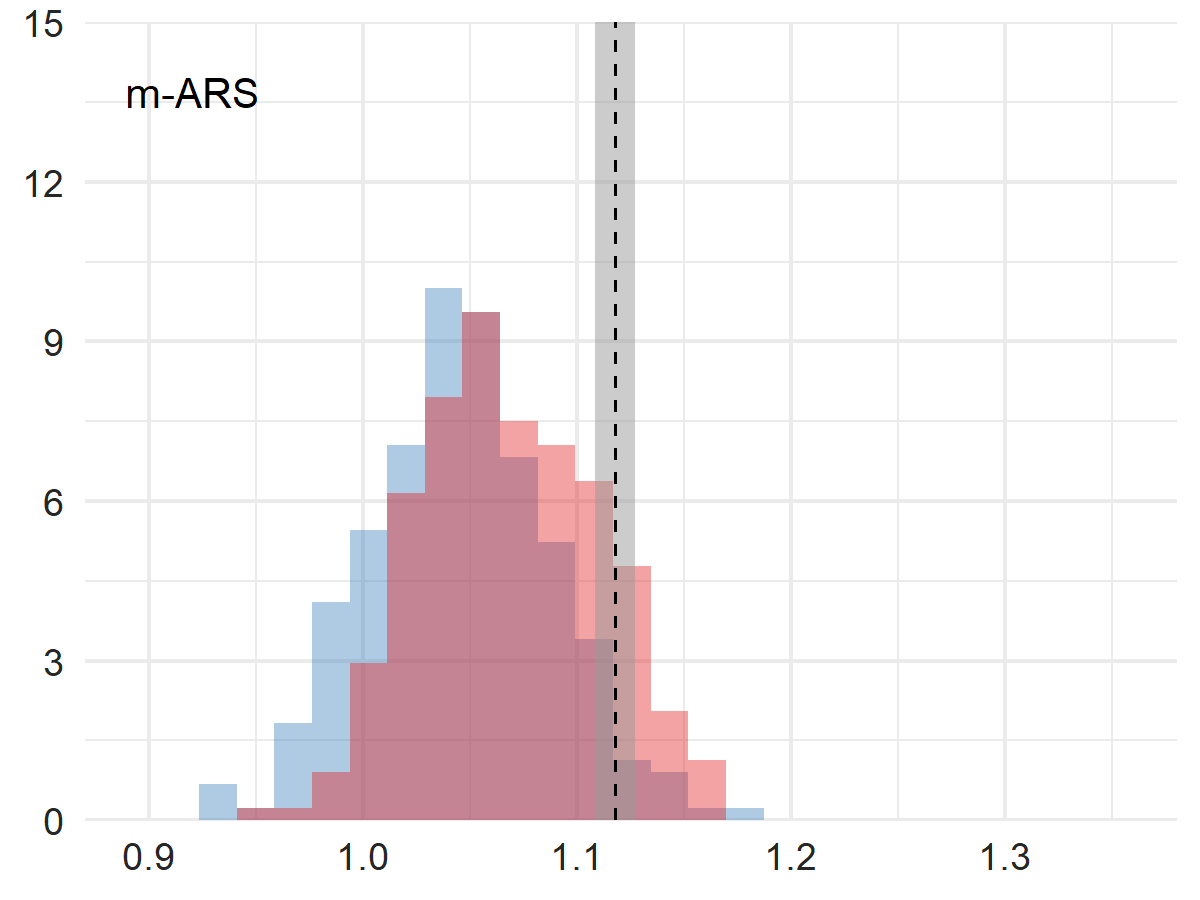

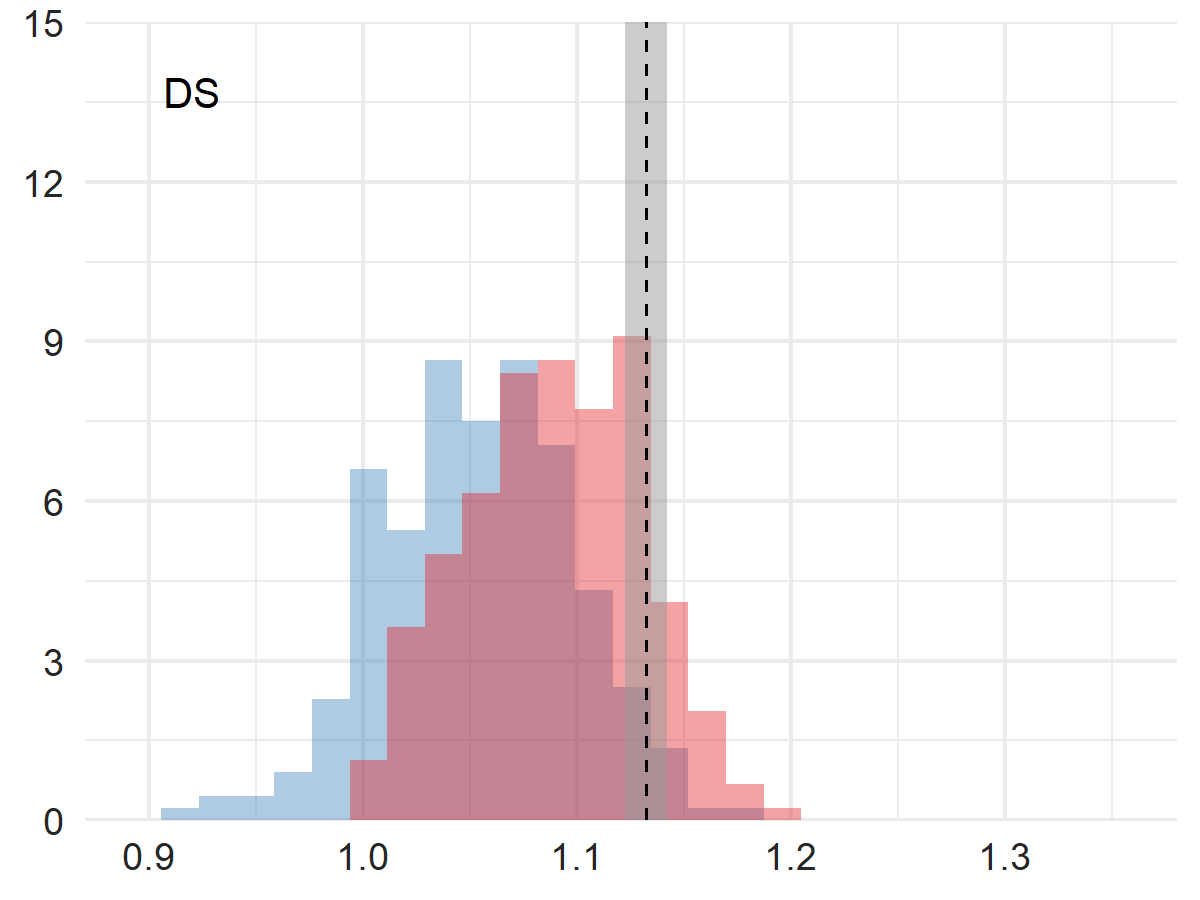

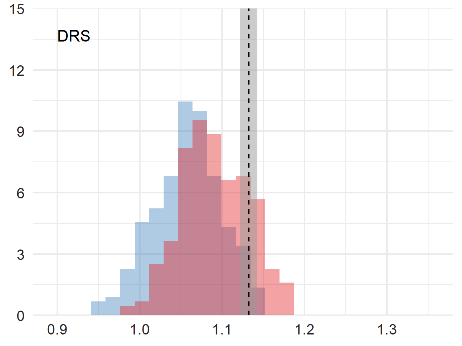

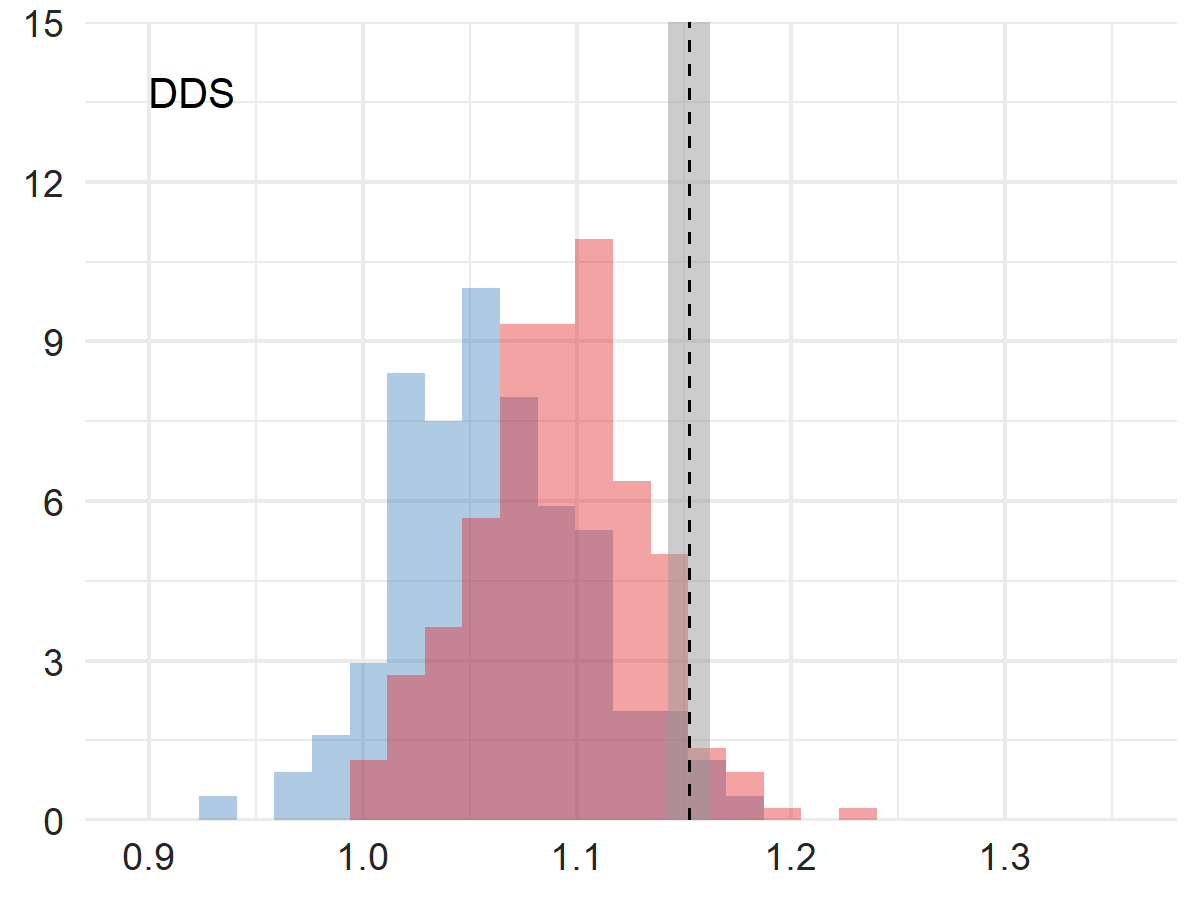

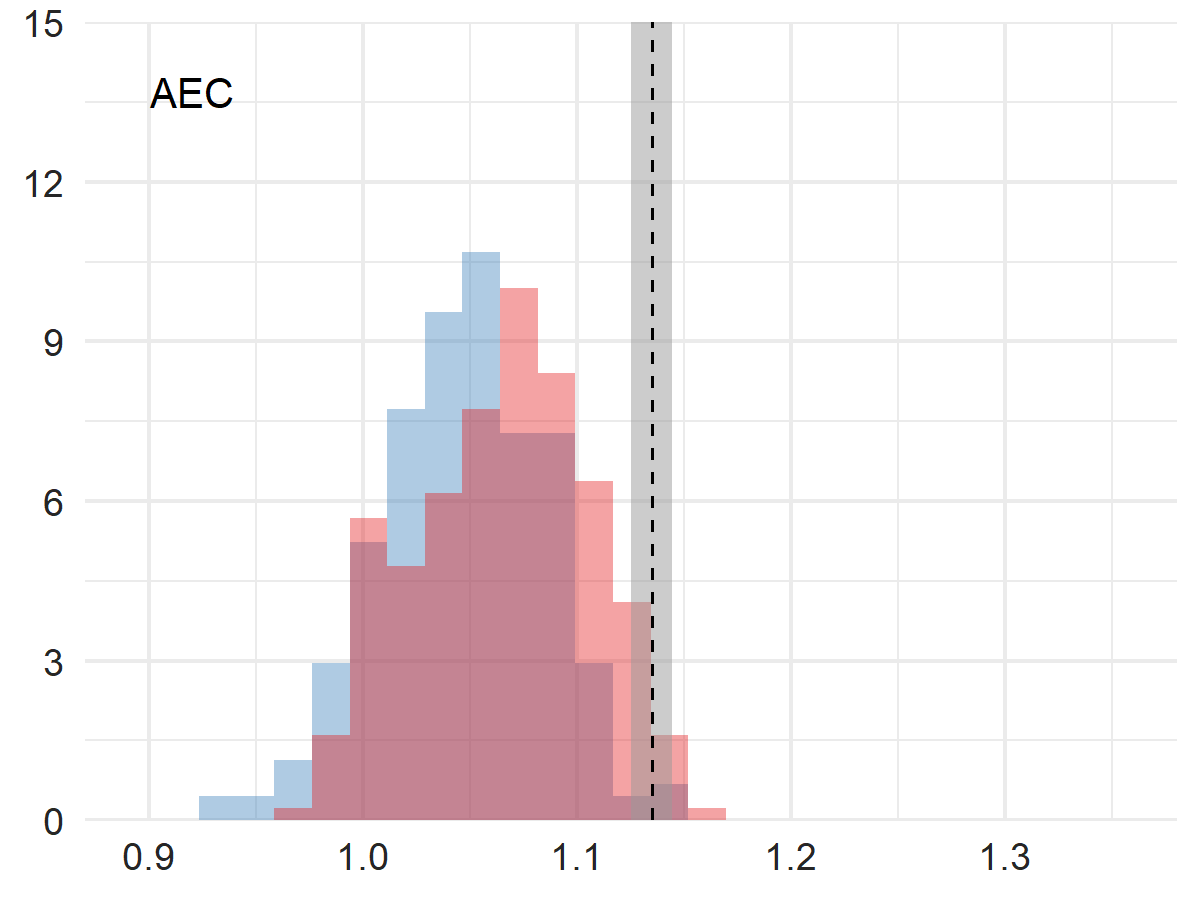

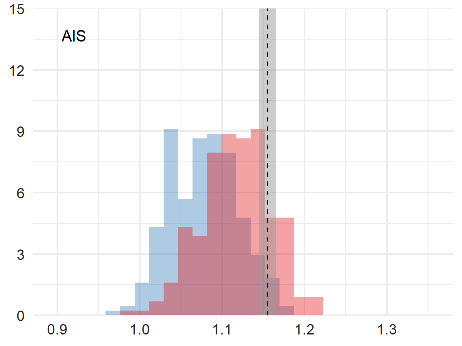

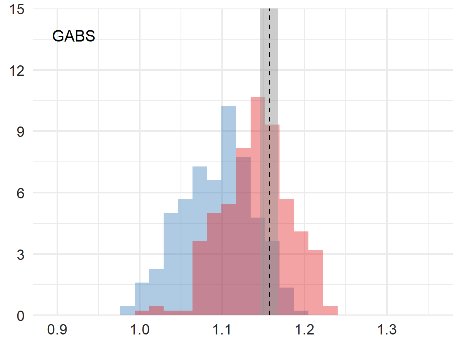

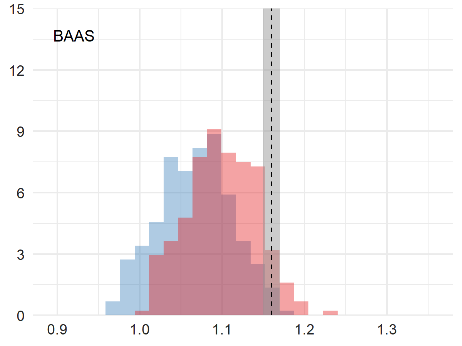

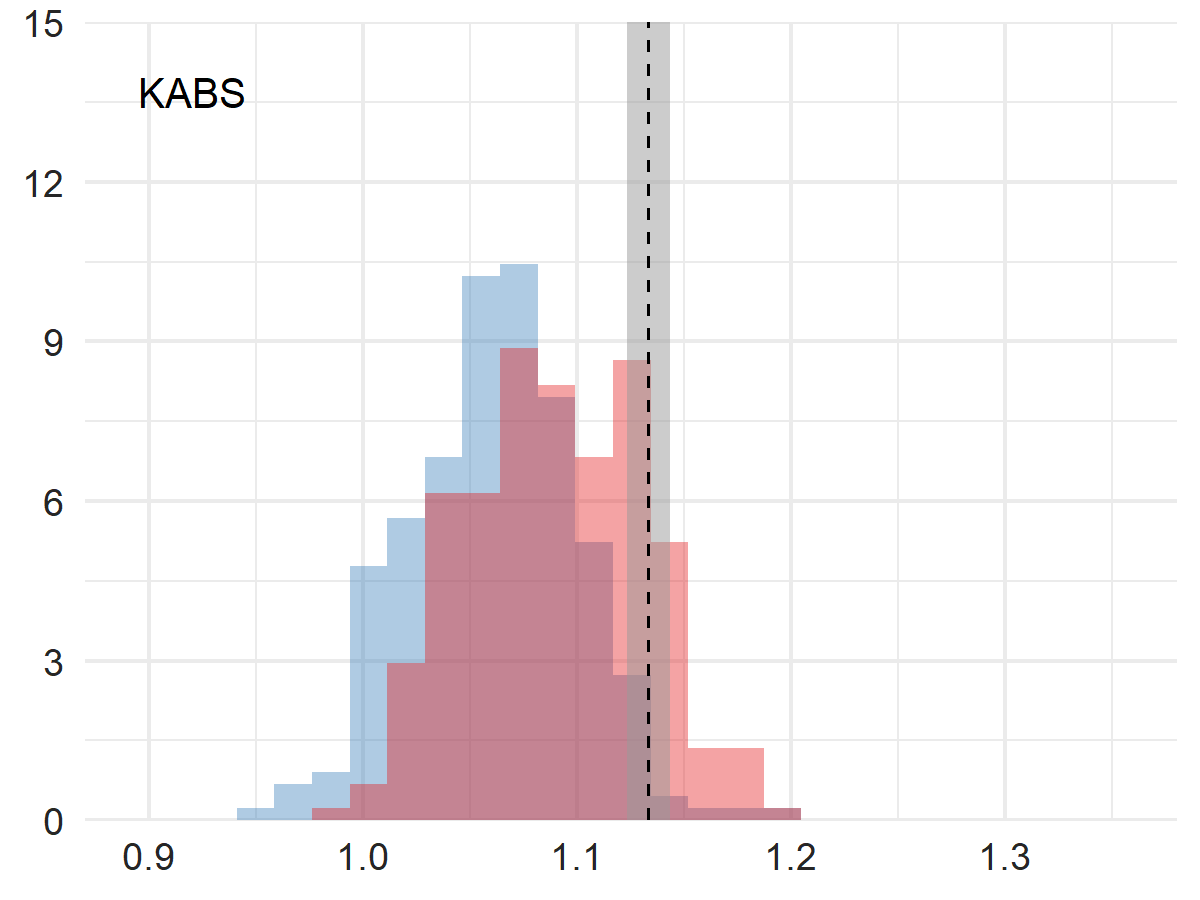

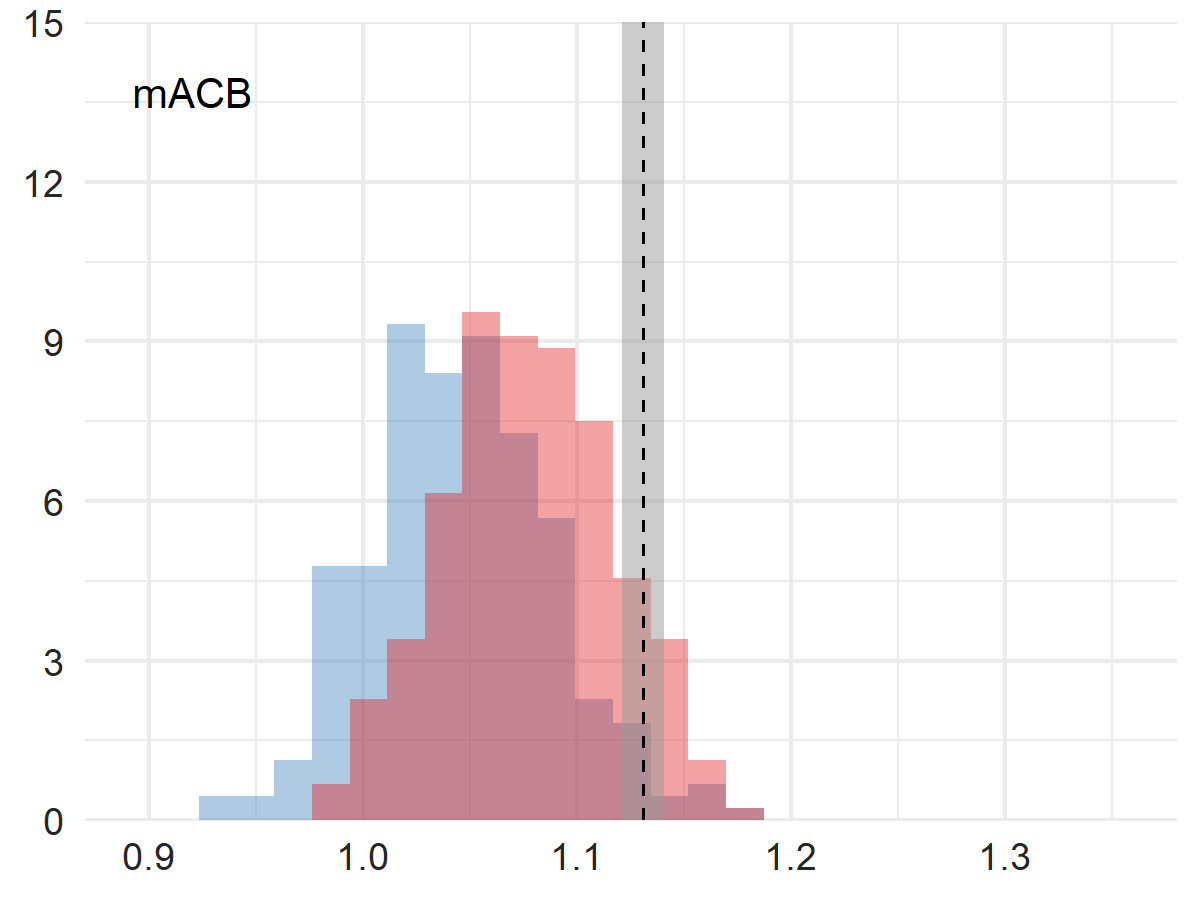

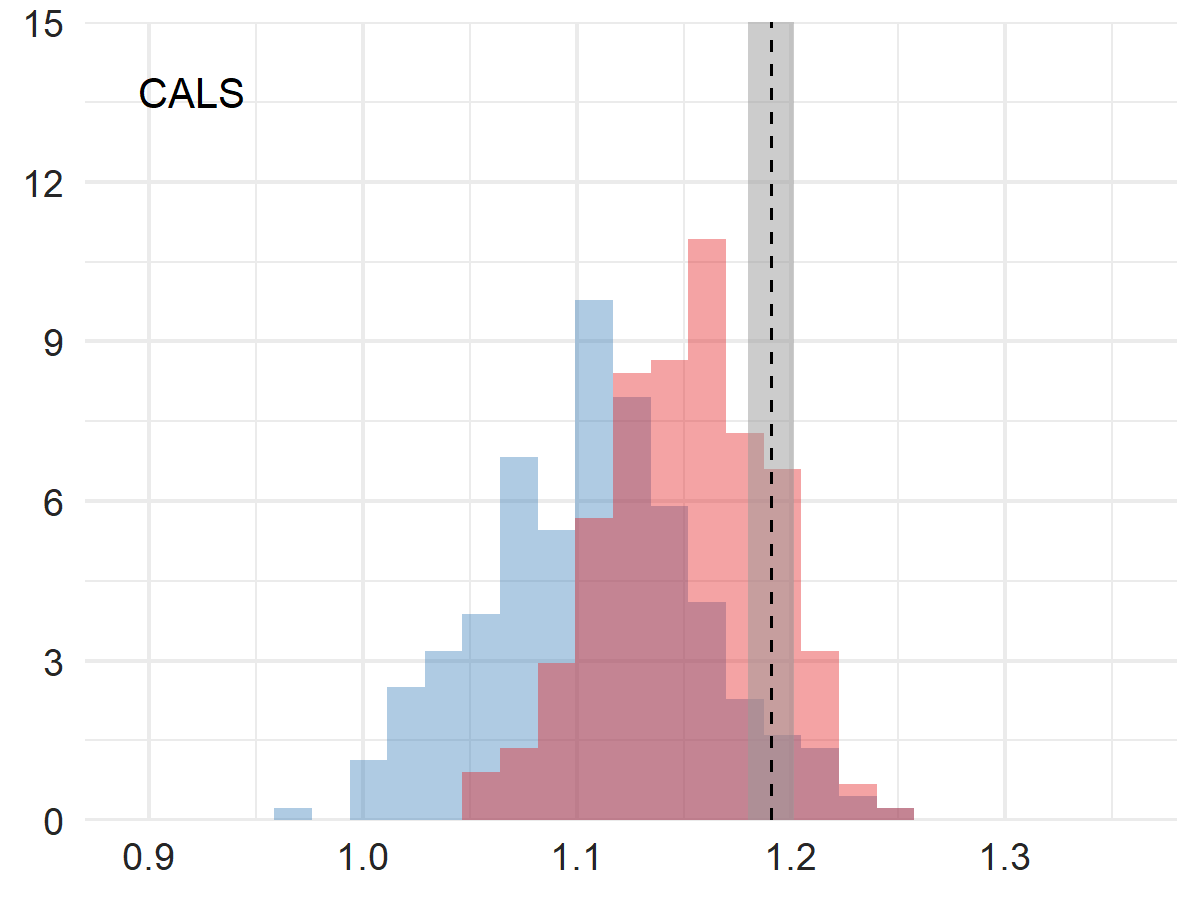

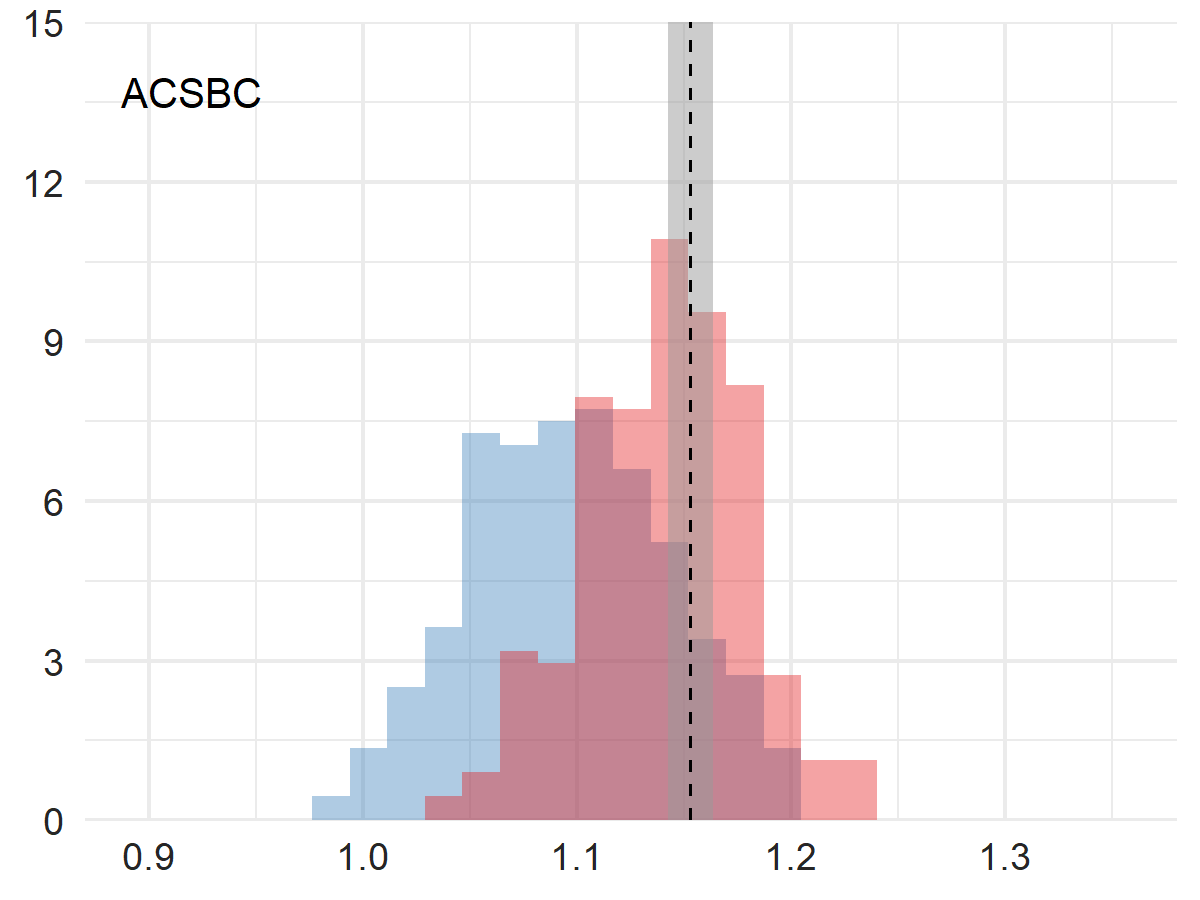

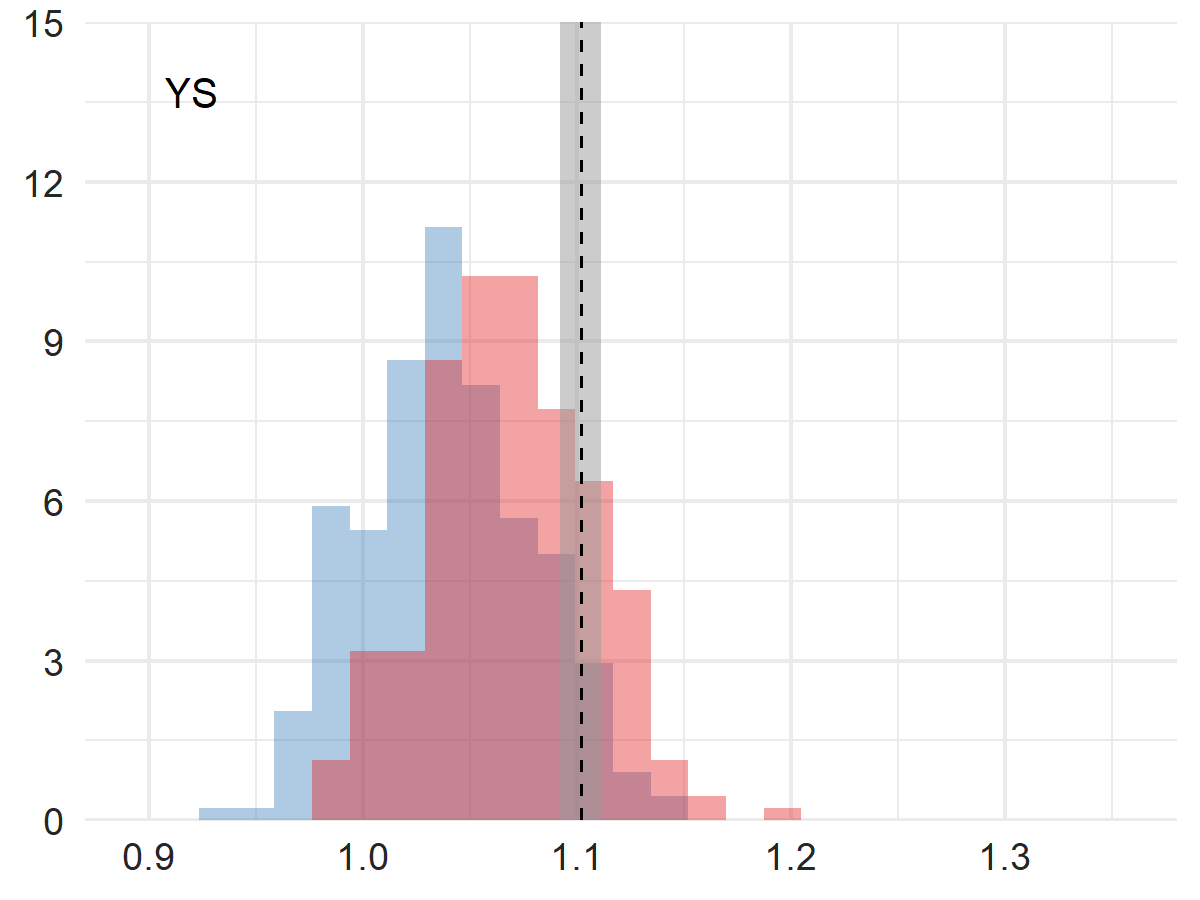


**Suppl. Figure 4**: Kaplan-Meier curves comparing the upper and lower quartiles of the pseudoscales burden in their effect on the rate of the outcome. For each outcome, the top panel shows the curves for the models adjusted for confounders and the bottom panel shows the curves for the unadjusted models. Years are depicted on the x-axis and the proportion of participants that experienced the outcome is depicted on the y-axis. Note that the curves are not necessarily monotonic due to the pooling and smoothing (see **Suppl. Text 2**).

*Death*


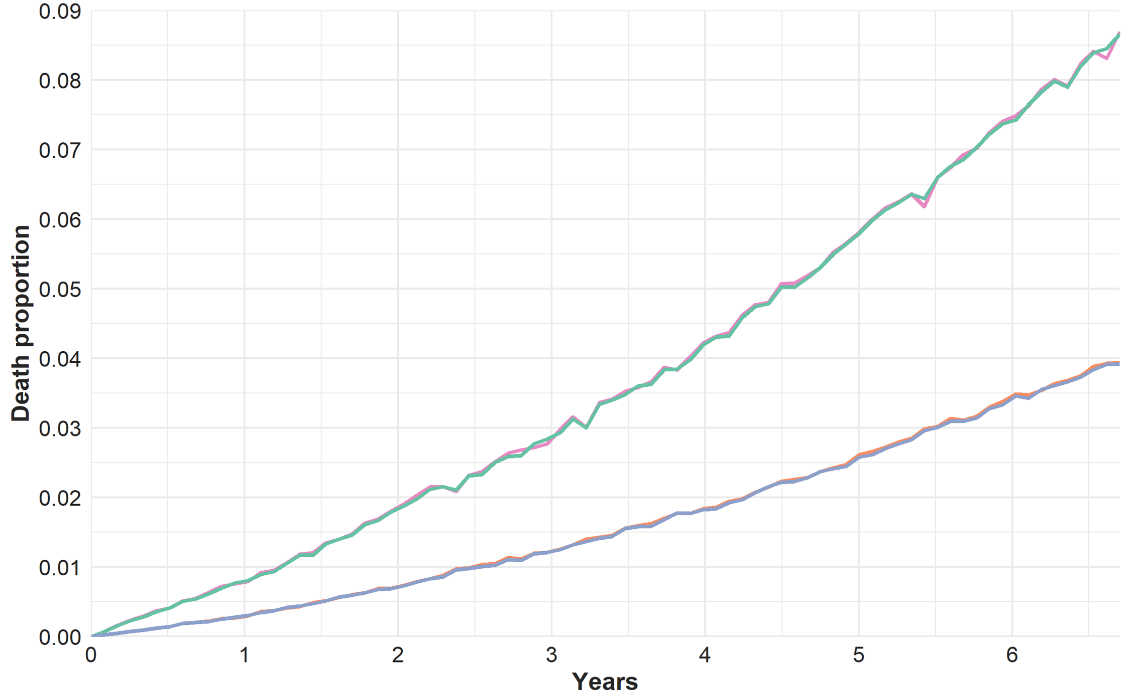

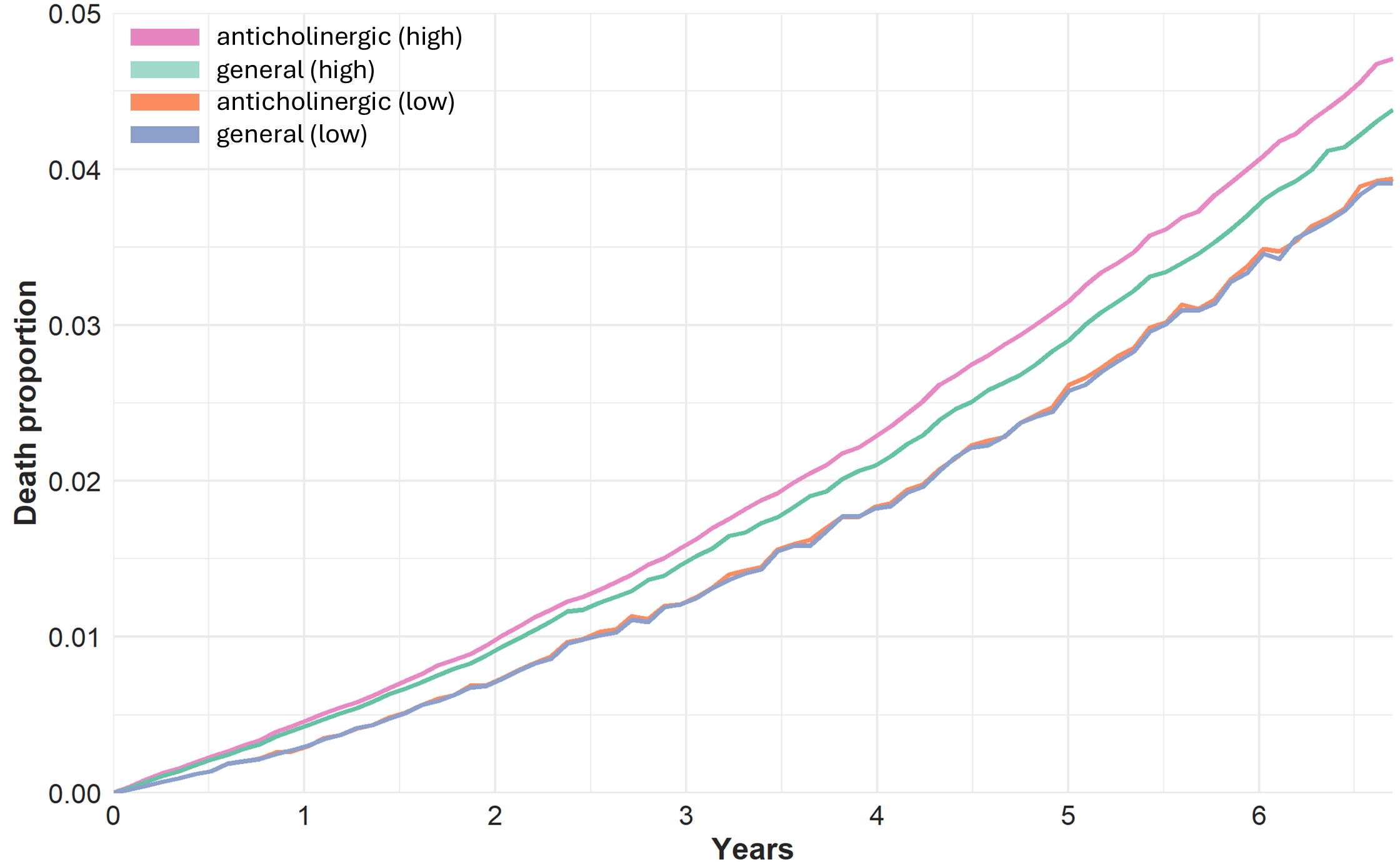


**adjusted**

**unadjusted**

*Dementia*


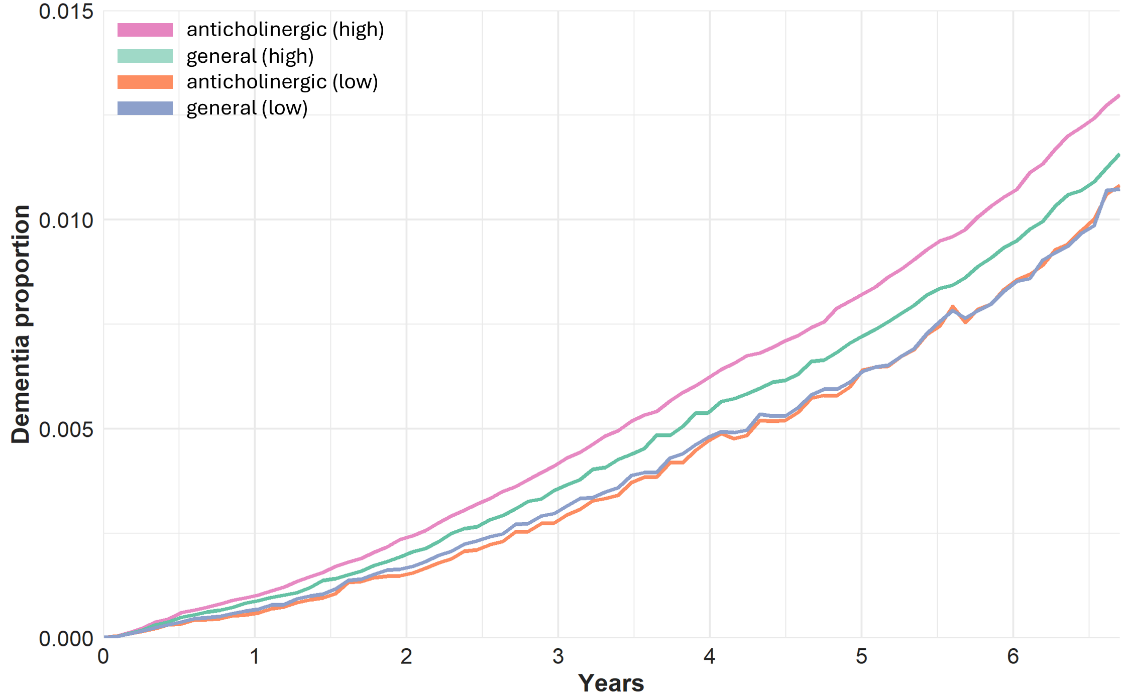

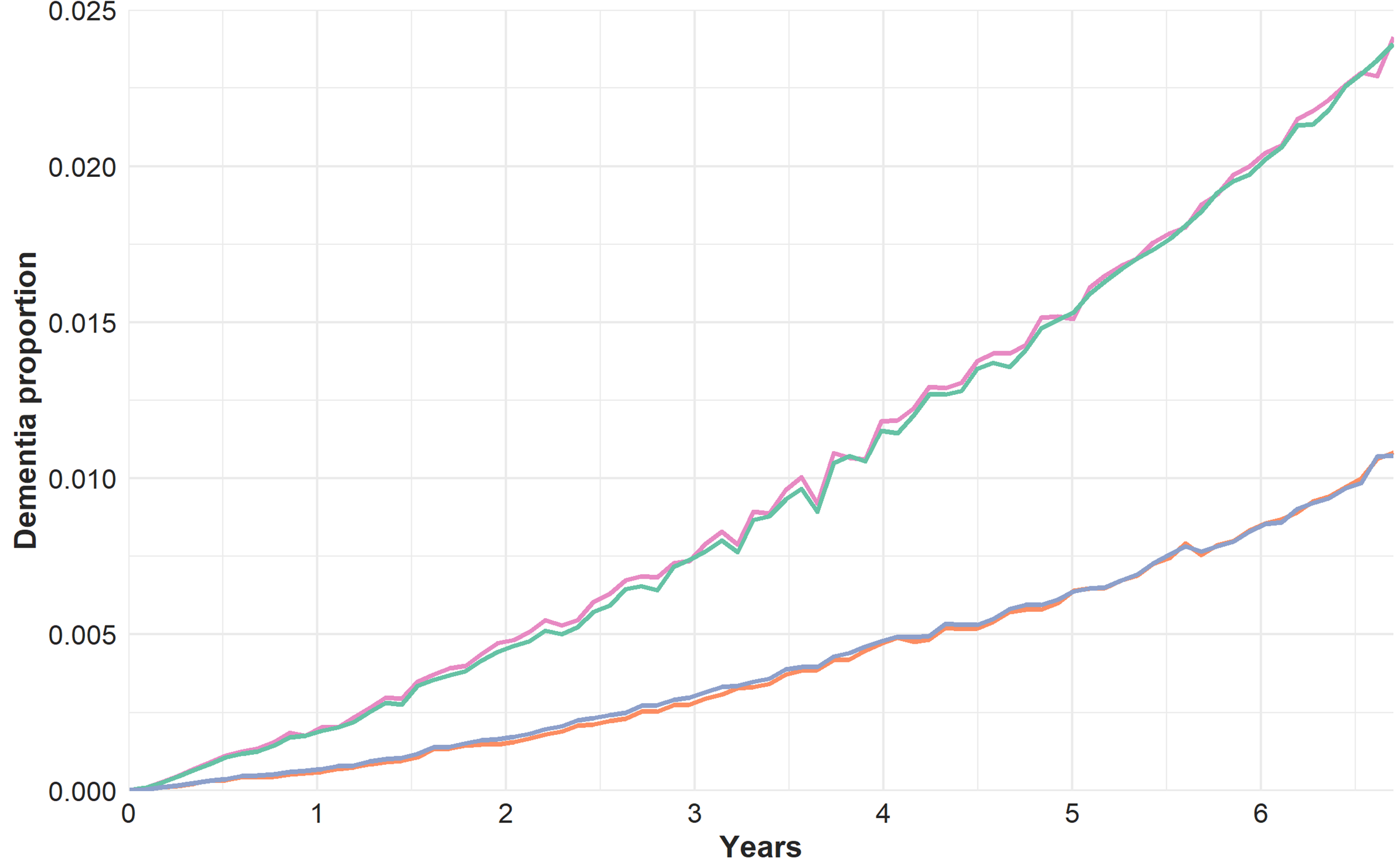


**adjusted**

**unadjusted**

*Delirium*


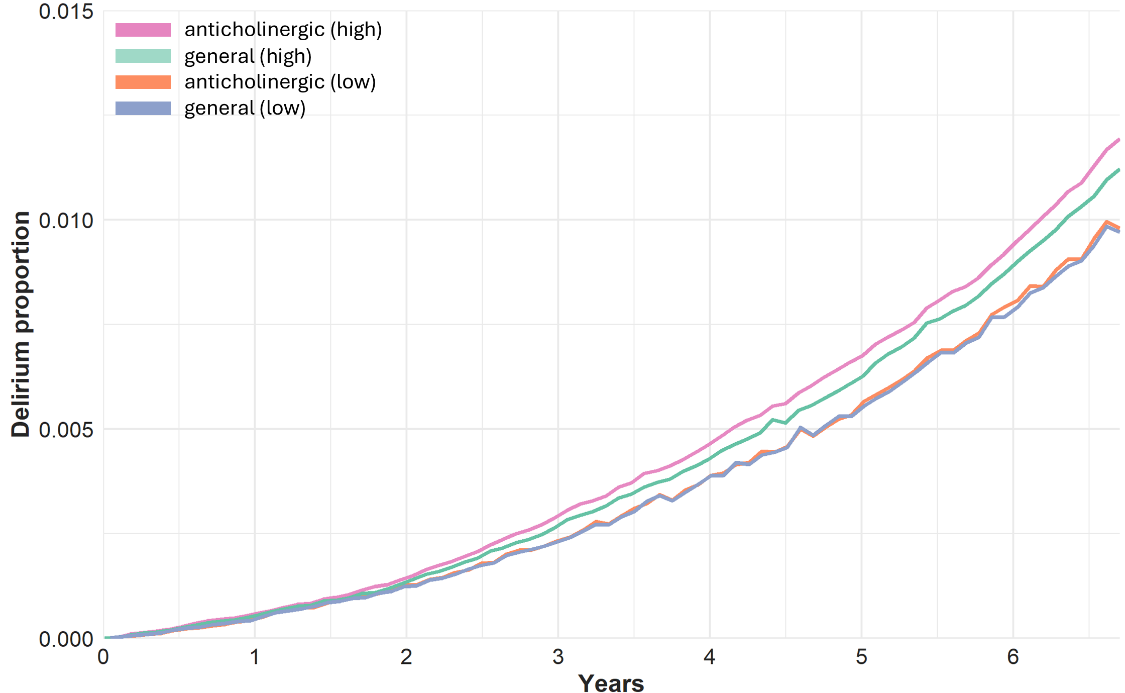

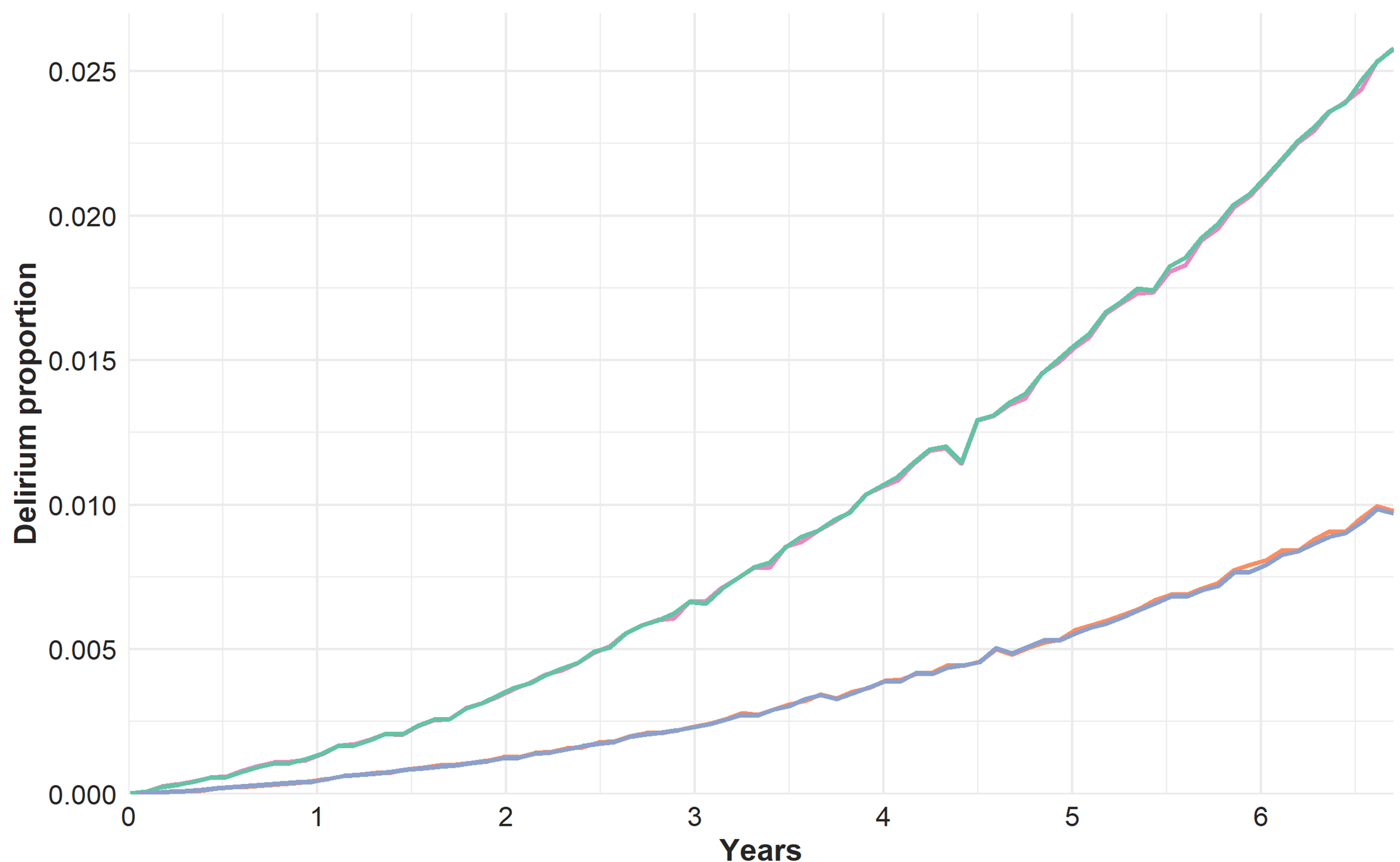


**adjusted**

**unadjusted**

**Supplementary references**

1 Boustani, M., Campbell, N., Munger, S., Maidment, I. & Fox, C. Impact of anticholinergics on the aging brain: A review and practical application. *Aging Health* **4**, 311–320 (2008). https://doi.org/10.2217/1745509X.4.3.311

2 Carnahan, R. M., Lund, B. C., Perry, P. J., Pollock, B. G. & Culp, K. R. The Anticholinergic Drug Scale as a measure of drug-related anticholinergic burden: associations with serum anticholinergic activity. *J Clin Pharmacol* **46**, 1481–1486 (2006). https://doi.org/10.1177/0091270006292126

3 Mur, J., Marioni, R. E., Russ, T. C., Muniz-Terrera, G. & Cox, S. R. Anticholinergic burden in middle and older age is associated with lower cognitive function, but not with brain atrophy. *Br J Clin Pharmacol* **89**, 2224–2235 (2023). https://doi.org/10.1111/bcp.15698

4 Summers, W. K. A clinical method of estimating risk of drug induced delirium. *Life Sci* **22**, 1511–1516 (1978). https://doi.org/10.1016/0024-3205(78)90006-1

5 Han, L., McCusker, J., Cole, M. *et al.* Use of medications with anticholinergic effect predicts clinical severity of delirium symptoms in older medical inpatients. *Archives of Internal Medicine* **161**, 1099–1105 (2001). https://doi.org/10.1001/archinte.161.8.1099

6 Ancelin, M. L., Artero, S., Portet, F. *et al.* Non-degenerative mild cognitive impairment in elderly people and use of anticholinergic drugs: longitudinal cohort study. *BMJ* **332**, 455–459 (2006). https://doi.org/10.1136/bmj.38740.439664.DE

7 Chew, M. L., Mulsant, B. H., Pollock, B. G. *et al.* Anticholinergic activity of 107 medications commonly used by older adults. *J Am Geriatr Soc* **56**, 1333–1341 (2008). https://doi.org/10.1111/j.1532-5415.2008.01737.x

8 Cancelli, I., Valentinis, L., Merlino, G., Valente, M. & Gigli, G. L. Drugs with anticholinergic properties as a risk factor for psychosis in patients affected by Alzheimer's disease. *Clinical Pharmacology and Therapeutics* **84**, 63–68 (2008). https://doi.org/10.1038/sj.clpt.6100435

9 Rudolph, J. L., Salow, M. J., Angelini, M. C. & McGlinchey, R. E. The anticholinergic risk scale and anticholinergic adverse effects in older persons. *Archives of Internal Medicine* **168**, 508–513 (2008). https://doi.org/10.1001/archinternmed.2007.106

10 Ehrt, U., Broich, K., Larsen, J. P., Ballard, C. & Aarsland, D. Use of drugs with anticholinergic effect and impact on cognition in Parkinson's disease: a cohort study. *J Neurol Neurosurg Psychiatry* **81**, 160–165 (2010). https://doi.org/10.1136/jnnp.2009.186239

11 Sittironnarit, G., Ames, D., Bush, A. I. *et al.* Effects of anticholinergic drugs on cognitive function in older Australians: results from the AIBL study. *Dement Geriatr Cogn Disord* **31**, 173–178 (2011). https://doi.org/10.1159/000325171

12 Sumukadas, D., McMurdo, M. E., Mangoni, A. A. & Guthrie, B. Temporal trends in anticholinergic medication prescription in older people: repeated cross-sectional analysis of population prescribing data. *Age Ageing* **43**, 515–521 (2014). https://doi.org/10.1093/ageing/aft199

13 Durán, C. E., Azermai, M. & Vander Stichele, R. H. Systematic review of anticholinergic risk scales in older adults. *Eur J Clin Pharmacol* **69**, 1485–1496 (2013). https://doi.org/10.1007/s00228-013-1499-3

14 Hefner, G., Shams, M. E. E., Wenzel-Seifert, K. *et al.* Rating The Delirogenic Potential of Drugs for Prediction of Side Effects in Elderly Psychiatric Inpatients. *Jacobs Journal of Pharmacology and Pharmacovigilance* **1** (2015).

15 Nguyen, P. V., Pelletier, L., Payot, I. & Latour, J. Drug Delirium Scale (DDS): A Tool to Evaluate Drugs as a Risk Factor for Delirium. *International Journal of Innovative Research in Medical Science* **1** (2016). https://doi.org/10.23958/ijirms/vol01-i06/02

16 Bishara, D., Harwood, D., Sauer, J. & Taylor, D. M. Anticholinergic effect on cognition (AEC) of drugs commonly used in older people. *Int J Geriatr Psychiatry* **32**, 650–656 (2017). https://doi.org/10.1002/gps.4507

17 Briet, J., Javelot, H., Heitzmann, E. *et al.* The anticholinergic impregnation scale: Towards the elaboration of a scale adapted to prescriptions in French psychiatric settings. *Therapie* **72**, 427–437 (2017). https://doi.org/10.1016/j.therap.2016.12.010

18 Kiesel, E. K., Hopf, Y. M. & Drey, M. An anticholinergic burden score for German prescribers: score development. *BMC Geriatr* **18**, 239 (2018). https://doi.org/10.1186/s12877-018-0929-6

19 Nery, R. T. & Reis, A. M. M. Development of a Brazilian anticholinergic activity drug scale. *Einstein (Sao Paulo)* **17**, eAO4435 (2019). https://doi.org/10.31744/einstein_journal/2019AO4435

20 Jun, K., Hwang, S., Ah, Y. M., Suh, Y. & Lee, J. Y. Development of an Anticholinergic Burden Scale specific for Korean older adults. *Geriatr Gerontol Int* **19**, 628–634 (2019). https://doi.org/10.1111/ggi.13680

21 Kable, A., Fullerton, A., Fraser, S. *et al.* Comparison of Potentially Inappropriate Medications for People with Dementia at Admission and Discharge during An Unplanned Admission to Hospital: Results from the SMS Dementia Study. *Healthcare (Basel)* **7** (2019). https://doi.org/10.3390/healthcare7010008

22 Ramos, H., Moreno, L., Pérez-Tur, J. *et al.* CRIDECO Anticholinergic Load Scale: An Updated Anticholinergic Burden Scale. Comparison with the ACB Scale in Spanish Individuals with Subjective Memory Complaints. *J Pers Med* **12** (2022). https://doi.org/10.3390/jpm12020207

23 Al Rihani, S. B., Deodhar, M., Darakjian, L. I. *et al.* Quantifying Anticholinergic Burden and Sedative Load in Older Adults with Polypharmacy: A Systematic Review of Risk Scales and Models. *Drugs Aging* **38**, 977–994 (2021). https://doi.org/10.1007/s40266-021-00895-x

24 Yamada, S., Mochizuki, M., Chimoto, J. *et al.* Development of a pharmacological evidence-based anticholinergic burden scale for medications commonly used in older adults. *Geriatr Gerontol Int* (2023). https://doi.org/10.1111/ggi.14619

25 Textor, J., van der Zander, B., Gilthorpe, M. S., Liśkiewicz, M. & Ellison, G. T. Robust causal inference using directed acyclic graphs: the R package 'dagitty'. *Int J Epidemiol* **45**, 1887–1894 (2016). https://doi.org/10.1093/ije/dyw341

26 UK Biobank. UK Biobank Primary Care Linked Data Version 1.0. (2019). <https://biobank.ndph.ox.ac.uk/showcase/ukb/docs/primary_care_data.pdf>

27 Darke, P., Cassidy, S., Catt, M. *et al.* Curating a longitudinal research resource using linked primary care EHR data-a UK Biobank case study. *J Am Med Inform Assoc* **29**, 546–552 (2022). https://doi.org/10.1093/jamia/ocab260

28 Townsend, P. Deprivation. *Journal of Social Policy* **16**, 125–146 (1987). https://doi.org/10.1017/s0047279400020341

29 Cox, S. R., Ritchie, S. J., Fawns-Ritchie, C., Tucker-Drob, E. M. & Deary, I. J. Structural brain imaging correlates of general intelligence in UK Biobank. *Intelligence* **76**, 101376 (2019). https://doi.org/10.1016/j.intell.2019.101376

30 Peters, R., Ee, N. Peters, J. *et al.* Air Pollution and Dementia: A Systematic Review. *J Alzheimers Dis* **70**, S145–S163 (2019). https://doi.org/10.3233/JAD-180631

31 Hanlon, P., Quinn, T. J., Gallacher, K I. *et al.* Assessing Risks of Polypharmacy Involving Medications With Anticholinergic Properties. *Ann Fam Med* **18**, 148–155 (2020). https://doi.org/10.1370/afm.2501

32 Mur, J., Cox, S. R., Marioni, R. E., Muniz-Terrera, G. & Russ, T. C. Increase in anticholinergic burden from 1990 to 2015: Age-period-cohort analysis in UK biobank. *Br J Clin Pharmacol* (2021). https://doi.org/10.1111/bcp.15045

33 Brenowitz, W. D., Filshtein, T. J., Yaffe, K. *et al.* Association of genetic risk for Alzheimer disease and hearing impairment. *Neurology* **95**, e2225–e2234 (2020). https://doi.org/10.1212/WNL.0000000000010709

34 Dawes, P., Fortnum, H., Moore, D. R. *et al.* Hearing in middle age: a population snapshot of 40- to 69-year olds in the United Kingdom. *Ear Hear* **35**, e44–51 (2014). https://doi.org/10.1097/AUD.0000000000000010

35 Mur, J., Klee, M., Wright, H. R. *et al.* A hypothetical intervention on the use of hearing aids for the risk of dementia in people with hearing loss in UK Biobank. *Am J Epidemiol* (2024). https://doi.org/10.1093/aje/kwae452

36 Elovainio, M., Hakulinen, C., Pulkki-Räback, L. *et al.* Contribution of risk factors to excess mortality in isolated and lonely individuals: an analysis of data from the UK Biobank cohort study. *Lancet Public Health* **2**, e260–e266 (2017). https://doi.org/10.1016/S2468-2667(17)30075-0

37 Sävje, F., Higgins, M. J. & Sekhon, J. S. Generalized Full Matching. *Political Analysis* **29**, 423–447 (2020). https://doi.org/10.1017/pan.2020.32

38 Sävje, F., Sekhon, J. S. & Higgins, M. J. Quick Generalized Full Matching (2023). <https://cran.r-project.org/web//packages/quickmatch/quickmatch.pdf>

39 Ho, D. E., Imai, K., King, G. & Stuart, E. A. MatchIt: nonparametric preprocessing for parametric causal inference. *Journal of Statistical Software* **42** (2011). https://doi.org/10.18637/jss.v042.i08

40 Fine, J. P. & Gray, R. J. A Proportional Hazards Model for the Subdistribution of a Competing Risk. *J Am Stat Assoc* **94** (1999). https://doi.org/10.1080/01621459.1999.10474144

1. Centre for Clinical Brain Sciences, University of Edinburgh, Edinburgh, United Kingdom [↑](#footnote-ref-1)
2. Department of Social Sciences, Institute for Research on Socio-Economic Inequality (IRSEI), University of Luxembourg, Esch-sur-Alzette, Luxembourg [↑](#footnote-ref-2)
3. Global Brain Health Institute, University of California San Francisco, San Francisco, California, United States [↑](#footnote-ref-3)
4. Heritage College of Osteopathic Medicine, Ohio University, Athens, Ohio, United States
   *Corresponding author; email: jure.mur@uni.lu [↑](#footnote-ref-4)
5. For general polypharmacy, this was a list of all unique medicines prescribed to the sample in the period of interest (*n*=525 for the year 2015). For anticholinergic polypharmacy, it was a list of all unique medicines prescribed to the sample in the period of interest that were assigned an anticholinergic potency >0 by at least one ABS (*n*=214 for the year 2015). The list of drugs included only generic names, for example alimemazine, aspirin, haloperidol, etc. [↑](#footnote-ref-5)
6. Where *n* is the number of drugs with a potency>0 that are to be included in the pseudoscale. In across-sampling, *n* varied from pseudoscale to pseudoscale, with the range of possible values determined by the range observed among the 23 ABS used in the present study (n=15-150). In within-sampling, a set of pseudoscales was constructed for each ABS; *n* was invariant within a set and equal to the number of drugs included in the ABS. [↑](#footnote-ref-6)
7. In across-sampling, *s* could take the values 4, 3, 2, 1, and 0.5, corresponding to the scores found across the 23 ABS. The probability of assigning *s* to a drug depended on the proportion of drugs with a score>0 that were assigned that score among the 23 ABS. In practice, each drug had the following probability of being assigned a certain value of *s*: 0.017 (*n*=4), 0.25 (*n*=3), 0.20 (*n*=2), 0.53 (*n*=1), 0.009 (*n*=0.5). In within-sampling, for any ABS, the numbers of drugs assigned each potency score s were invariant across all pseudoscales in the set and equal to the number of drugs with those potency scores in the ABS. [↑](#footnote-ref-7)
